# Supplementary material for: A fluorogenic probe for predicting treatment response in non-small cell lung cancer with EGFR-activating mutations
Source: Nat Commun. 2022 Nov 14;13:6944. doi: 10.1038/s41467-022-34627-5 (PMC9663578; doi:10.1038/s41467-022-34627-5)
Supplement: Supplementary file 1 — Supplementary Information [file 41467_2022_34627_MOESM1_ESM.pdf]

Supplementary Information

A fluorogenic probe for predicting treatment response in non-small cell lung cancer with *EGFR*-activating mutations

Hui Deng[1,2,3], Qian Lei[1,2,3], Chengdi Wang[1], Zhoufeng Wang[1,3], Hai Chen[2,3], Gang Wang[2], Na Yang[2], Dan Huang[4], Quanwei Yu[2], Mengling Yao[3], Xue Xiao[3], Guonian Zhu[3], Cheng Cheng[3], Yangqian Li[3], Feng Li[4], Panwen Tian[1], and Weimin Li[1,2,3]

Supplementary Figures

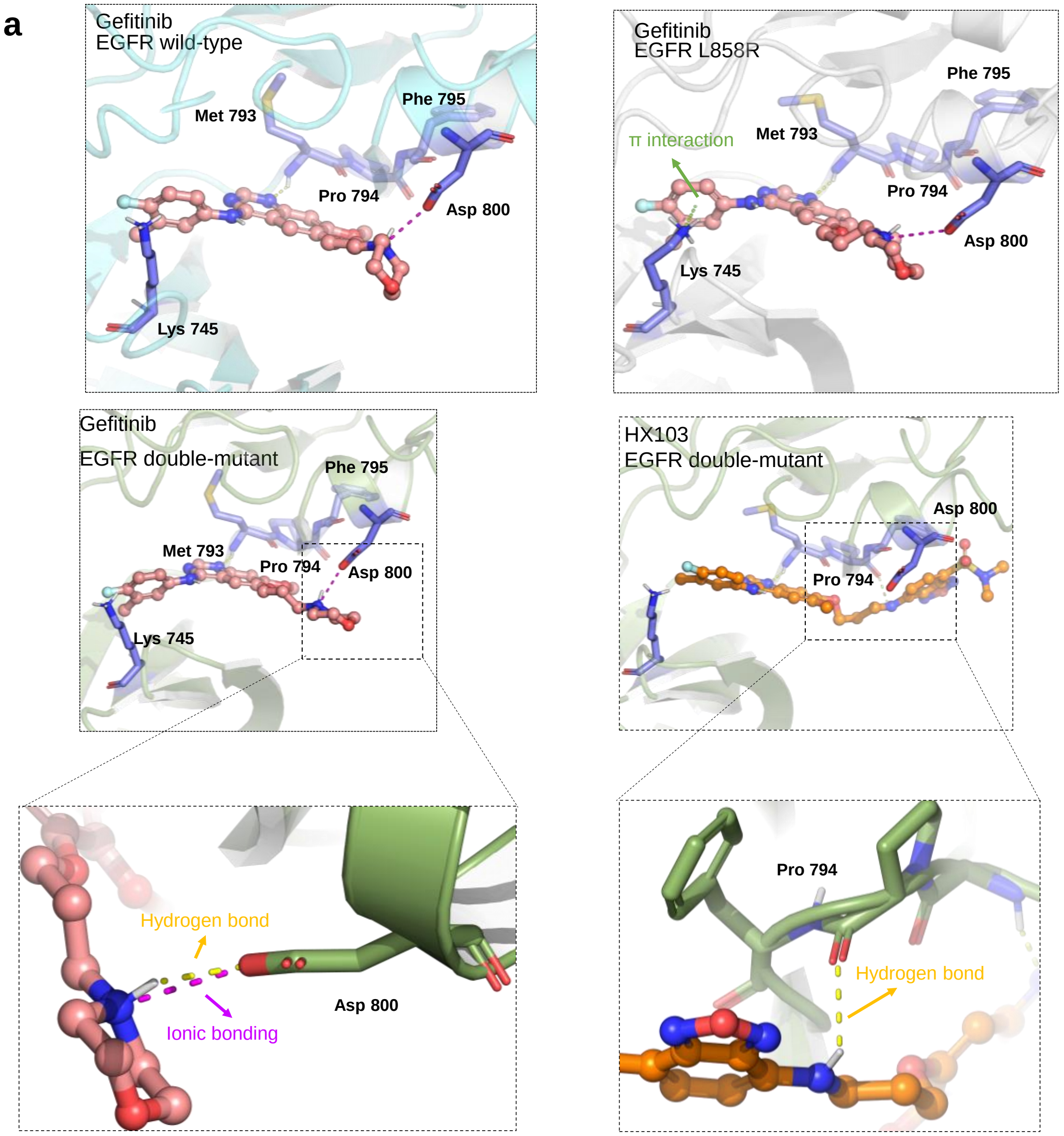

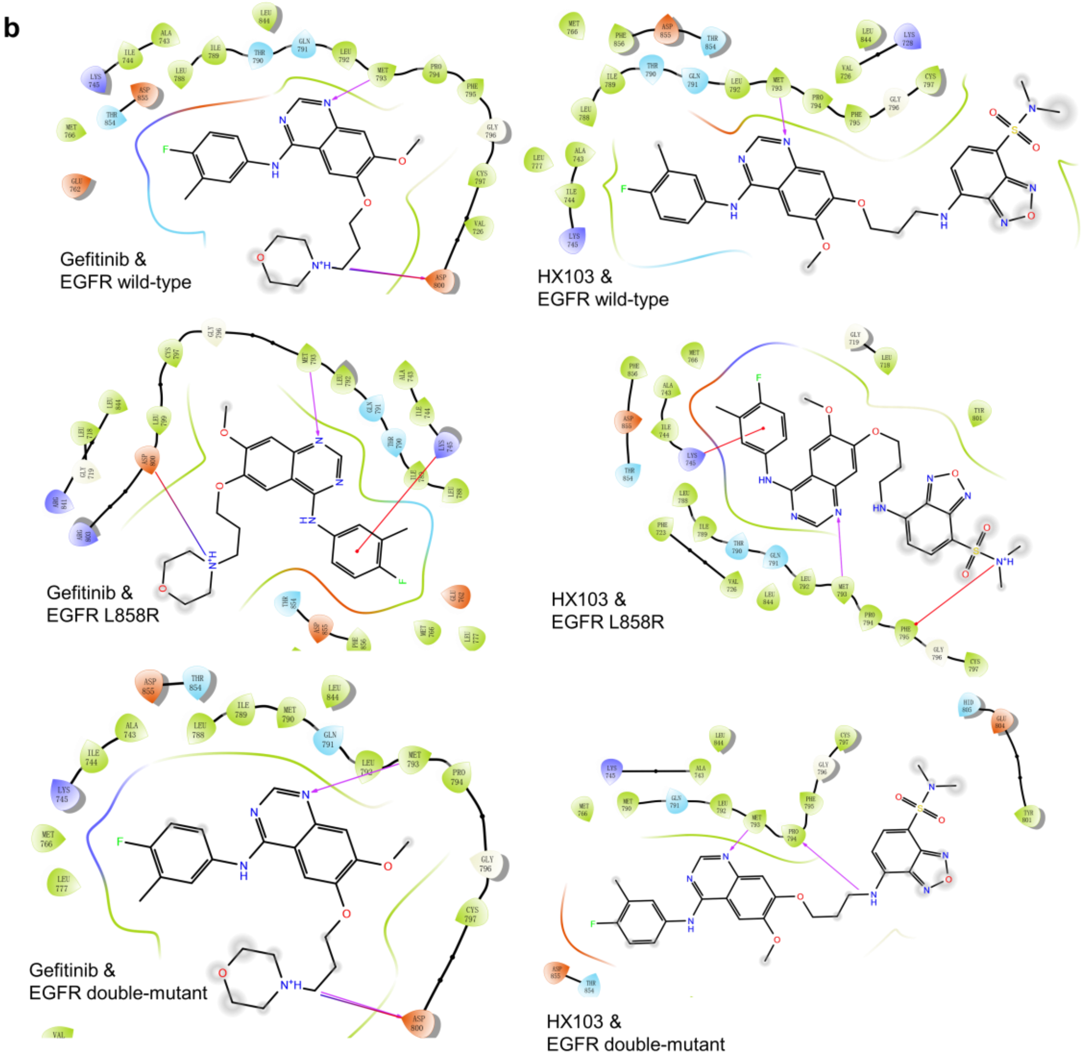

Supplementary Figure 1. Docking results of gefitinib and HX103 to EGFR. (**a**) Predicted binding modes of gefitinib with the EGFR wild-type (PDB: 4WKQ), EGFR L858R mutant (PBD: 4LQM) and EGFR L858R/T790M (PDB: 5EDP); HX103 showing the interaction with Pro794 in EGFR double-mutant. The hydrogen bond is shown as yellow, while ionic bonding is shown as pink and π interaction is shown in green. (**b**) The 2D interaction diagram between gefitinib/HX103 and EGFRs (wild-type, L858R, and double mutant).

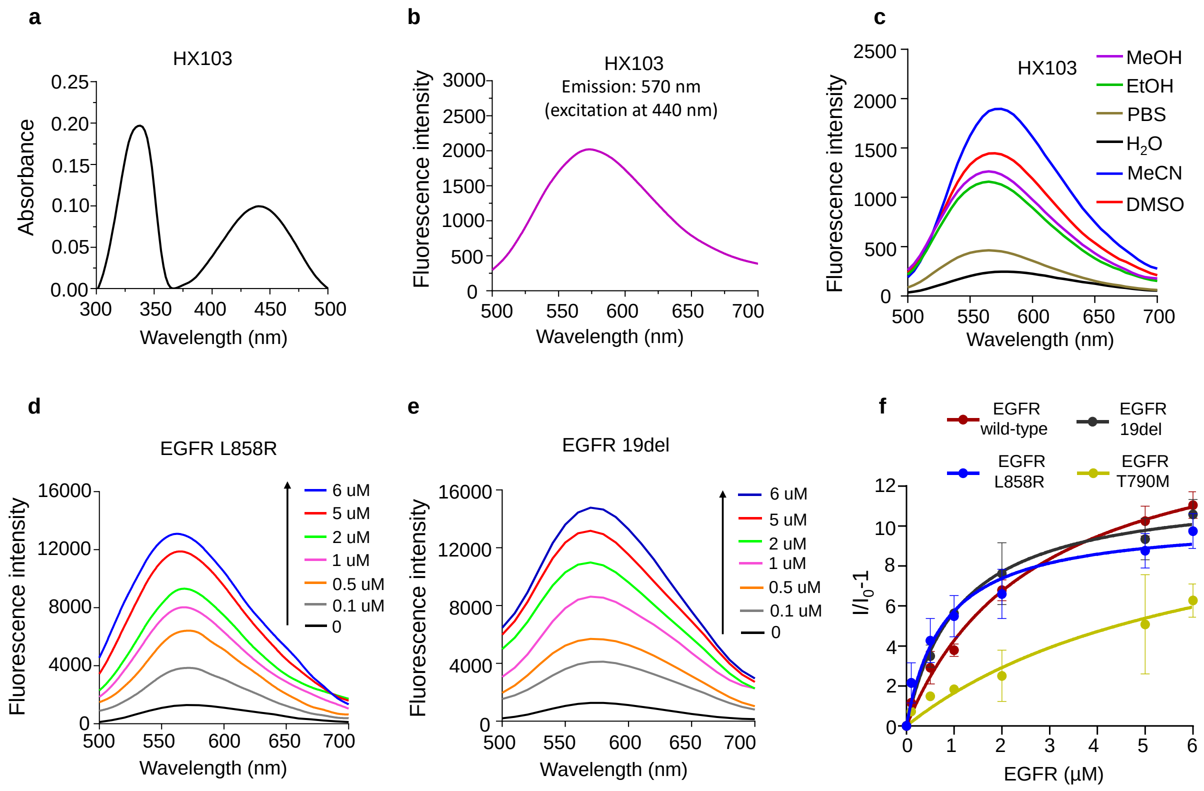

Supplementary Figure 2. Fluorescence turn-on response of HX103 on EGFR. (**a**, **b**) Absorption and emission spectra of HX103 (5 μM) in DMSO. Excitation wavelength for emission fluorescence spectra was 440 nm. (**c**) Fluorescence spectra of HX103 (5 μM) in different solvents. (**d**) Fluorescence spectra change of HX103 (5 μM) with increasing concentration of EGFR L858R mutant (0 to 6 μM) in PBS buffer. (**e**) Fluorescence spectra change of HX103 (5 μM) with increasing concentration of EGFR 19del (0 to 6 μM) in PBS buffer. (**f**) Titration curve of HX103 (5 μM) with increasing concentration of EGFRs (0 to 6 μM). The apparent binding affinity (K_d) of HX103 to EGFR wild-type, L858R, 19del and T790M mutants are 2.7 ± 0.4 μM, 0.8 ± 0.3 μM, 1.1 ± 0.2 μM and 6.6 ± 4.6 μM, respectively. The fluorescence titration curve was fitted on one-site specific binding model. I and I_0 represent the intensities of HX103 at 570 nm in the presence and absence of protein. Data represent average values ± SD, n = 3 independent experiments per group. Source data are provided as a Source Data file.

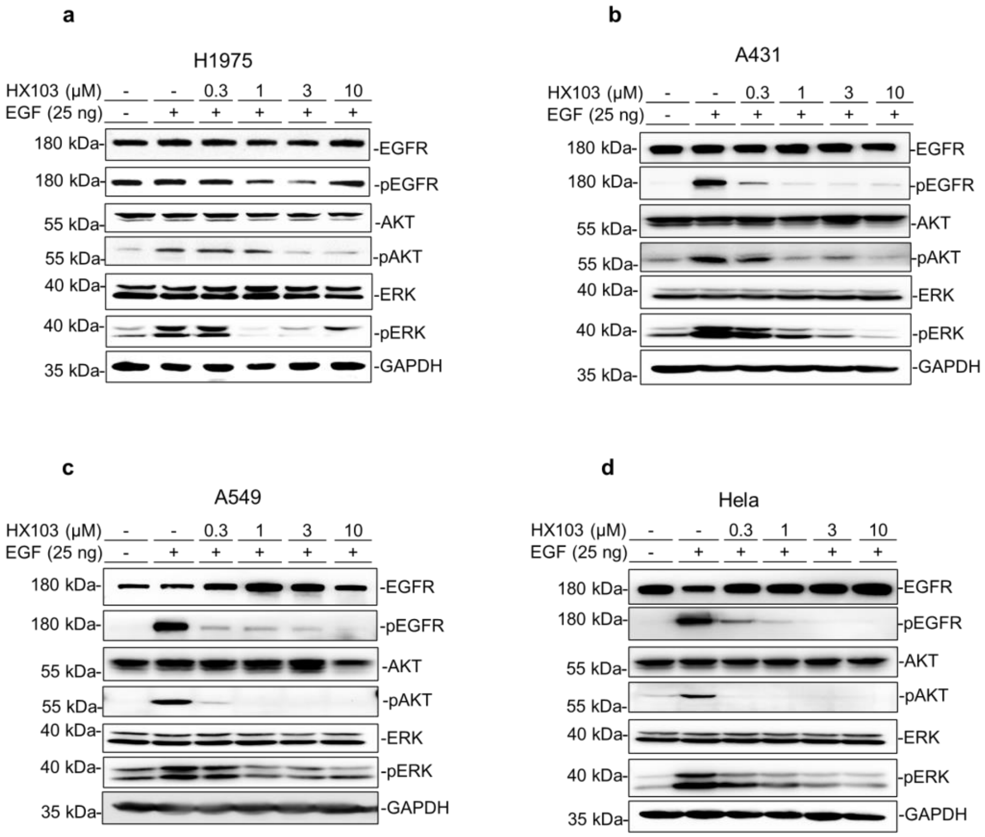

Supplementary Figure 3. Molecular pharmacology of HX103 on EGFR-signaling pathway. (**a**) The impacts of HX103 (0 to 10 μM) on the activation of EGFR and its downstream AKT and ERK1/2 phosphorylation levels were measured by Western blot in H1975 harboring EGFR mutations. (**b-d**) The impacts of HX103 (0 to 10 μM) on the activation of EGFR EGF (25 ng/mL) and its downstream AKT and ERK1/2 phosphorylation levels were measured by Western blot in cancer cells with EGFR wild-type. Uncropped blots are in Source Data. Three biological independent experiments were repeated with similar results.

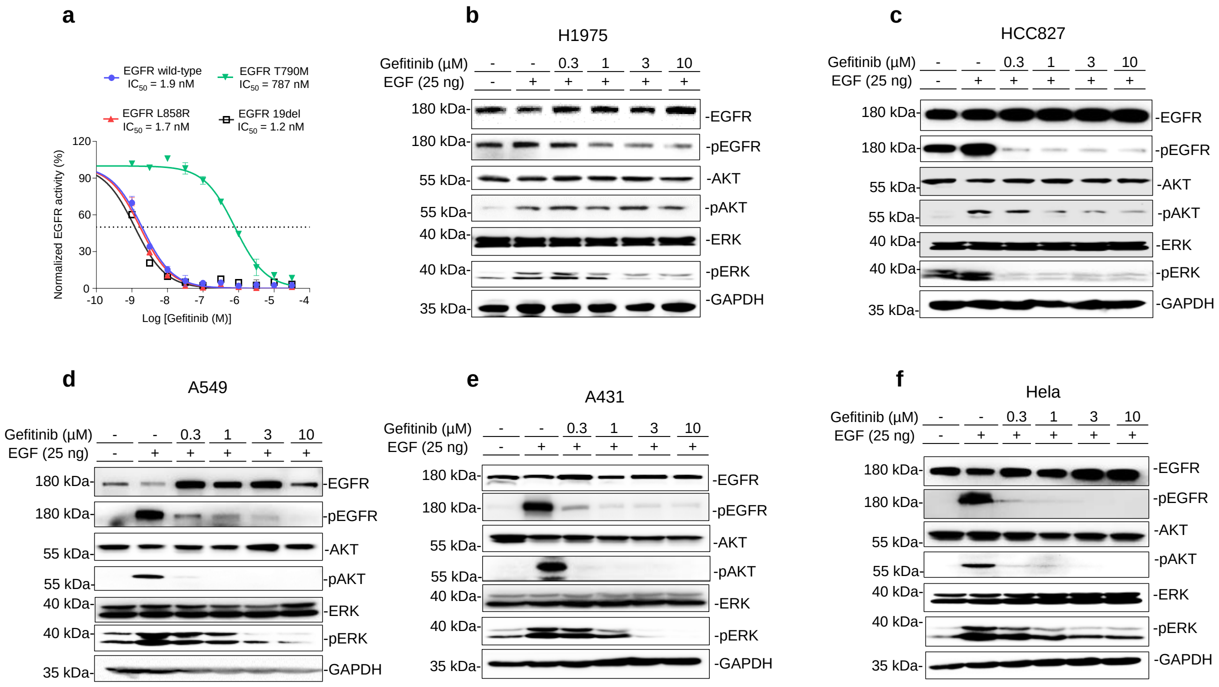

Supplementary Figure 4. Molecular pharmacology of gefitinib on EGFR. (**a**) Kinase inhibitory activity of gefitinib on recombinant human EGFR wild-type, L858R, 19del and T790M determined by a fluorescent mobility-shift assay. Data represent average values ± SEM, n = 3 independent experiments per group. (**b-f**) The impacts of gefitinib (0 to 10 μM) on EGFR activation EGF (25 ng/mL) and its downstream AKT and ERK1/2 phosphorylation levels were tested in H1975 (b), HCC827 (c), A549 (d), A431 (e) and Hela (f). Uncropped blots are in Source Data. Three biological independent experiments were repeated with similar results. Source data are provided as a Source Data file.

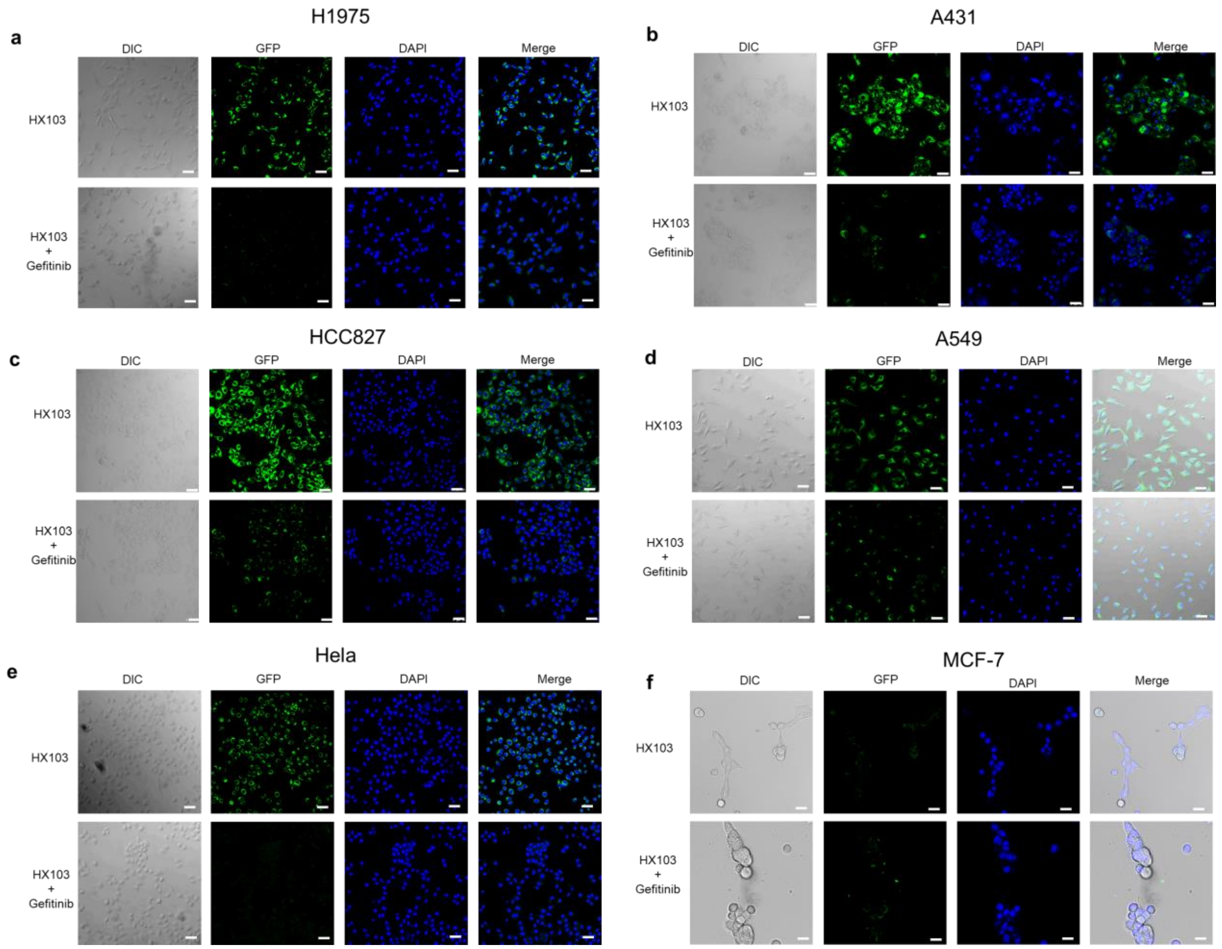

Supplementary Figure 5. Fluorescence imaging of cancer cells with HX103. (**a-f**) Visualization of HX103 (5 µM, 30 min) uptake in H1975 (a), A431 (b), HCC827 (c), A549 (d), Hela (e) and MCF-7 (f) by fluorescence confocal microscopy. Scale bar, 50 µm. The uptake of HX103 in HCC827, H1975, A549, A431 and Hela cells can be blocked by preincubation with gefitinib (50 µM). Three biological independent experiments were repeated with similar results.

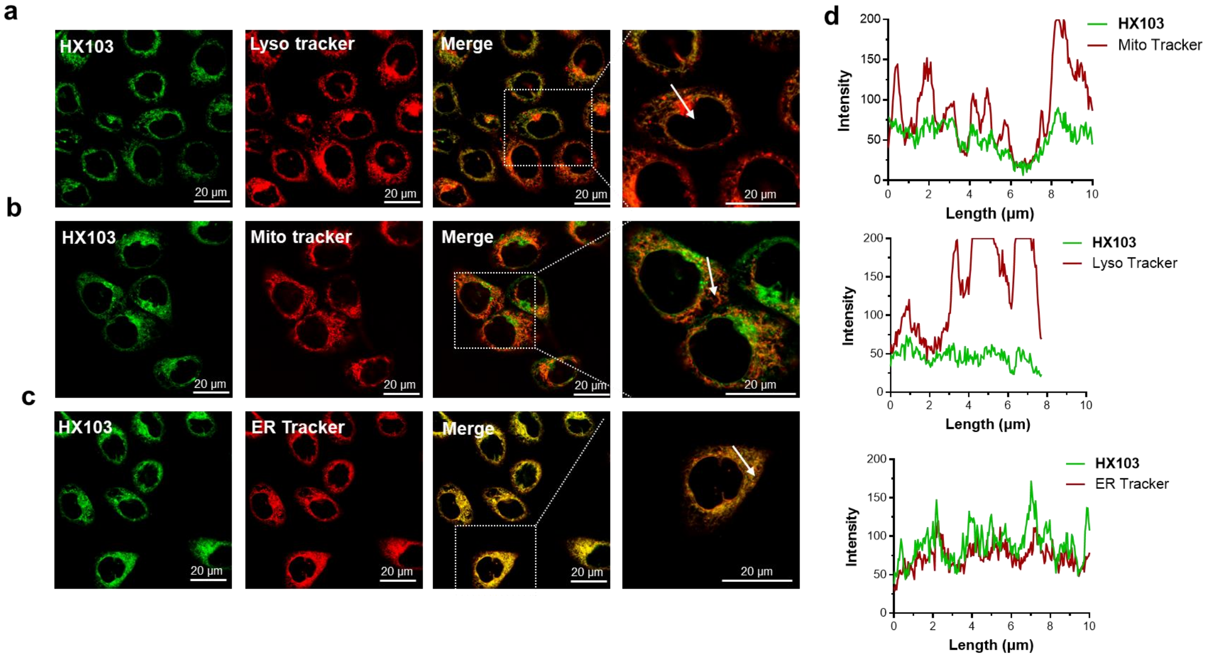

Supplementary Figure 6. Application of HX103 in living cells. (**a-c**) Subcellular co-localization of HX103 with Lyso tracker (a), Mito tracker (b) or ER tracker (c). H1975 cells were treated with HX103 (5 µM, 0.5 h) and stained with Lyso tracker (100 µM, 30 min), Mito tracker (100 nM, 15 min) or ER tracker (500 nM, 15 min). After a PBS washing, the cells were used for confocal microscopy. Scale bar, 20 µm. Three biological independent experiments were repeated with similar results. (**d**) The mean fluorescence intensity analysis along white arrows in the overlay image in (a-c). Source data are provided as a Source Data file.

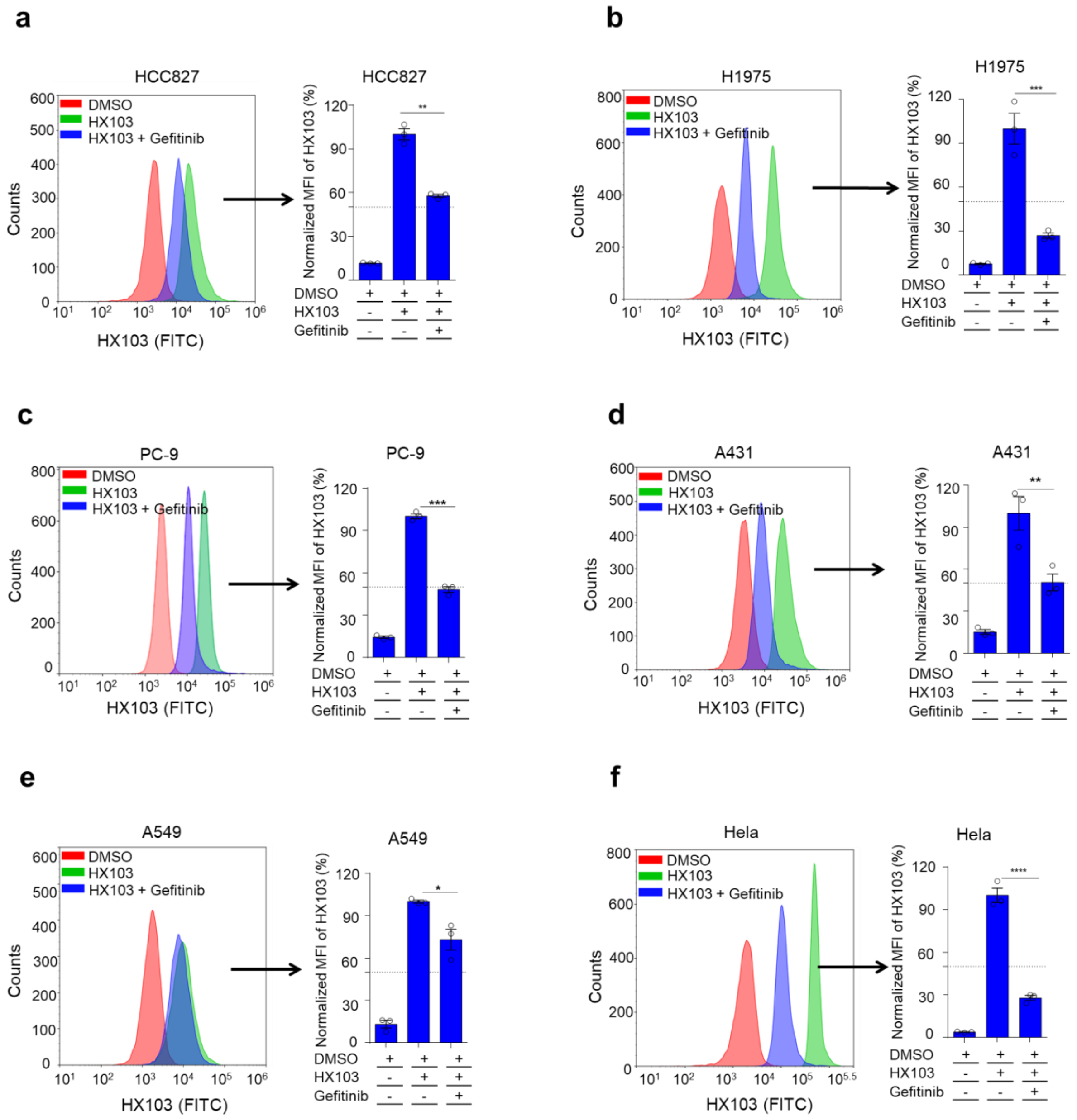

Supplementary Figure 7. Competitive flow cytometric experiments of HX103 with EGFR-TKI (gefitinib). (**a-f**) Representative histograms and bar charts showing fluorescence intensity differences between untreated (DMSO), HX103 (5 μM)-treated cells and that with gefitinib preincubation (50 μM) in HCC827 (a), H1975 (b), PC-9 (c), A431 (d), A549 (e) and Hela (f). Data represent average values ± SEM, n = 3 independent experiments per group. The statistical *P* values were calculated by the two-tailed Student's test, $^{*}P$<0.05, $^{**}P$<0.01, $^{***}P$<0.001 for gefitinib pre-treated vs. only HX103-treated group. *P* = 0.002 (HCC827), *P* = 0.006 (A431), *P* = 0.022 (A549). Flow cytometric was performed to quantify antibody (#ab192982, Alex Fluor 647) labeled EGFR in cancer cell lines. Source data are provided as a Source Data file.

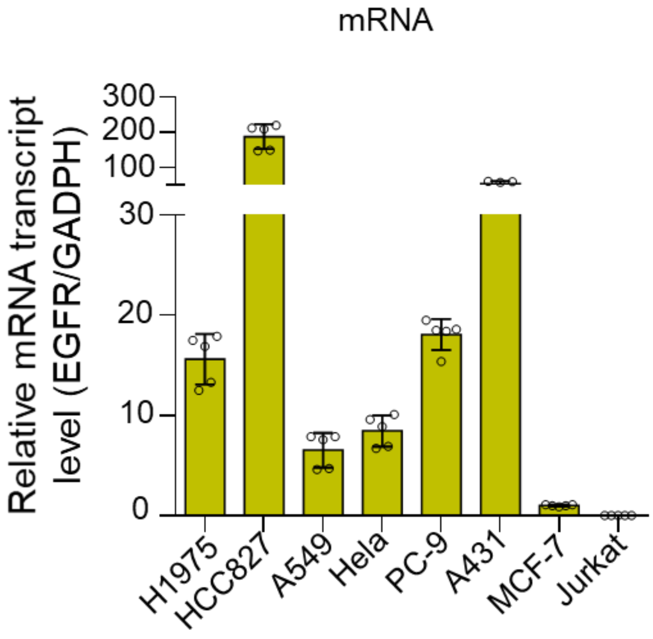

Supplementary Figure 8. qPCR analysis of EGFR/GAPDH in a panel of cancer cells. The ratio of EGFR/GAPDH in MCF-7 was set at 1 to normalize that of the remaining cell lines. Data represent average values ± SD, n = 5 independent experiments per group. Source data are provided as a Source Data file.

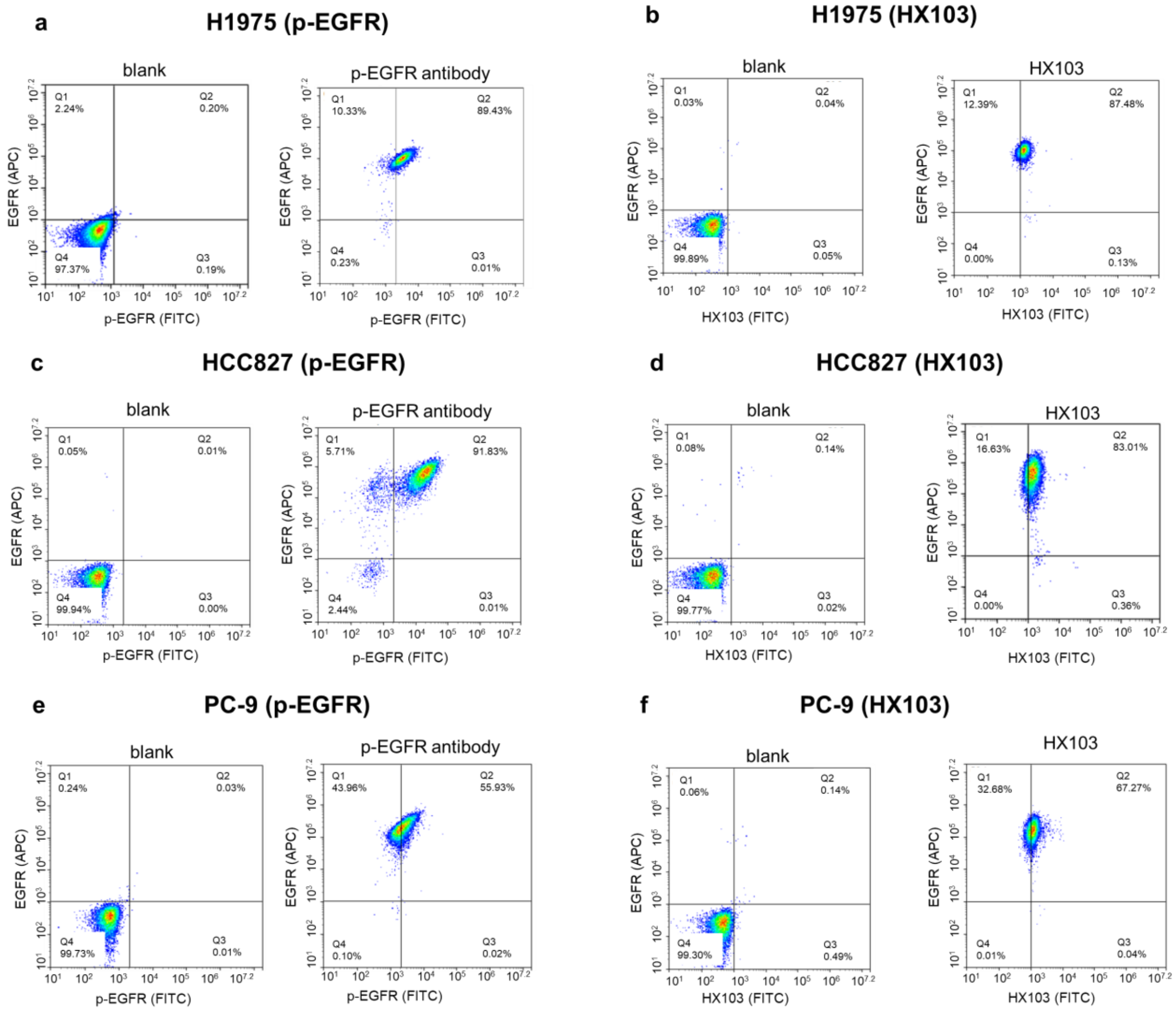

Supplementary Figure 9. Representative dot plots showing FACS analysis of the percent of phosphorated EGFR(+) cells (**a**, **c** and **e**) and HX103 (5 μM) labelled cells (**b**, **d** and **f**) in EGFR mutated cancer cells. Three biological independent experiments were repeated with similar results.

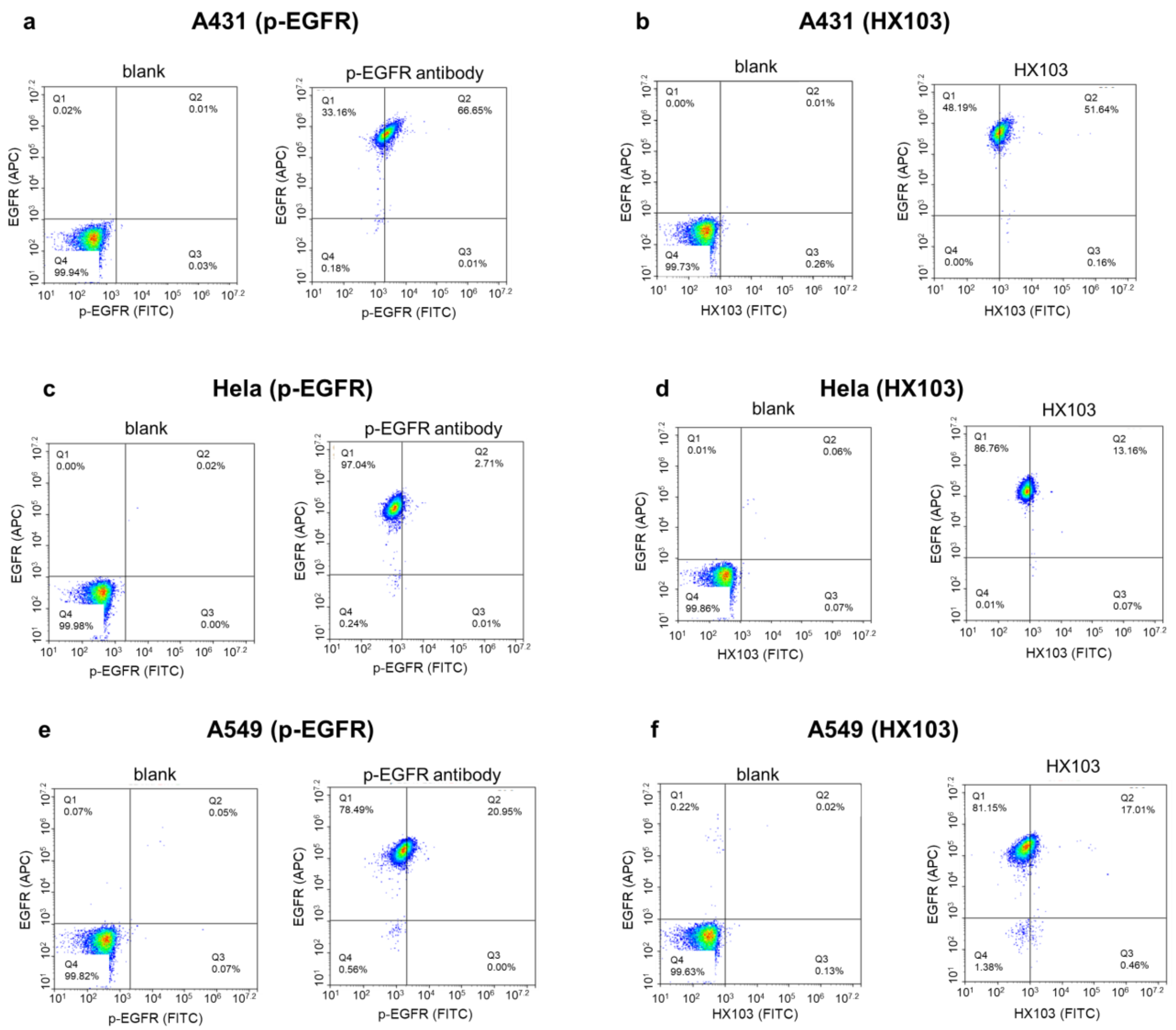

Supplementary Figure 10. Representative dot plots showing FACS analysis of the percent of phosphorated EGFR(+) cells (**a**, **c** and **e**) and HX103 (5 μM) labelled cells (**b**, **d** and **f**) in EGFR wild-type cancer cells. Three biological independent experiments were repeated with similar results.

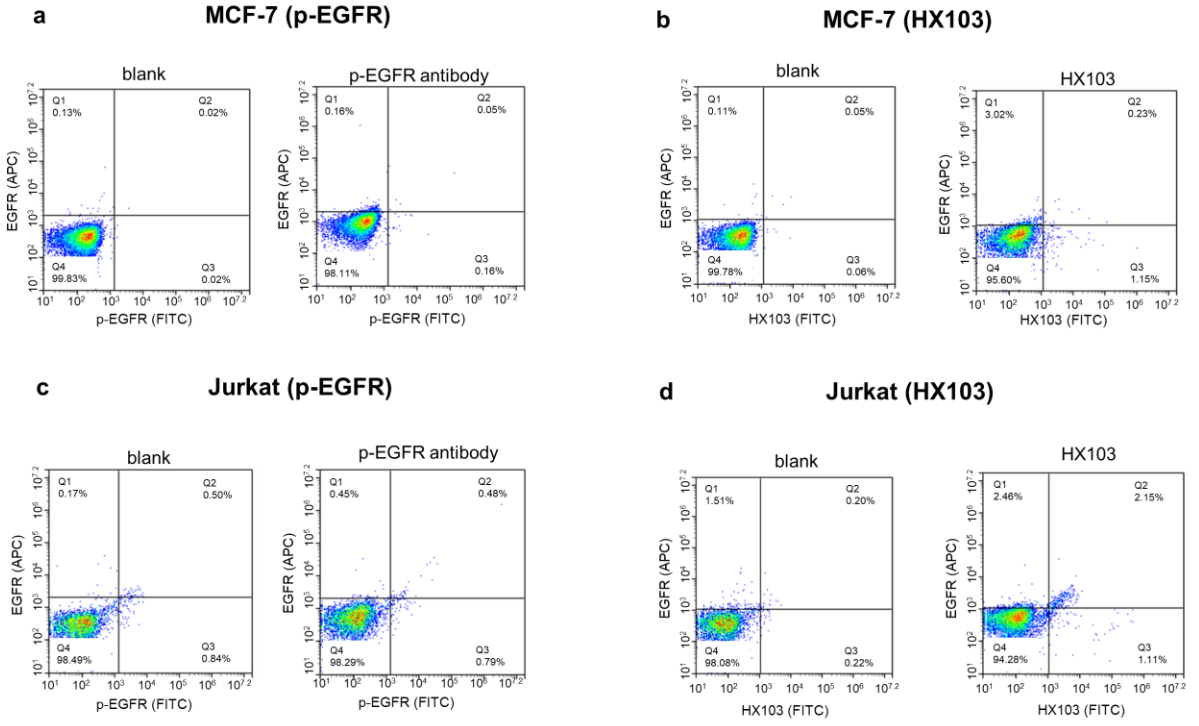

Supplementary Figure 11. Representative dot plots showing FACS analysis of the percent of phosphorated EGFR(+) cells (**a** and **c**) and HX103 (5 μM) labelled cells (**b** and **d**) in EGFR-negative cancer cells. Three biological independent experiments were repeated with similar results.

a

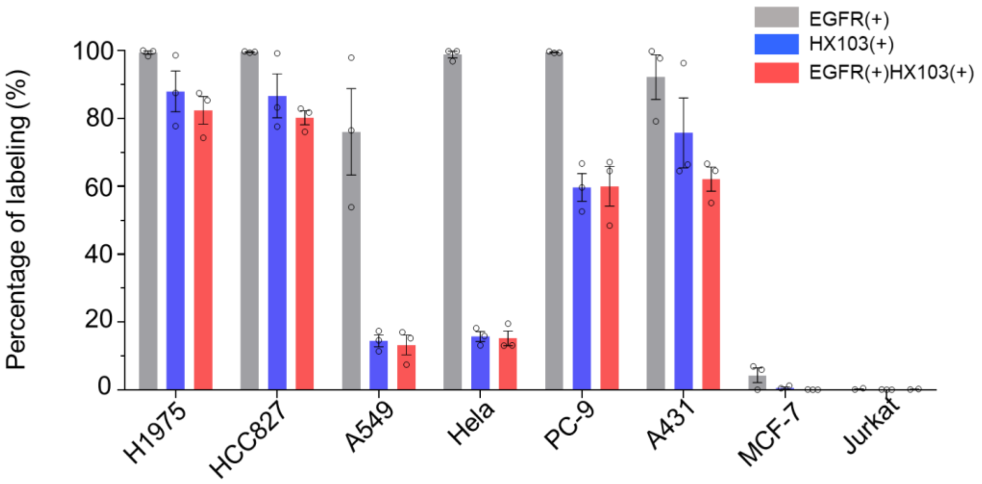

b

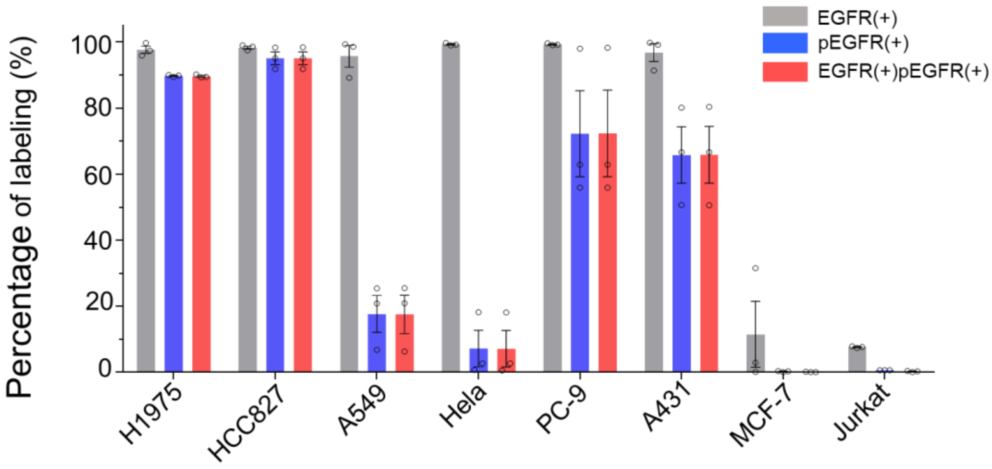

Supplementary Figure 12. Determination of the percent of HX103(+), EGFR(+), pEGFR(+), EGFR(+)HX103(+) and EGFR(+)pEGFR(+) in cells with flow cytometric experiments. (**a**) Bar chart showing the percentage values of HX103(+), EGFR(+) and EGFR(+)HX103(+) labeling in cancer cells with distinct forms of EGFR. Data represent average values ± SEM, n = 3 independent experiments per group. (**b**) Bar chart showing the percentage values of pEGFR(+), EGFR(+) and EGFR(+)pEGFR(+) labeling in cancer cells with distinct forms of EGFR. Data represent average values ± SEM, n = 3 independent experiments per group. Source data are provided as a Source Data file.

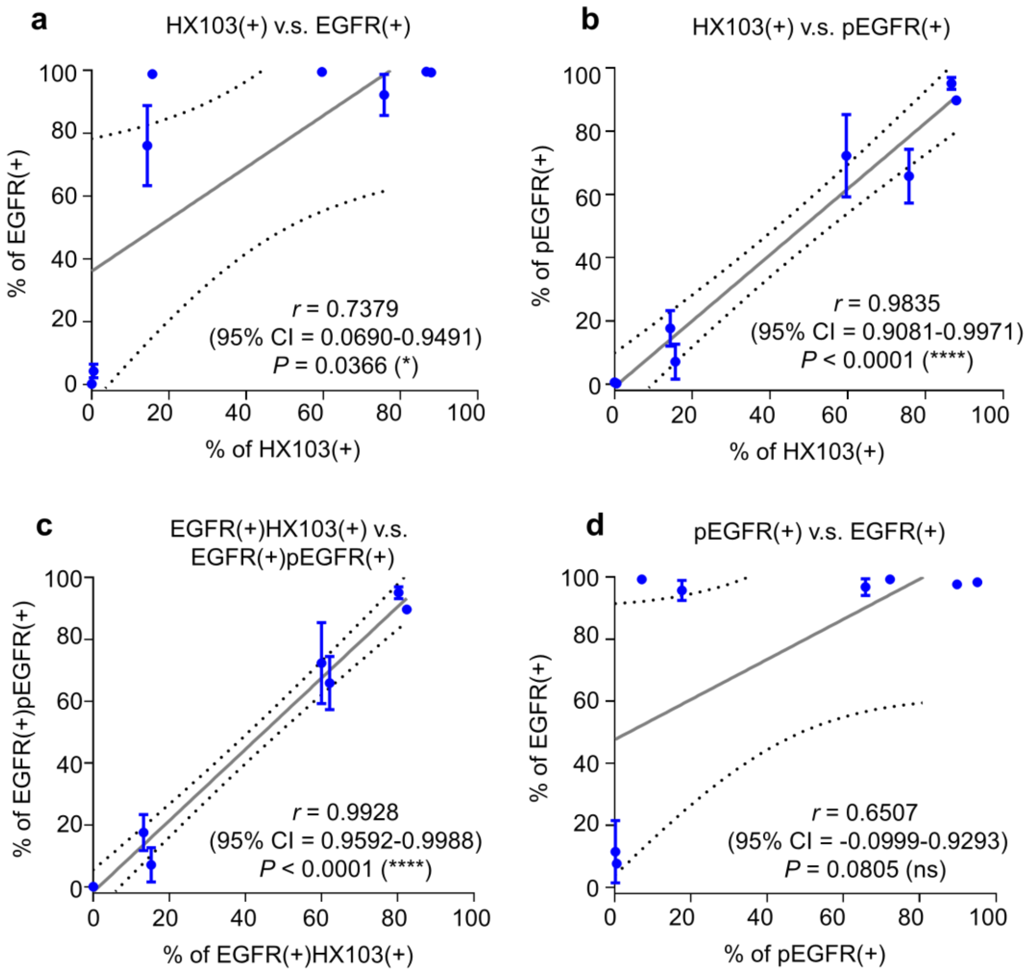

Supplementary Figure 13. Pearson's correlation analysis between HX103 labeling and EGFR expression\EGFR activation was performed in a panel of cancer cells (H1975, HCC827, A549, Hela, PC-9, A431, MCF-7 and Jurkat). (**a**) Correlation of the percentage values between HX103(+) and EGFR(+). Data represent average values ± SEM, n = 3 independent experiments per group. The statistical *P* values were calculated by the two-tailed Student's test, and *r* represents Pearson's correlation coefficient. (**b**) Correlation of the percentage values between HX103(+) and pEGFR(+). Data represent average values ± SEM, n = 3 independent experiments per group. The statistical *P* values were calculated by the two-tailed Student's test, and *r* represents Pearson's correlation coefficient. (**c**) Correlation of the percentage values between EGFR(+)HX103(+) and EGFR(+)pEGFR(+). Data represent average values ± SEM, n = 3 independent experiments per group. The statistical *P* values were calculated by the two-tailed Student's test, and *r* represents Pearson's correlation coefficient. (**d**) Correlation of the percentage values between pEGFR(+) and EGFR(+). Data represent average values ± SEM, n = 3 independent experiments per group. The statistical *P* values were calculated by the two-tailed Student's test, and *r* represents Pearson's correlation coefficient. Source data are provided as a Source Data file.

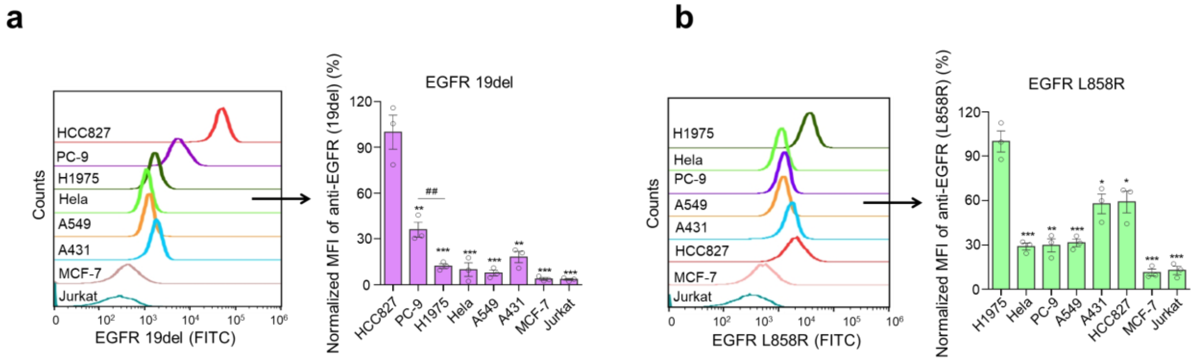

Supplementary Figure 14. Determination of the percent of EGFR mutant cells with flow cytometric experiments. (**a**) Flow cytometric experiments were performed to quantify the expression of EGFR 19del with EGFR 19del antibody. Data represent average values ± SEM, n = 3 independent experiments per group. The statistical *P* values were calculated by the two-tailed Student's test, $^{**}P<0.01$, $^{***}P<0.001$ for HCC827 cells compared with the rest cells; $^{\#\#}P<0.01$, for PC-9 cells compared with H1975 cells. HCC827 vs. PC-9, $P = 0.006$; HCC827 vs. A431, $P = 0.002$; PC-9 vs. H1975, $P = 0.009$. (**b**) Flow cytometric experiments were performed to quantify the expression of EGFR L858R with EGFR L858R antibody. Data represent average values ± SEM, n = 3 independent experiments per group. The statistical *P* values were calculated by the two-tailed Student's test, $^{*}P<0.05$, $^{**}P<0.01$, $^{***}P<0.001$ for H1975 cells compared with the rest cells. H1975 vs. PC-9, $P = 0.001$; H1975 vs. A431, $P = 0.013$; H1975 vs. HCC827, $P = 0.017$. Source data are provided as a Source Data file.

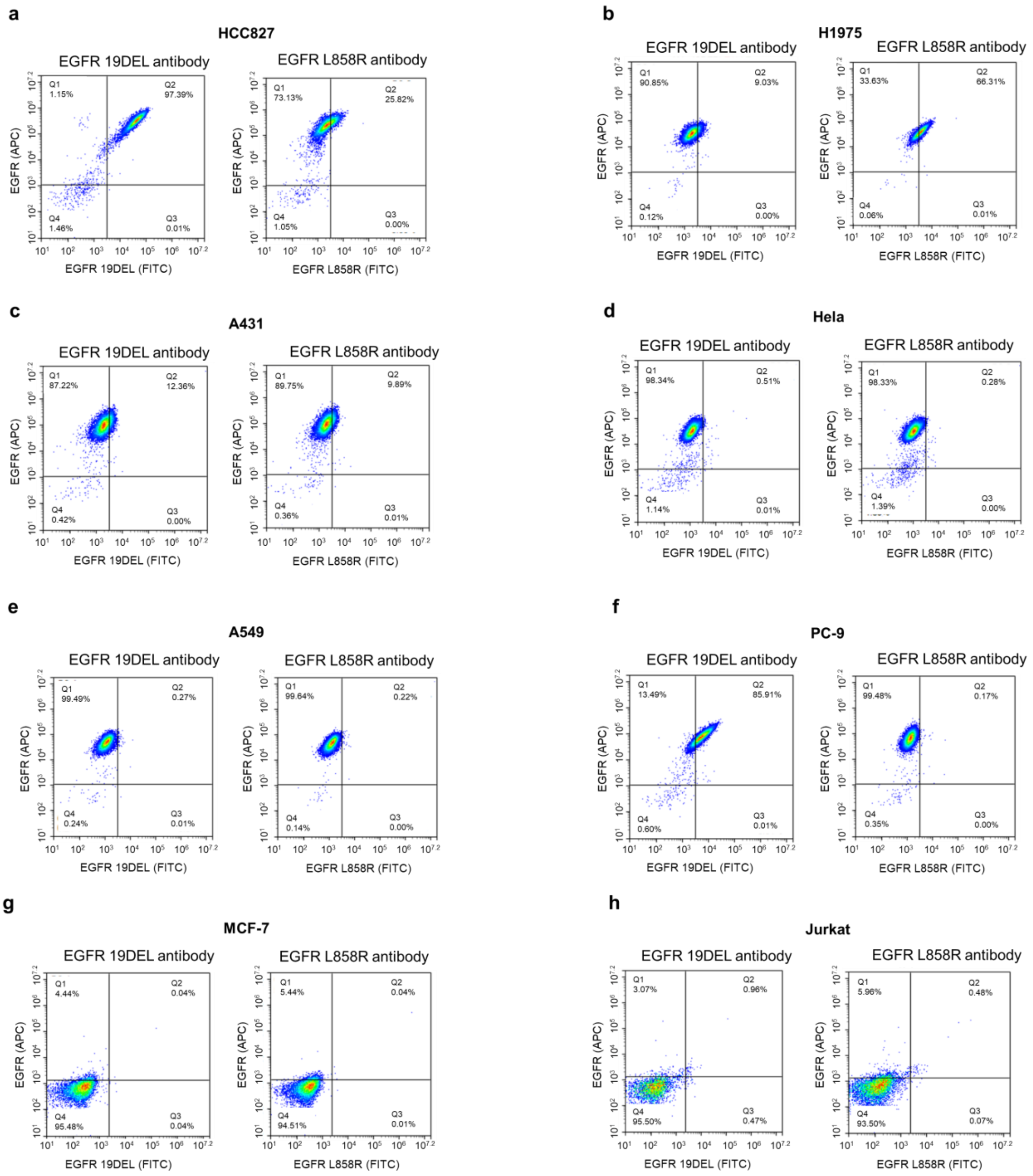

Supplementary Figure 15. Representative dot plots showing FACS analysis of the percentages of total EGFR that is mutant in various cells. Three biological independent experiments were repeated with similar results.

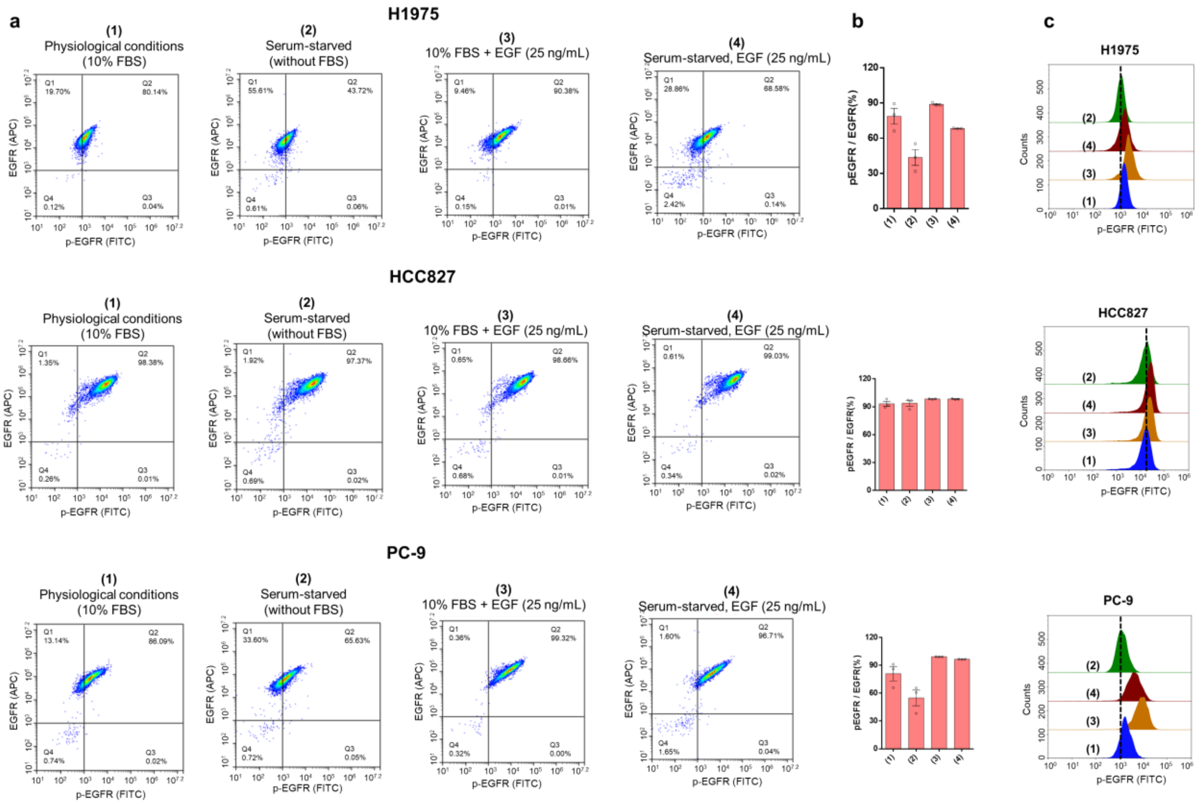

Supplementary Figure 16. Determination of the percent of EGFR(+)pEGFR(+) cells in the presence and absence of EGF or 10% FBS. (**a**) Representative dot plots showing FACS analysis of EGFR phosphorylation in *EGFR* mutation positive cancer cell lines (A431, A549 and Hela) among these conditions: (1) cells were cultured in the presence of 10% FBS; (2) cells were starved with the medium in the absence of FBS; (3) cells were cultured with 10% FBS and induced with EGF (25 ng/mL); and (4) Starved cells were induced with EGF (25 ng/mL). Three biological independent experiments were repeated with similar results. (**b**) Bar chart showing the differences of EGFR phosphorylation between the four conditions in (a). Data represent average values ± SEM, n = 3 independent experiments per group. (**c**) Representative histograms showing fluorescence intensity differences between the four conditions in (a). Three biological independent experiments were repeated with similar results.

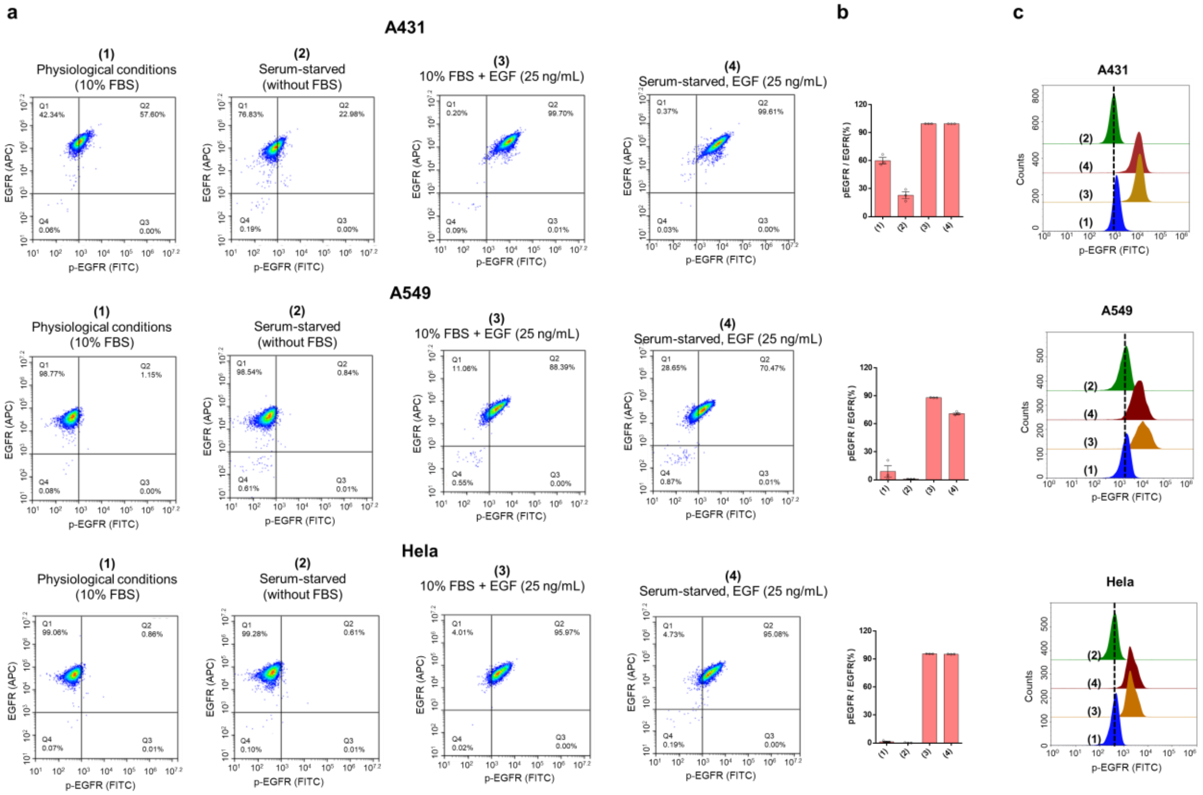

Supplementary Figure 17. Determination of the percent of EGFR(+)pEGFR(+) cells in the presence and absence of EGF or 10% FBS. (**a**) Representative dot plots showing FACS analysis of EGFR phosphorylation in *EGFR* wild-type cell lines (A431, A549 and Hela) among these conditions: (1) cells were cultured in the presence of 10% FBS; (2) cells were starved with the medium in the absence of FBS; (3) cells were cultured with 10% FBS and induced with EGF (25 ng/mL); and (4) Starved cells were induced with EGF (25 ng/mL). Three biological independent experiments were repeated with similar results. (**b**) Bar chart showing the differences of EGFR phosphorylation between the four conditions in (a). Data represent average values ± SEM, n = 3 independent experiments per group. (**c**) Representative histograms showing fluorescence intensity differences between the four conditions in (a). Three biological independent experiments were repeated with similar results.

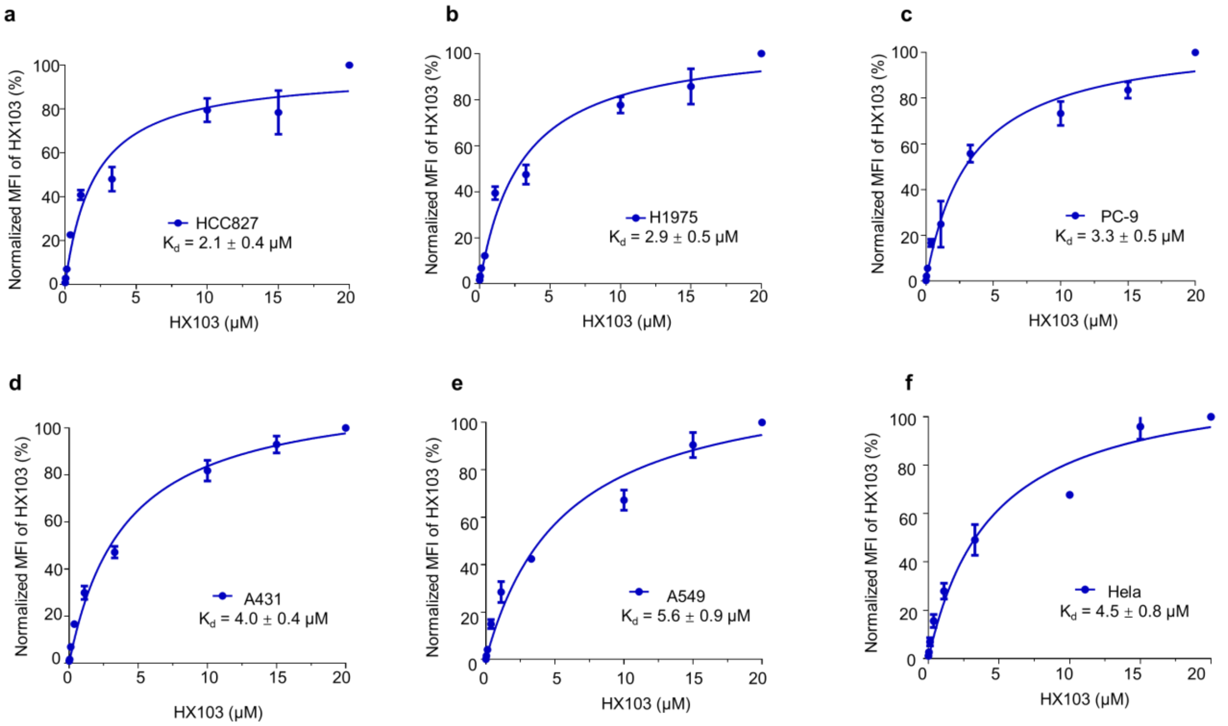

Supplementary Figure 18. The binding affinities of HX103 in various EGFR-positive cancer cells were determined with apparent K_d value of 2.1±0.4 μM (HCC827) (**a**), 2.9±0.5 μM (H1975) (**b**), 3.3±0.5 μM (PC-9) (**c**), 4.0±0.4 μM (A431) (**d**), 5.6±0.9 μM (A549) (**e**) and 4.5±0.8 μM (Hela) (**f**). Data represent average values ± SEM, n = 3 independent experiments per group. Source data are provided as a Source Data file.

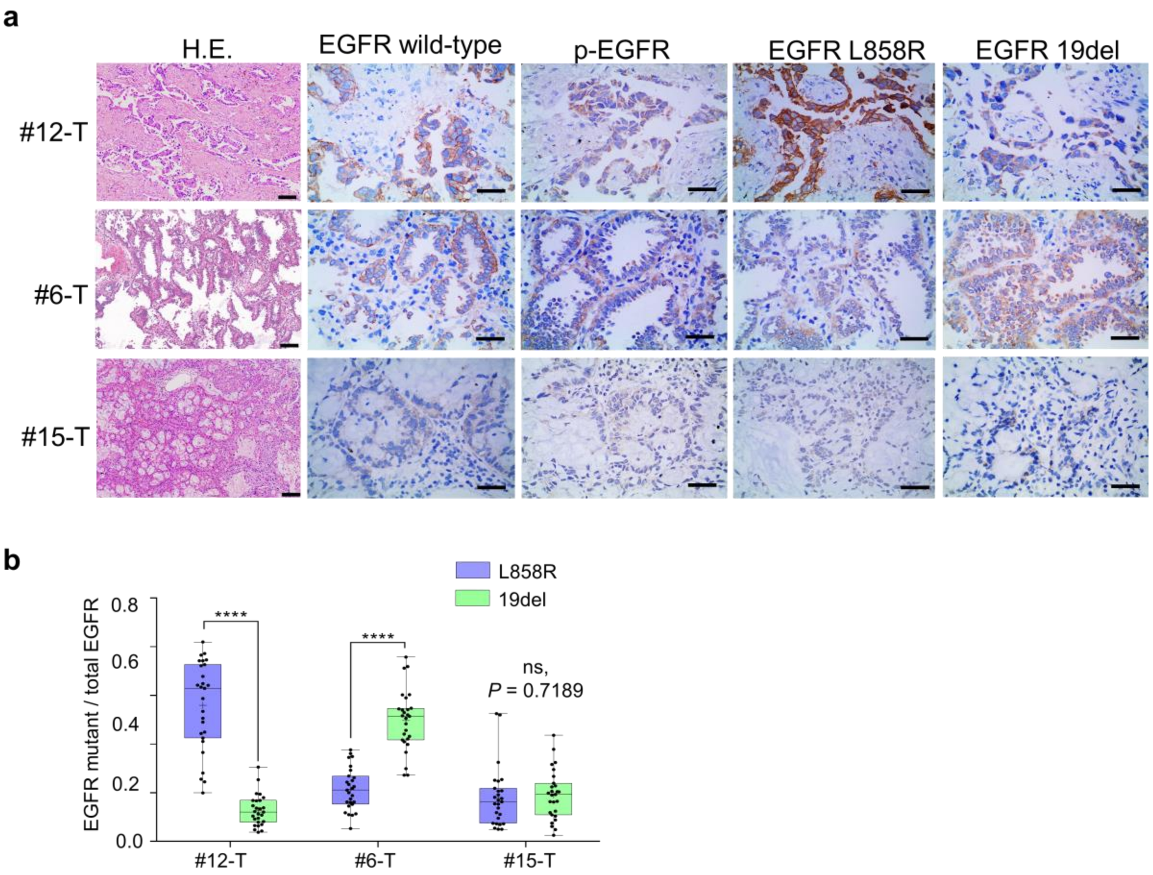

Supplementary Figure 19. Histological results of patients with *EGFR* mutations. (**a**) Representative histological confirmation of patients with *EGFR* mutations. H&E staining at 10x magnification. Scale bar, 100 µm. Immunohistochemistry for EGFR, p-EGFR, EGFR L858R and EGFR 19del at 20x magnification. Scale bar, 40 µm. Three biological independent experiments were repeated with similar results. (**b**) Box and whisker plots indicate the percent of mutant EGFR in total EGFR expression in tumor tissues. Data were quantified from IHC analysis (a). Average values are shown as "+", n = 27 per group (3 biological replicates × 9 ROIs per picture). The line in the box corresponds to the median. The boxes go from the upper to the lower quartiles of the data. Whiskers represent Min to Max shown with all data points. The statistical *P* values were calculated by the two-tailed Student's test, $^{****}P < 0.0001$, ns represents not significant (EGFR L858R vs. EGFR 19del, $P = 0.7189$). Data are provided as a Source Data file.

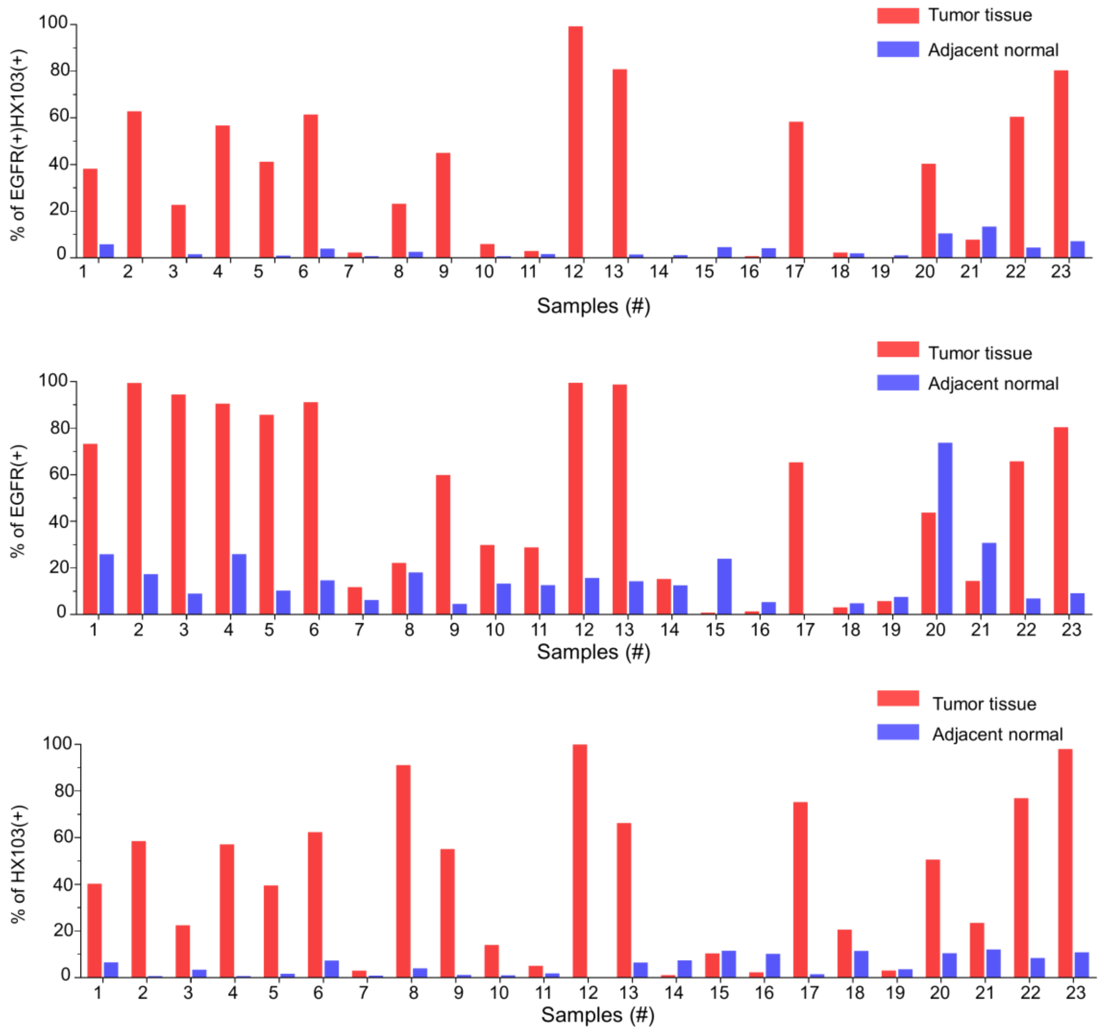

Supplementary Figure 20. The bar chart showing the percentage values of EGFR(+)HX103(+), EGFR(+) and HX103(+) in surgical samples (tumor and the adjacent normal) from patients with NSCLC (Supplementary Table 5). Source data are provided as a Source Data file.

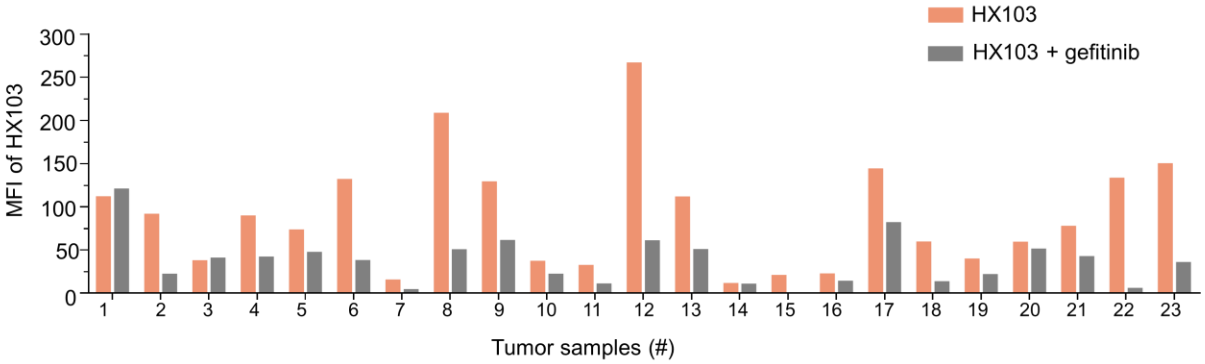

Supplementary Figure 21. The bar chart showing the mean fluorescence intensity (MFI) of HX103 in 23 surgical tumor samples (Supplementary Table 6). Source data are provided as a Source Data file.

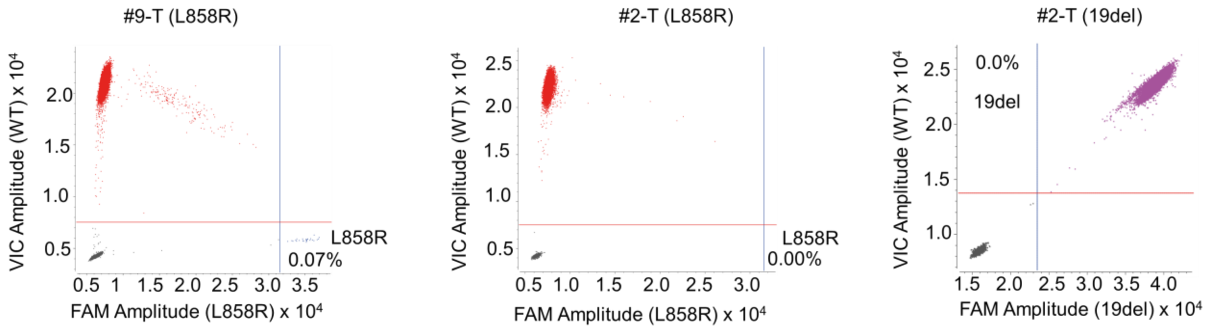

Supplementary Figure 22. Two-dimensional scatterplots of droplet digital PCR *EGFR* assay showing *EGFR* L858R negative for tumor samples #2-T and #9-T, and EGFR 19del negative for tumor sample #2-T. Source data are provided as a Source Data file.

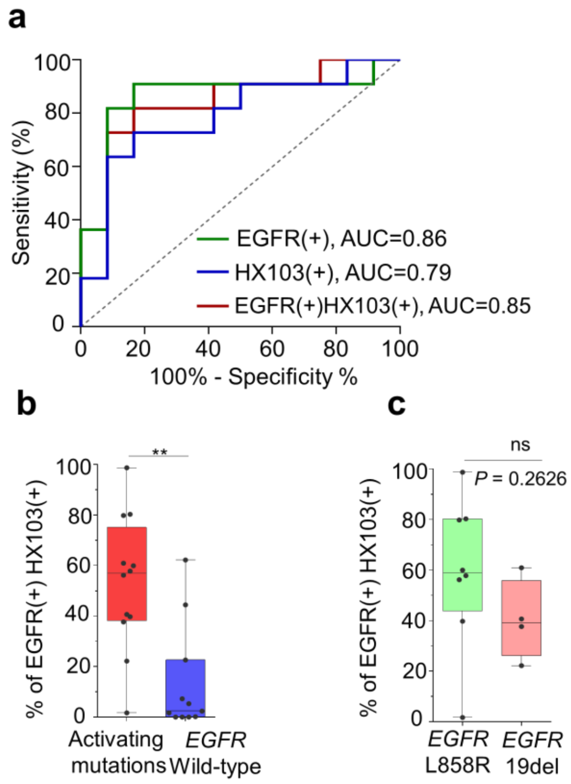

Supplementary Figure 23. Correlations between HX103-based FACS and *EGFR*-activating mutations in surgically resected samples from NSCLC patients. (**a**) Receiver operating characteristic (ROC) curve representing the sensitivity and specificity of HX103(+), EGFR(+) and EGFR(+)HX103(+) for predicting the presence of *EGFR* mutations in surgical tumor samples [HX103(+): AUC = 0.79; EGFR(+): AUC = 0.86; EGFR(+)HX103(+): AUC = 0.85]. (**b**) Box and whisker plot of EGFR(+)HX103(+) in tumor samples with *EGFR*-activating mutations and wild-type. Average values are shown as "+". The line in the box corresponds to the median. The boxes go from the upper to the lower quartiles of the data. Whiskers represent Min to Max shown with all data points. For *EGFR*-activating mutation group, n = 12; for *EGFR* wild-type group, n = 11. Statistics was performed using Mann-Whitney test ($^{**}P = 0.004 < 0.01$, two-sided). (**c**) Box and whisker plot of EGFR(+)HX103(+) in tumor samples with *EGFR* L858R and 19del. Average values are shown as "+". The line in the box corresponds to the median. The boxes go from the upper to the lower quartiles of the data. Whiskers represent Min to Max shown with all data points. For the group of *EGFR* L858R, n = 8; for the group of *EGFR* 19del, n = 4. Statistics was performed using Mann-Whitney test ($P = 0.2626$, ns represents not significant, two-sided). Source data are provided as a Source Data file.

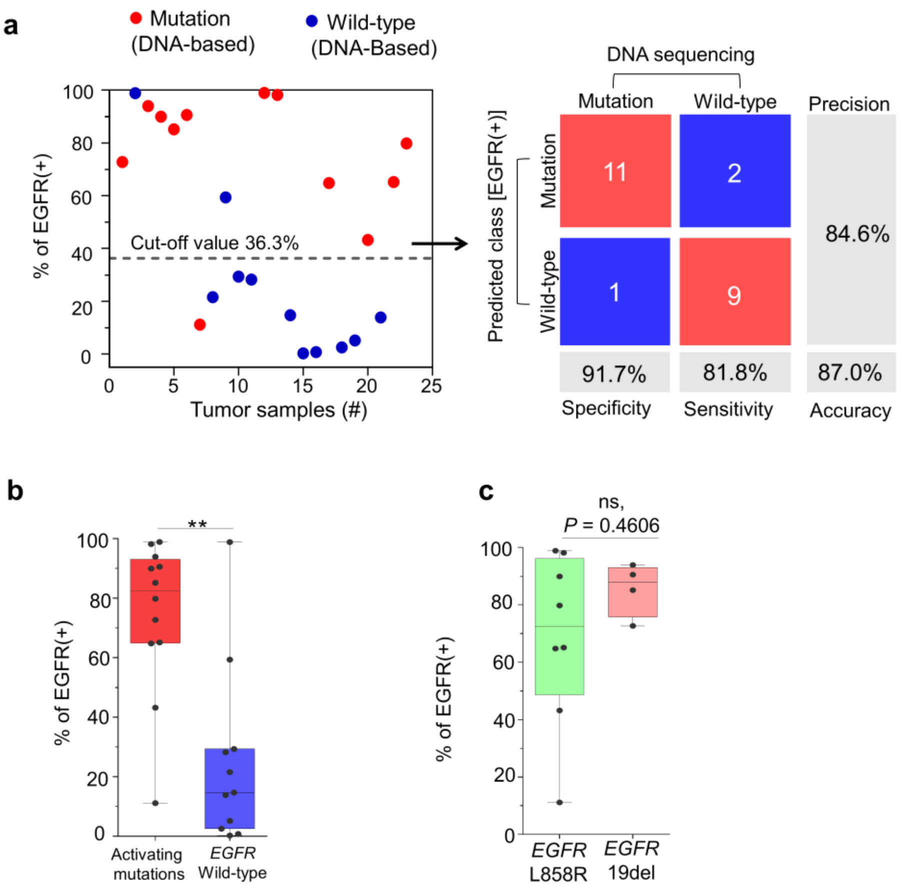

Supplementary Figure 24. Analysis of the tumor tissues from NSCLC patients with the percent of EGFR(+) determined by HX103-based FACS. (**a**) Results of 23 surgical tumor samples from NSCLC patients predicted by the percentage values of EGFR(+). (**b**) Box and whisker plot of EGFR(+) in tumor samples with *EGFR*-activating mutations and wild-type. Average values are shown as “+”. The line in the box corresponds to the median. The boxes go from the upper to the lower quartiles of the data. Whiskers represent Min to Max shown with all data points. For *EGFR*-activating mutation group, n = 12; for *EGFR* wild-type group, n = 11. Statistics was performed using Mann-Whitney test ($^{**}P = 0.002 < 0.01$, two-sided). (**c**) Box and whisker plot of EGFR(+)in surgical tumor samples with *EGFR* 19del and L858R. The mean % values of EGFR(+) were 68.9±10.6% and 85.6±4.7% for *EGFR* L858R and 19del, respectively. No statistical association was found between EGFR(+) and *EGFR* 19del or L858R mutation (*P* = 0.4606, ns represents not significant, two-sided). Average values are shown as “+”. The line in the box corresponds to the median. The boxes go from the upper to the lower quartiles of the data. Whiskers represent Min to Max shown with all data points. For the group of *EGFR* L858R, n = 8; for the group of *EGFR* 19del, n = 4. Statistics was performed using Mann-Whitney test. Source data are provided as a Source Data file.

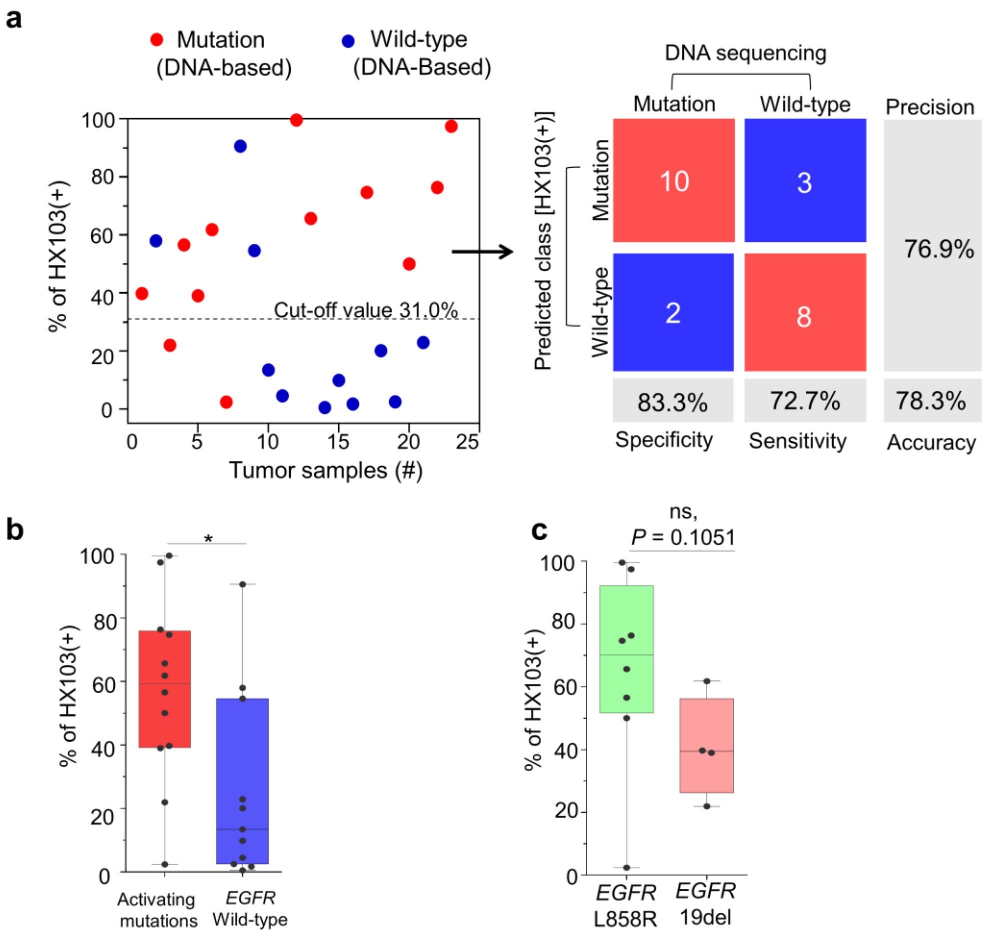

Supplementary Figure 25. Analysis of the tumor tissues from NSCLC patients with the percent of HX103(+) determined by HX103-based FACS. (**a**) Results of 23 surgical tumor samples from NSCLC patients predicted by the percentage values of HX103(+). (**b**) Box and whisker plot of HX103(+) in tumor samples with *EGFR*-activating mutations and wild-type. Average values are shown as "+". The line in the box corresponds to the median. The boxes go from the upper to the lower quartiles of the data. Whiskers represent Min to Max shown with all data points. For *EGFR*-activating mutation group, n = 12; for *EGFR* wild-type group, n = 11. Statistics was performed using Mann-Whitney test ($^{*}P = 0.019 < 0.05$, two-sided). (**c**) Box and whisker plot of HX103(+) in surgical tumor samples with *EGFR* 19del and L858R. The mean % values of HX103(+) were 65.3±10.9% and 40.6±8.2% for *EGFR* L858R and 19del, respectively. Average values are shown as "+". The line in the box corresponds to the median. The boxes go from the upper to the lower quartiles of the data. Whiskers represent Min to Max shown with all data points. For the group of *EGFR* L858R, n = 8; for the group of *EGFR* 19del, n = 4. No statistical association was found between HX103(+) and *EGFR* 19del or L858R mutation ($P = 0.1051$, ns represents not significant, two-sided). Statistics was performed using Mann-Whitney test. Source data are provided as a Source Data file.

#1-T (*EGFR* 19del)

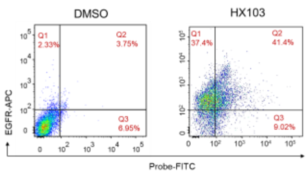

	Q1+Q2 EGFR(+) %	Q2+Q3 HX103(+) %	Q2 EGFR(+)HX103(+) %
DMSO	6.08	10.7	3.75
HX103	78.8	50.42	41.4
Corrected	72.72	39.72	37.65

#2-T (No *EGFR*-activating mutations)

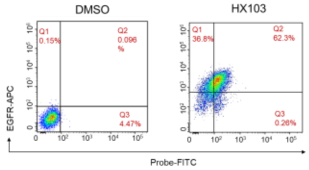

	Q1+Q2 EGFR(+)%	Q2+Q3 HX103(+)%	Q2 EGFR(+)HX103(+) %
DMSO	0.246	4.566	0.096
HX103	99.1	62.56	62.3
Corrected	98.854	57.994	62.204

#3-T (*EGFR* 19del)

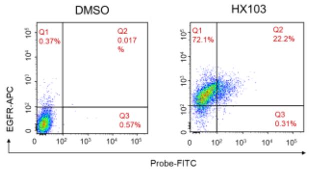

	Q1+Q2 EGFR(+)%	Q2+Q3 HX103(+)%	Q2 EGFR(+)HX103(+) %
DMSO	0.387	0.587	0.017
HX103	94.3	22.51	22.2
Corrected	93.913	21.923	22.183

#4-T (*EGFR* L858R)

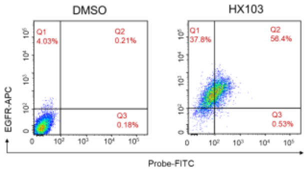

	Q1+Q2 EGFR(+)%	Q2+Q3 HX103(+)%	Q2 EGFR(+)HX103(+) %
DMSO	4.24	0.39	0.21
HX103	94.2	56.93	56.4
Corrected	89.96	56.54	56.19

#5-T (*EGFR* 19del)

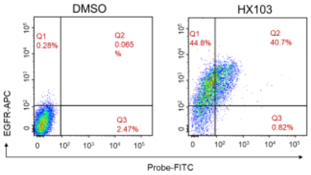

	Q1+Q2 EGFR(+) %	Q2+Q3 HX103(+) %	Q2 EGFR(+)HX103(+) %
DMSO	0.345	2.535	0.065
HX103	85.5	41.52	40.7
Corrected	85.155	38.985	40.635

#6-T (*EGFR* 19del)

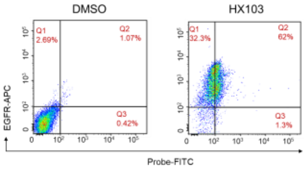

	Q1+Q2 EGFR(+)%	Q2+Q3 HX103(+) %	Q2 EGFR(+)HX103(+) %
DMSO	3.76	1.49	1.07
HX103	94.3	63.3	62
Corrected	90.54	61.81	60.93

#7 -T (*EGFR* L858R)

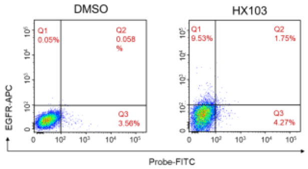

	Q1+Q2 EGFR(+) %	Q2+Q3 HX103(+) %	Q2 EGFR(+)HX103(+) %
DMSO	0.108	3.618	0.058
HX103	11.28	6.02	1.75
Corrected	11.172	2.402	1.692

#8-T (No *EGFR*-activating mutations)

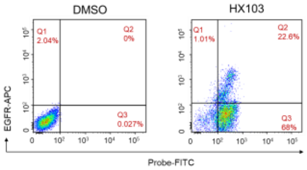

	Q1+Q2 EGFR(+) %	Q2+Q3 HX103(+) %	Q2 EGFR(+)HX103(+) %
DMSO	2.04	0.027	0
HX103	23.61	90.6	22.6
Corrected	21.57	90.573	22.6

#9-T (No *EGFR*-activating mutations)

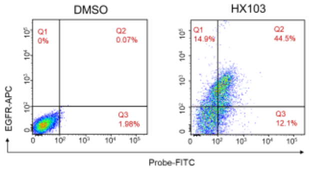

	Q1+Q2 EGFR(+) %	Q2+Q3 HX103(+) %	Q2 EGFR(+)HX103(+) %
DMSO	0.07	2.05	0.07
HX103	59.4	56.6	44.5
Corrected	59.33	54.55	44.43

#10-T (No *EGFR*-activating mutations)

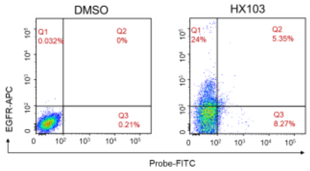

	Q1+Q2 EGFR(+) %	Q2+Q3 HX103(+) %	Q2 EGFR(+)HX103(+) %
DMSO	0.032	0.21	0
HX103	29.35	13.62	5.35
Corrected	29.318	13.41	5.35

#11-T (No *EGFR*-activating mutations)

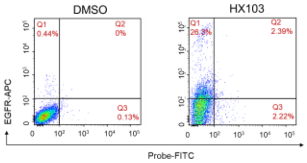

	Q1+Q2 EGFR(+) %	Q2+Q3 HX103(+) %	Q2 EGFR(+)HX103(+) %
DMSO	0.44	0.13	0
HX103	28.69	4.61	2.39
Corrected	28.25	4.48	2.39

#12-T (*EGFR* L858R)

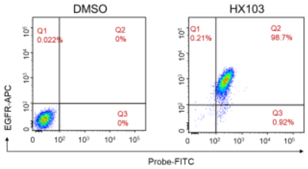

	Q1+Q2 EGFR(+) %	Q2+Q3 HX103(+) %	Q2 EGFR(+)HX103(+) %
DMSO	0.022	0	0
HX103	98.91	99.62	98.7
Corrected	98.888	99.62	98.7

Supplementary Figure 26. The summary of dual-parameter dot plots (HX103/EGFR antibody) determined by HX103-based FACS in surgical tumor tissues (from #1-T to #12-T). The calculations of percentage values of EGFR(+), HX103(+) and EGFR(+)HX103(+) were showed in the tables.

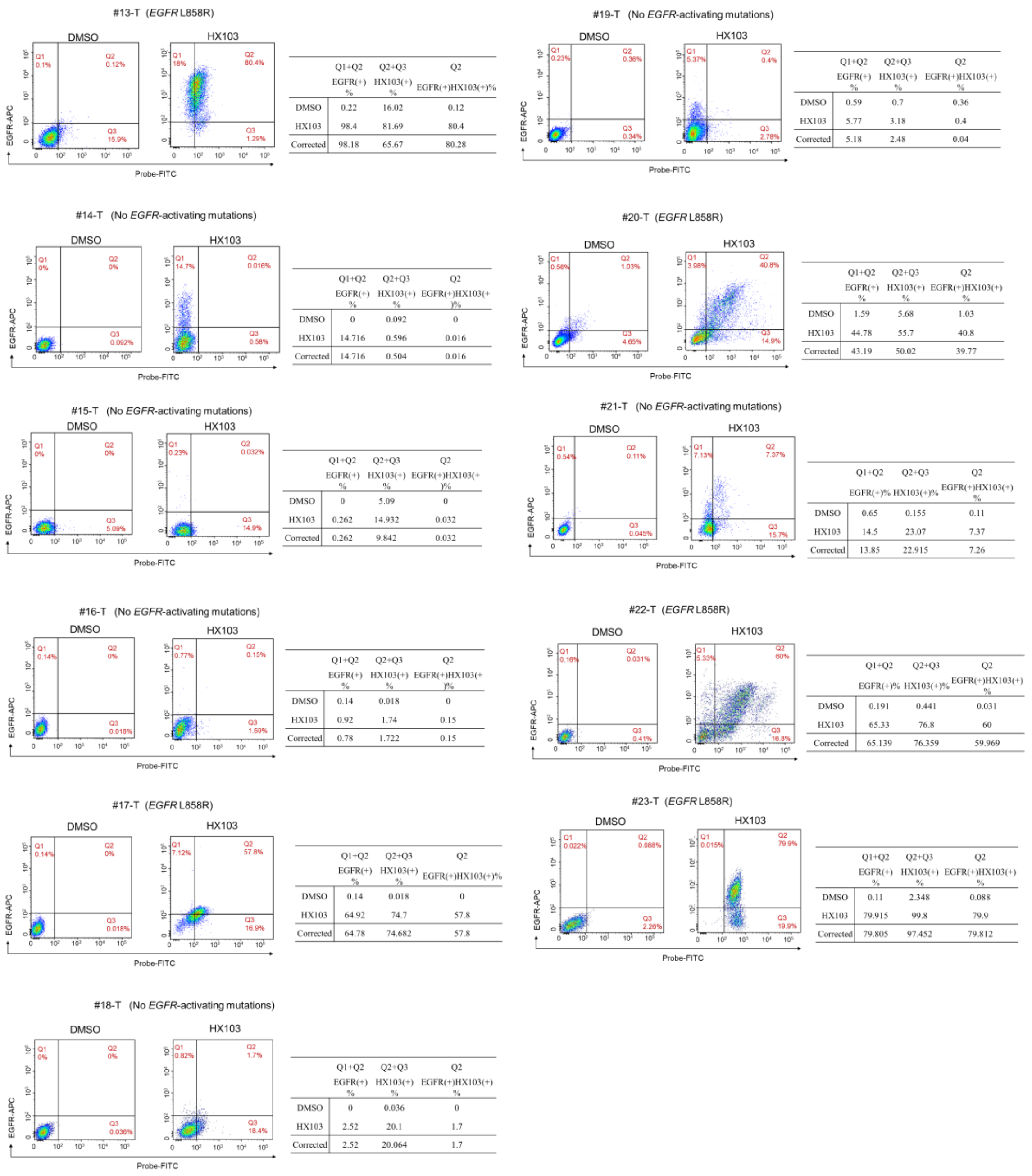

#13-T (*EGFR* L858R)

	Q1+Q2 EGFR(+)%	Q2+Q3 HX103(+)%	Q2 EGFR(+)HX103(+)%
DMSO	0.22	16.02	0.12
HX103	98.4	81.69	80.4
Corrected	98.18	65.67	80.28

#19-T (No *EGFR*-activating mutations)

	Q1+Q2 EGFR(+)%	Q2+Q3 HX103(+)%	Q2 EGFR(+)HX103(+)%
DMSO	0.59	0.7	0.36
HX103	5.77	3.18	0.4
Corrected	5.18	2.48	0.04

#14-T (No *EGFR*-activating mutations)

	Q1+Q2 EGFR(+)%	Q2+Q3 HX103(+)%	Q2 EGFR(+)HX103(+)%
DMSO	0	0.092	0
HX103	14.716	0.596	0.016
Corrected	14.716	0.504	0.016

#20-T (*EGFR* L858R)

	Q1+Q2 EGFR(+)%	Q2+Q3 HX103(+)%	Q2 EGFR(+)HX103(+)%
DMSO	1.59	5.68	1.03
HX103	44.78	55.7	40.8
Corrected	43.19	50.02	39.77

#15-T (No *EGFR*-activating mutations)

	Q1+Q2 EGFR(+)%	Q2+Q3 HX103(+)%	Q2 EGFR(+)HX103(+)%
DMSO	0	5.09	0
HX103	0.262	14.932	0.032
Corrected	0.262	9.842	0.032

#21-T (No *EGFR*-activating mutations)

	Q1+Q2 EGFR(+)%	Q2+Q3 HX103(+)%	Q2 EGFR(+)HX103(+)%
DMSO	0.65	0.155	0.11
HX103	14.5	23.07	7.37
Corrected	13.85	22.915	7.26

#16-T (No *EGFR*-activating mutations)

	Q1+Q2 EGFR(+)%	Q2+Q3 HX103(+)%	Q2 EGFR(+)HX103(+)%
DMSO	0.14	0.018	0
HX103	0.92	1.74	0.15
Corrected	0.78	1.722	0.15

#22-T (*EGFR* L858R)

	Q1+Q2 EGFR(+)%	Q2+Q3 HX103(+)%	Q2 EGFR(+)HX103(+)%
DMSO	0.191	0.441	0.031
HX103	65.33	76.8	60
Corrected	65.139	76.359	59.969

#17-T (*EGFR* L858R)

	Q1+Q2 EGFR(+)%	Q2+Q3 HX103(+)%	Q2 EGFR(+)HX103(+)%
DMSO	0.14	0.018	0
HX103	64.92	74.7	57.8
Corrected	64.78	74.682	57.8

#23-T (*EGFR* L858R)

	Q1+Q2 EGFR(+)%	Q2+Q3 HX103(+)%	Q2 EGFR(+)HX103(+)%
DMSO	0.11	2.348	0.088
HX103	79.915	99.8	79.9
Corrected	79.805	97.452	79.812

#18-T (No *EGFR*-activating mutations)

	Q1+Q2 EGFR(+)%	Q2+Q3 HX103(+)%	Q2 EGFR(+)HX103(+)%
DMSO	0	0.036	0
HX103	2.52	20.1	1.7
Corrected	2.52	20.064	1.7

Supplementary Figure 27. The summary of dual-parameter dot plots (HX103/EGFR antibody) determined by HX103-based FACS in surgical tumor tissues (from #13-T to #23-T). The calculations of percentage values of EGFR(+), HX103(+) and EGFR(+)HX103(+) were showed in the tables.

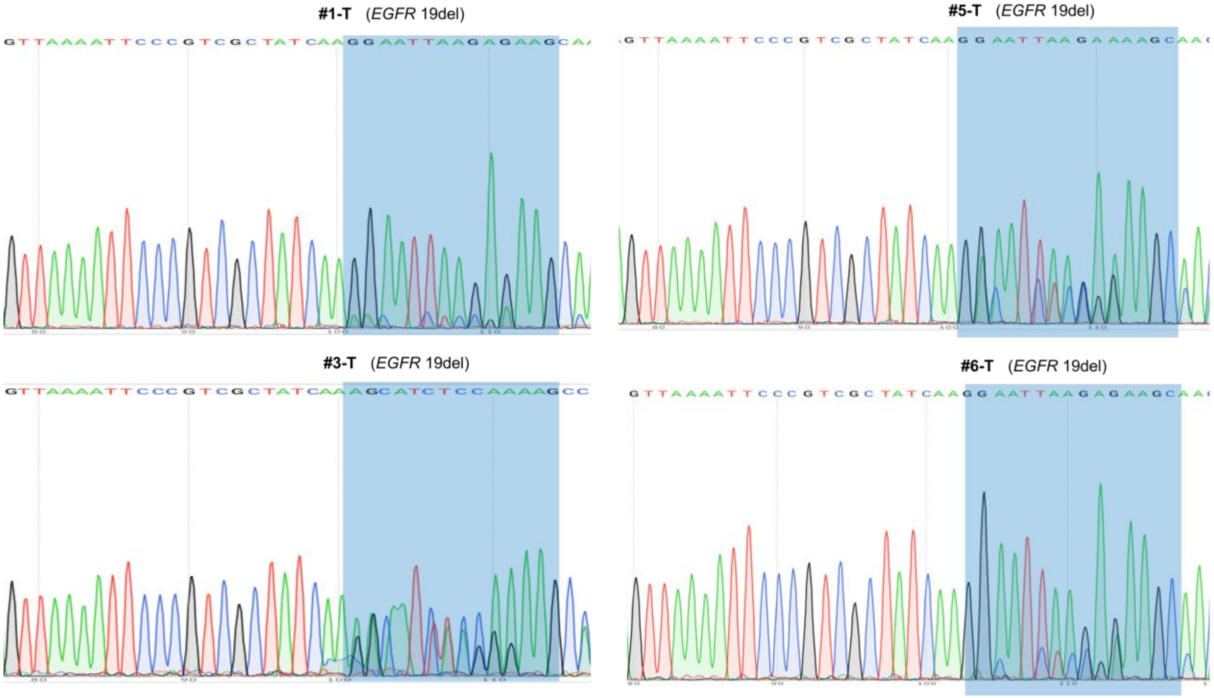

Supplementary Figure 28. The summary of gene sequencing confirmation for surgical tumor samples with *EGFR* 19del (#1-T, #3-T, #5-T and #6-T). Start of the exon 19 (GGAATTAAGAGAAGC) deletion is indicated by the blue area.

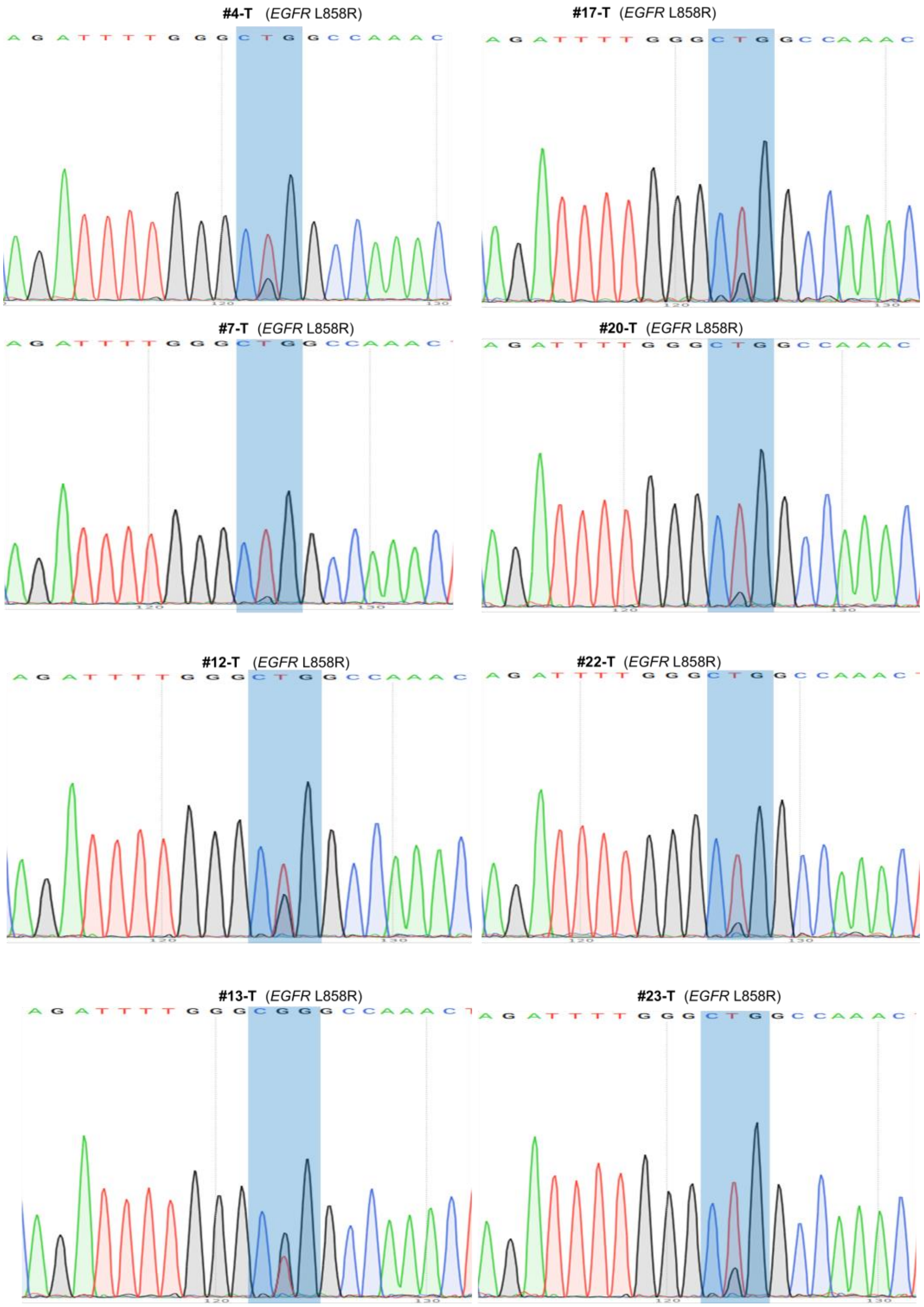

Supplementary Figure 29. The summary of gene sequencing confirmation for surgical tumor samples with *EGFR* L858R (#4-T, #7-T, #12-T, #13-T, #17-T, #20-T, #22-T and #23-T).

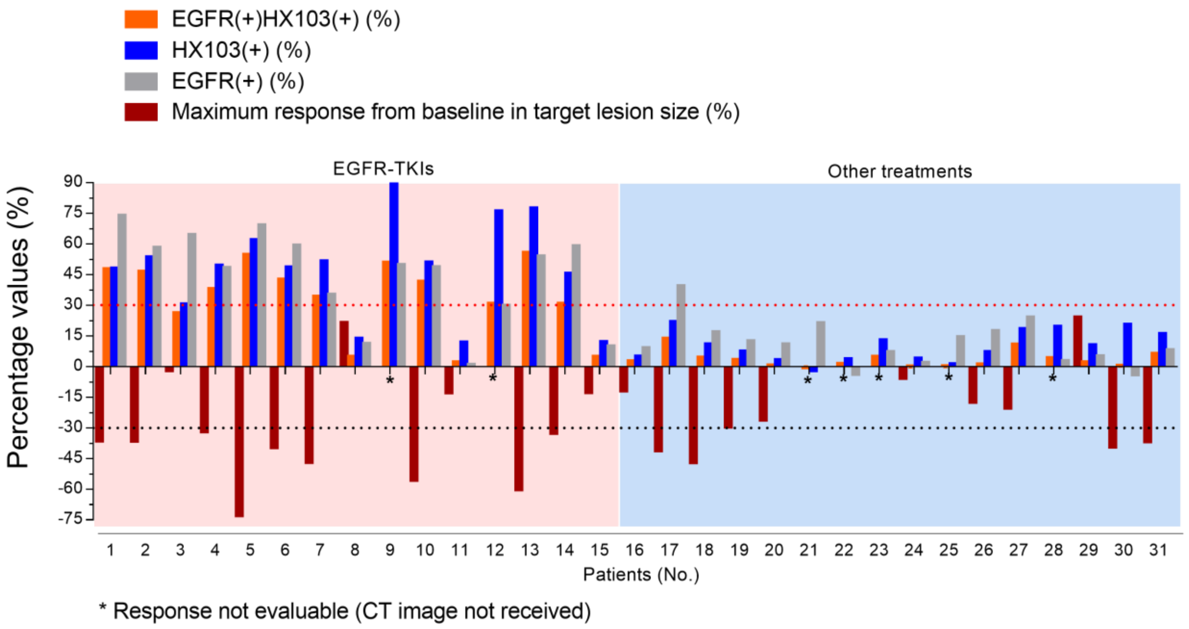

Supplementary Figure 30. Bar charts showing the best percentage change in target lesion size (red), the percentage values of EGFR(+) (grey), HX103(+) (blue) and EGFR(+)HX103(+) (orange) for NSCLC patients (*n* = 31). Of note, patients 1-15 received EGFR-TKIs, whereas patients 16-31 were treated with other methods. Source data are provided as a Source Data file.

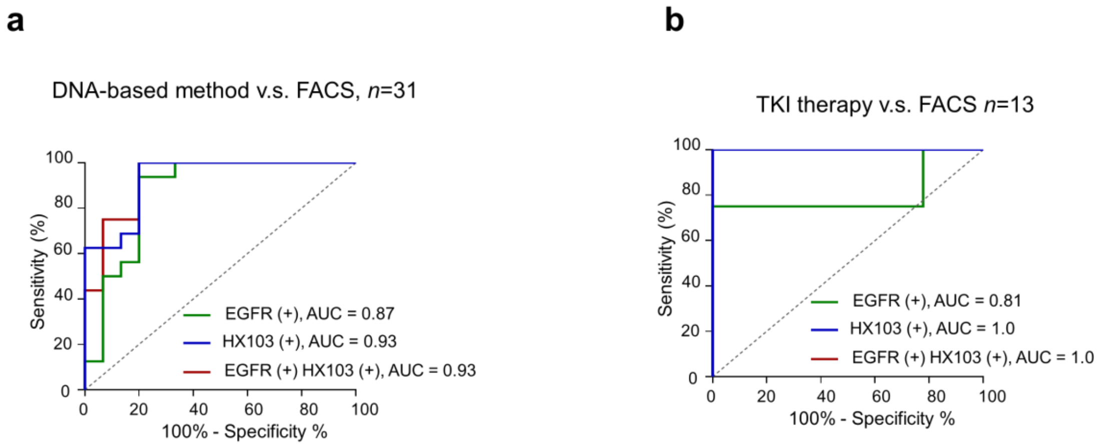

Supplementary Figure 31. Summary of the ROC analysis for biopsy samples from NSCLC patients. (**a**) ROC analysis for HX103(+), EGFR(+) and EGFR(+)HX103(+) for predicting *EGFR*-activating mutations (DNA-based method) in biopsy samples [HX103(+): AUC = 0.93; EGFR(+):AUC = 0.87; EGFR(+)HX103(+):AUC = 0.93]. (**b**) ROC analysis for HX103(+), EGFR(+) and EGFR(+)HX103(+) for predicting EGFR-TKI sensitivity (PR and CR were considered as objective response) in biopsy samples [HX103(+):AUC = 1.0; EGFR(+):AUC = 0.81; EGFR(+)HX103(+):AUC = 1.0]. Source data are provided as a Source Data file.

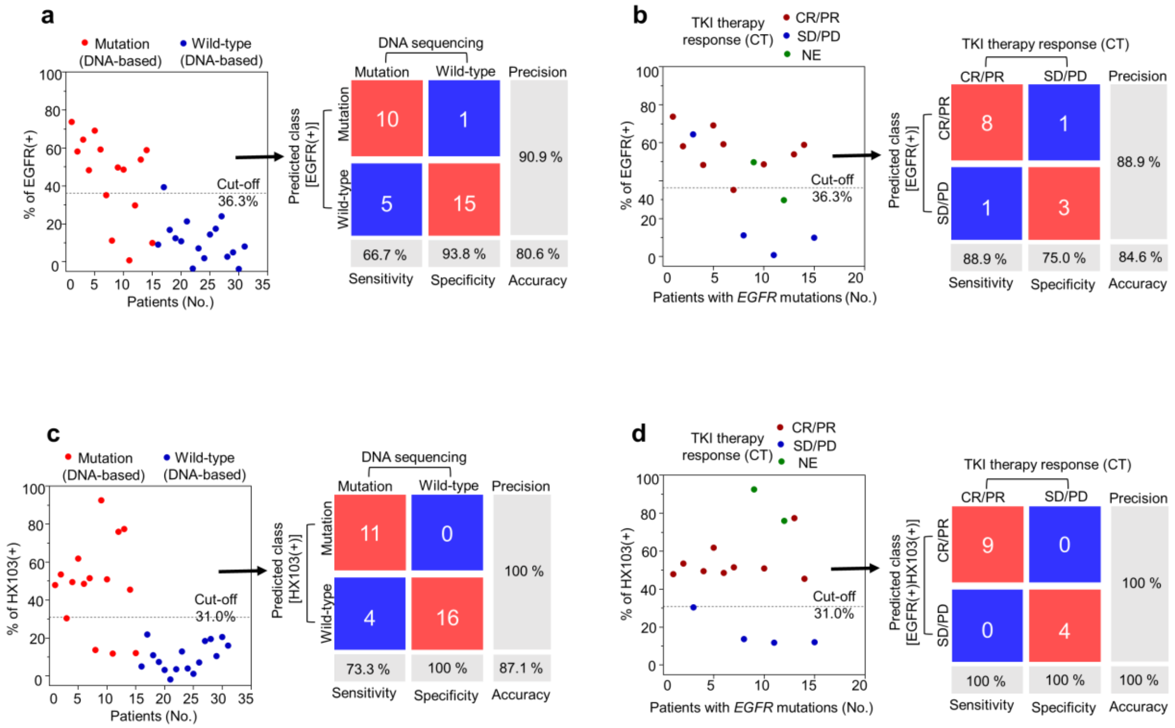

Supplementary Figure 32. Analysis of the biopsy samples from NSCLC patients with the percent of EGFR(+) or HX103(+) determined by HX103-based FACS. (**a**) Comparing the percentage values of EGFR(+) and *EGFR*-activating mutations (DNA-based method) in 31 biopsy samples from NSCLC patients to study the correlations between EGFR(+) labeling and *EGFR*-activating mutations. (**b**) Comparing the percentage values of EGFR(+) and EGFR-TKI therapy response (determined by CT imagines) in 15 NSCLC patients to study the correlations between EGFR(+) labeling and EGFR-TKI therapy response. Of note, therapy responses of 2 patients (9 and 12) were not evaluated. (**c**) Comparing the percentage values of HX103(+) and *EGFR*-activating mutations (DNA-based method) in 31 biopsy samples from NSCLC patients to study the correlations between HX103(+) labeling and *EGFR*-activating mutations. (**d**) Comparing the percentage values of HX103(+) and EGFR-TKI therapy response (determined by CT imagines) in 15 NSCLC patients to study the correlations between HX103(+) labeling and EGFR-TKI therapy response. Of note, therapy responses of 2 patients (9 and 12) were not evaluated.

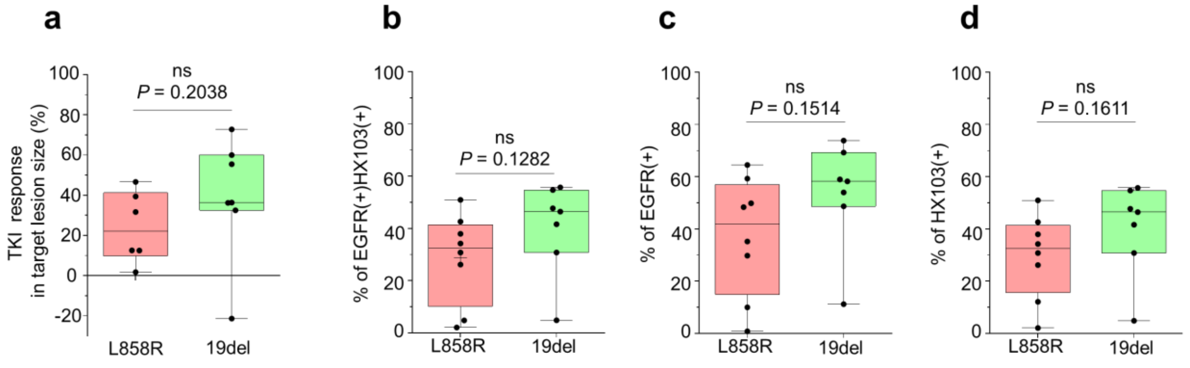

Supplementary Figure 33. The associations between EGFR mutation status and TKI response or HX103-based FACS analysis. (**a**) Box and whisker plots indicate the associations between EGFR-TKI response and *EGFR* L858R or 19del mutation status. Average values are shown as "+". The line in the box corresponds to the median. The boxes go from the upper to the lower quartiles of the data. Whiskers represent Min to Max shown with all data points. For *EGFR* L858R group, n = 6; for *EGFR* 19del group, n = 7. Statistics was performed using Mann-Whitney test, *P* values are two-sided. (**b-d**) Box and whisker plots indicate the associations between *EGFR* L858R or 19del mutation status and labeling of EGFR(+)HX103(+) (**b**), EGFR(+) (**c**) or HX103(+) (**d**). Average values are shown as "+". The line in the box corresponds to the median. The boxes go from the upper to the lower quartiles of the data. Whiskers represent Min to Max shown with all data points. For *EGFR* L858R group, n = 8; for *EGFR* 19del group, n = 7. Statistics was performed using Mann-Whitney test, *P* values are two-sided. Source data are provided as a Source Data file.

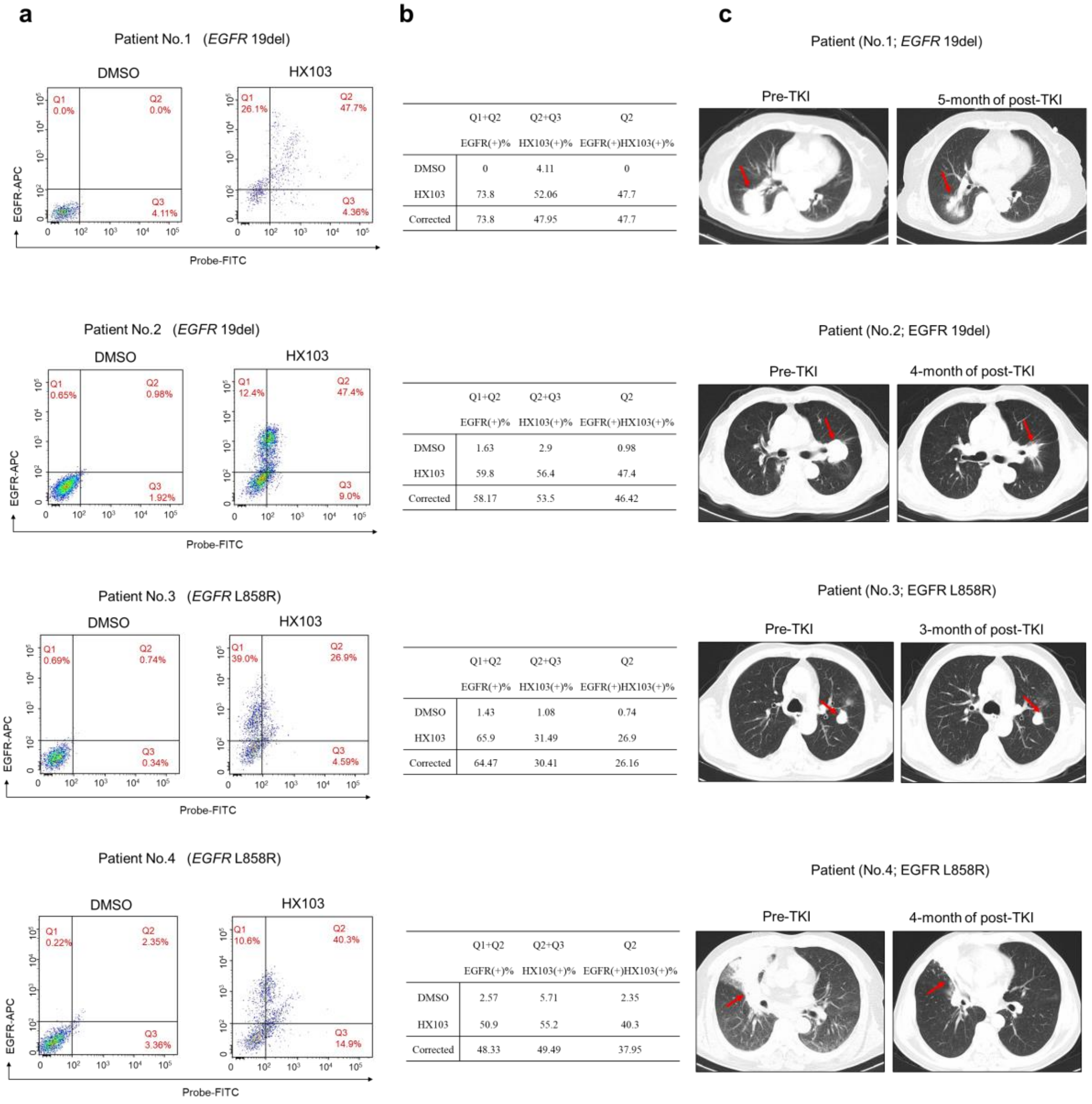

	Q1+Q2 EGFR(+)%	Q2+Q3 HX103(+)%	Q2 EGFR(+)HX103(+)%
DMSO	0	4.11	0
HX103	73.8	52.06	47.7
Corrected	73.8	47.95	47.7

	Q1+Q2 EGFR(+)%	Q2+Q3 HX103(+)%	Q2 EGFR(+)HX103(+)%
DMSO	1.63	2.9	0.98
HX103	59.8	56.4	47.4
Corrected	58.17	53.5	46.42

	Q1+Q2 EGFR(+)%	Q2+Q3 HX103(+)%	Q2 EGFR(+)HX103(+)%
DMSO	1.43	1.08	0.74
HX103	65.9	31.49	26.9
Corrected	64.47	30.41	26.16

	Q1+Q2 EGFR(+)%	Q2+Q3 HX103(+)%	Q2 EGFR(+)HX103(+)%
DMSO	2.57	5.71	2.35
HX103	50.9	55.2	40.3
Corrected	48.33	49.49	37.95

Supplementary Figure 34. Results of HX103-based FACS and CT scans for patients 1-4. (**a**) The summary of dual-parameter dot plots (HX103/EGFR antibody) determined by HX103-based FACS in biopsy samples (from Patient 1 to Patient 4). (**b**) Tables showing the calculations of percentage values for EGFR(+), HX103(+) and EGFR(+)HX103(+). (**c**) CT scans between pre-TKI treatment and post-TKI treatment showed the change in the size of tumor from NSCLC patients with *EGFR*-activating mutations who received EGFR-TKIs.

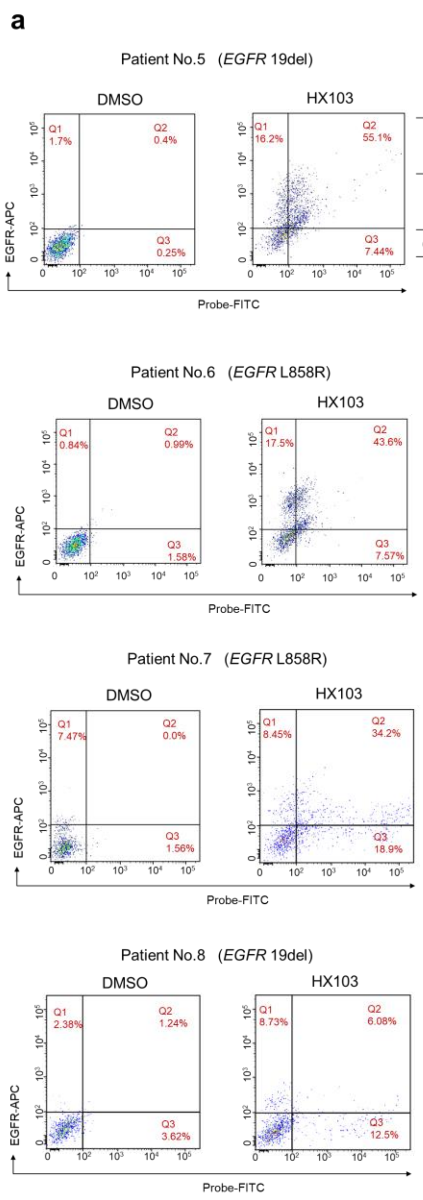

b

	Q1+Q2<br>EGFR(+)%	Q2+Q3<br>HX103(+)%	Q2<br>EGFR(+)HX103(+)%
DMSO	2.1	0.65	0.4
HX103	71.3	62.54	55.1
Corrected	69.2	61.89	54.7

	Q1+Q2<br>EGFR(+)%	Q2+Q3<br>HX103(+)%	Q2<br>EGFR(+)HX103(+)%
DMSO	1.83	2.57	0.99
HX103	61.1	51.17	43.6
Corrected	59.27	48.6	42.61

	Q1+Q2<br>EGFR(+)%	Q2+Q3<br>HX103(+)%	Q2<br>EGFR(+)HX103(+)%
DMSO	7.47	1.56	0
HX103	42.65	53.1	34.2
Corrected	35.18	51.54	34.2

	Q1+Q2<br>EGFR(+)%	Q2+Q3<br>HX103(+)%	Q2<br>EGFR(+)HX103(+)%
DMSO	3.62	4.86	1.24
HX103	14.81	18.58	6.08
Corrected	11.19	13.72	4.84

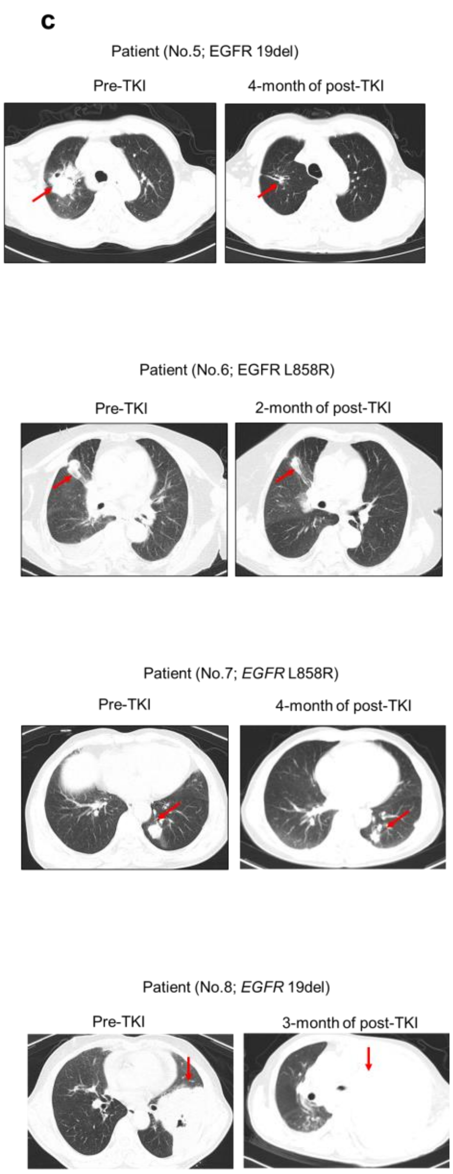

Supplementary Figure 35. Results of HX103-based FACS and CT scans for patients 5-8. (**a**) The summary of dual-parameter dot plots (HX103/EGFR antibody) determined by HX103-based FACS in biopsy samples (from Patient 5 to Patient 8). (**b**) Tables showing the calculations of percentage values for EGFR(+), HX103(+) and EGFR(+)HX103(+). (**c**) CT scans between pre-TKI treatment and post-TKI treatment showed the change in the size of tumor from NSCLC patients with *EGFR*-activating mutations who received EGFR-TKIs.

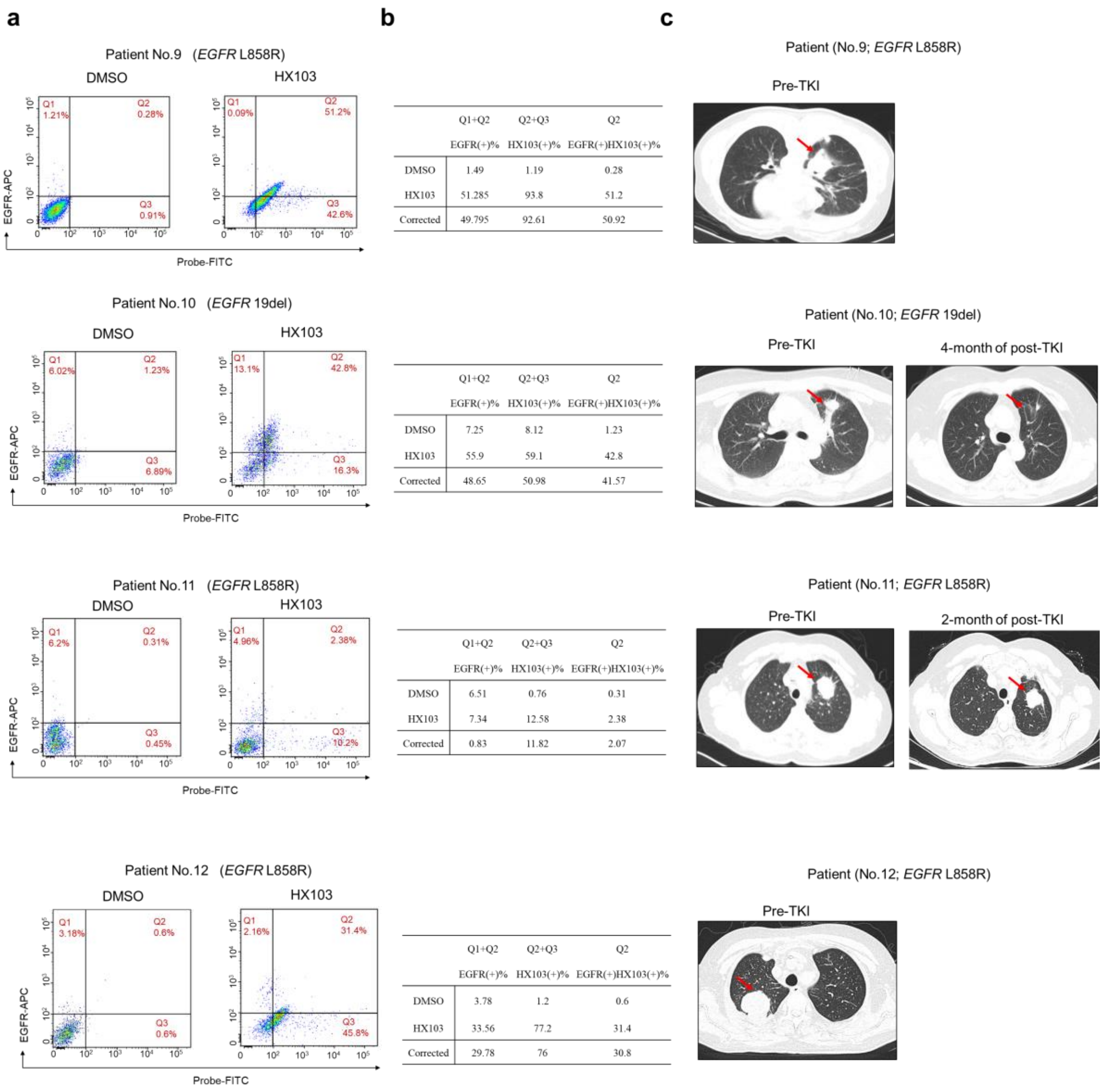

	Q1+Q2 EGFR(+)%	Q2+Q3 HX103(+)%	Q2 EGFR(+)HX103(+)%
DMSO	1.49	1.19	0.28
HX103	51.285	93.8	51.2
Corrected	49.795	92.61	50.92

	Q1+Q2 EGFR(+)%	Q2+Q3 HX103(+)%	Q2 EGFR(+)HX103(+)%
DMSO	7.25	8.12	1.23
HX103	55.9	59.1	42.8
Corrected	48.65	50.98	41.57

	Q1+Q2 EGFR(+)%	Q2+Q3 HX103(+)%	Q2 EGFR(+)HX103(+)%
DMSO	6.51	0.76	0.31
HX103	7.34	12.58	2.38
Corrected	0.83	11.82	2.07

	Q1+Q2 EGFR(+)%	Q2+Q3 HX103(+)%	Q2 EGFR(+)HX103(+)%
DMSO	3.78	1.2	0.6
HX103	33.56	77.2	31.4
Corrected	29.78	76	30.8

Supplementary Figure 36. Results of HX103-based FACS and CT scans for patients 9-12. (**a**) The summary of dual-parameter dot plots (HX103/EGFR antibody) determined by HX103-based FACS in biopsy samples (from Patient 9 to Patient 12). (**b**) Tables showing the calculations of percentage values for EGFR(+), HX103(+) and EGFR(+)HX103(+). (**c**) CT scans between pre-TKI treatment and post-TKI treatment showed the change in the size of tumor from NSCLC patients with *EGFR*-activating mutations who received EGFR-TKIs. Of note, CT scans of Patient 9 and Patient 12 after EGFR-TKIs treatment were not obtained.

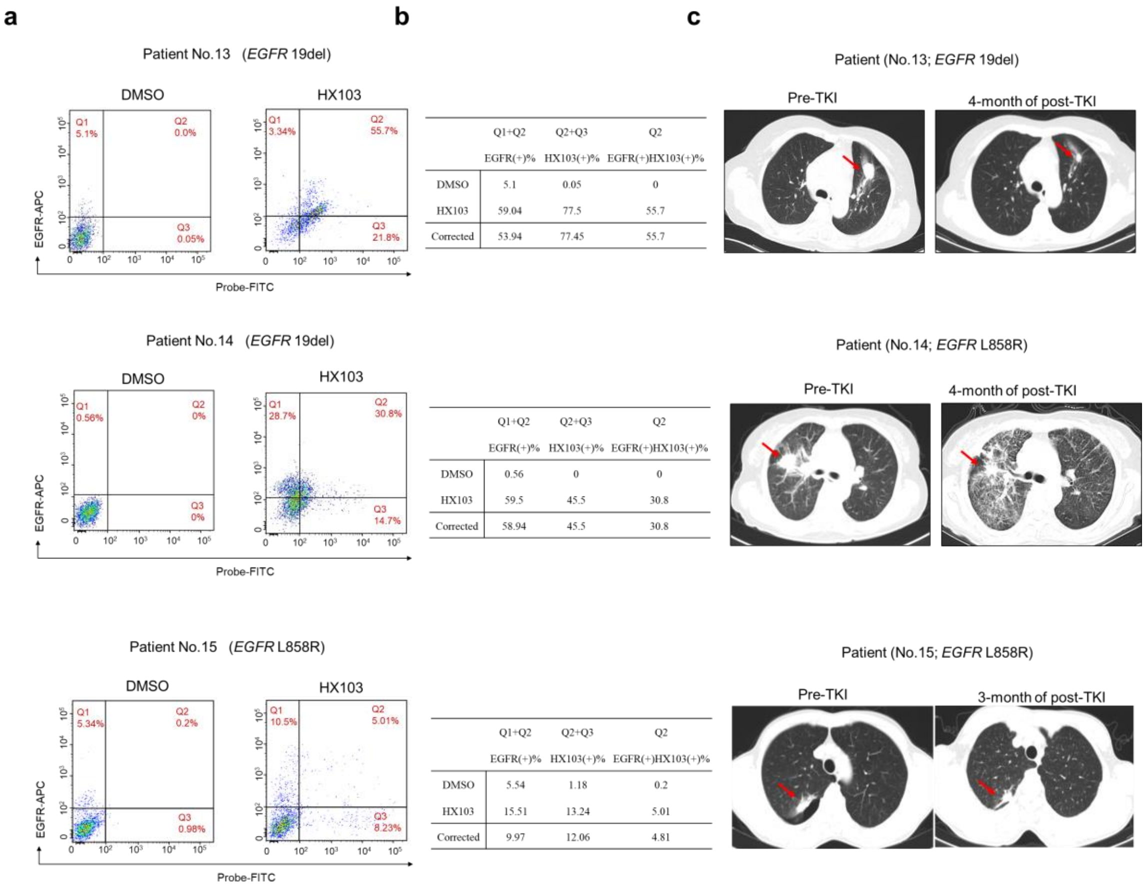

Supplementary Figure 37. Results of HX103-based FACS and CT scans for patients 13-15. (**a**) The summary of dual-parameter dot plots (HX103/EGFR antibody) determined by HX103-based FACS in biopsy samples (from Patient 13 to Patient 15). (**b**) Tables showing the calculations of percentage values for EGFR(+), HX103(+) and EGFR(+)HX103(+). (**c**) CT scans between pre-TKI treatment and post-TKI treatment showed the change in the size of tumor from NSCLC patients with *EGFR*-activating mutations who received EGFR-TKIs.

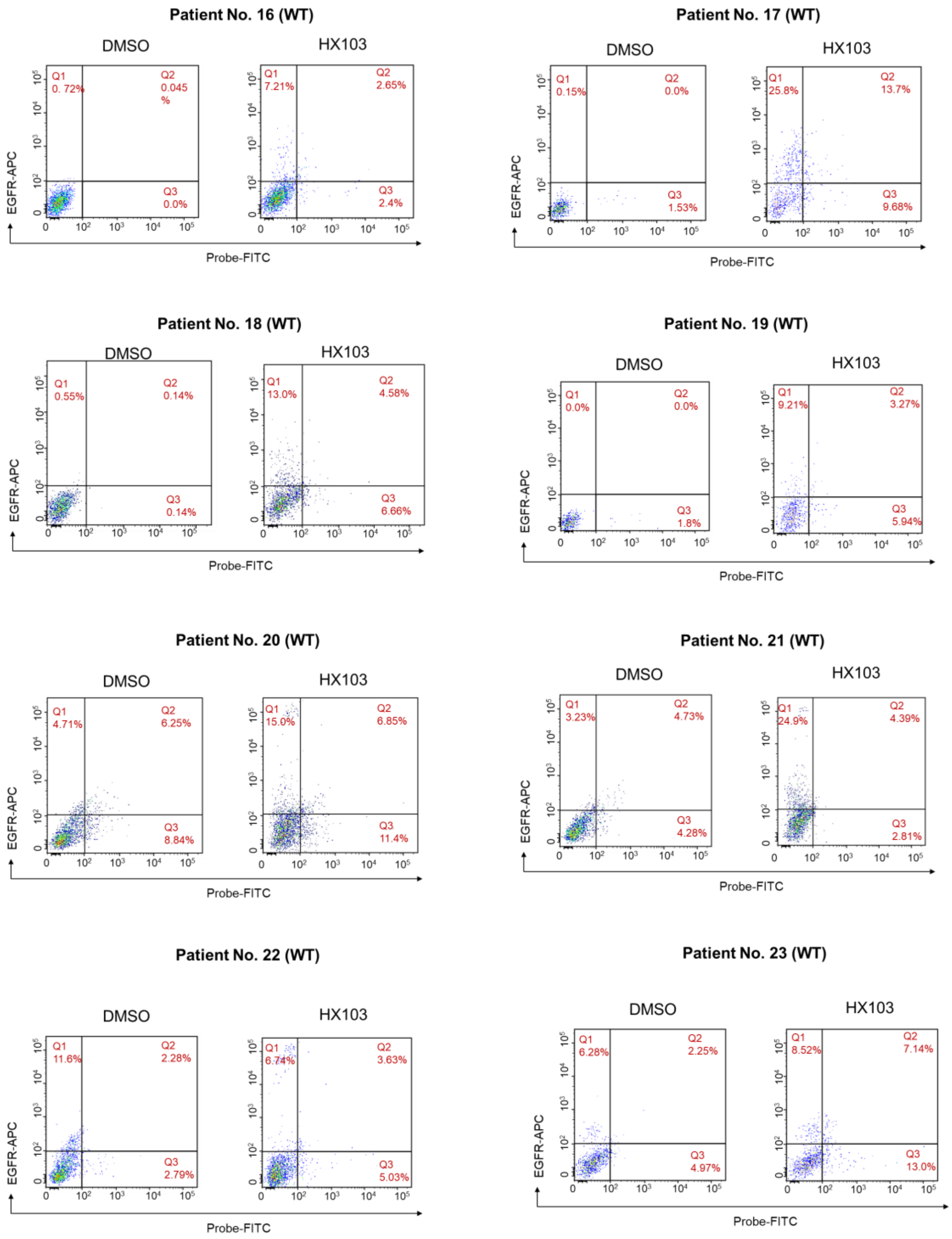

Supplementary Figure 38. The summary of dual-parameter dot plots (HX103/EGFR antibody) determined by HX103-based FACS in *EGFR* gene mutation negative biopsy samples (from Patient 16 to Patient 23).

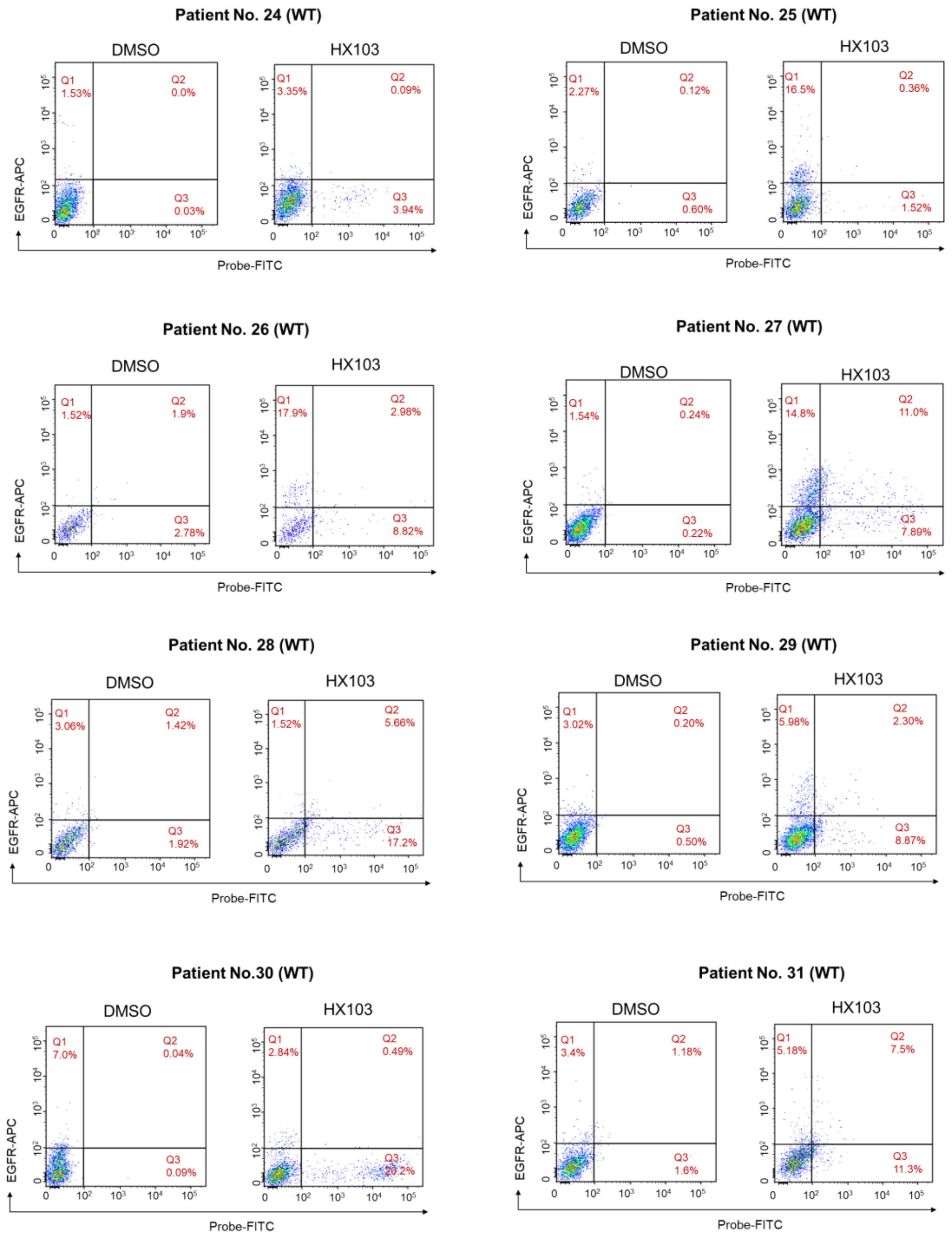

Supplementary Figure 39. The summary of dual-parameter dot plots (HX103/EGFR antibody) determined by HX103-based FACS in *EGFR* gene mutation negative biopsy samples (from Patient 24 to Patient 31).

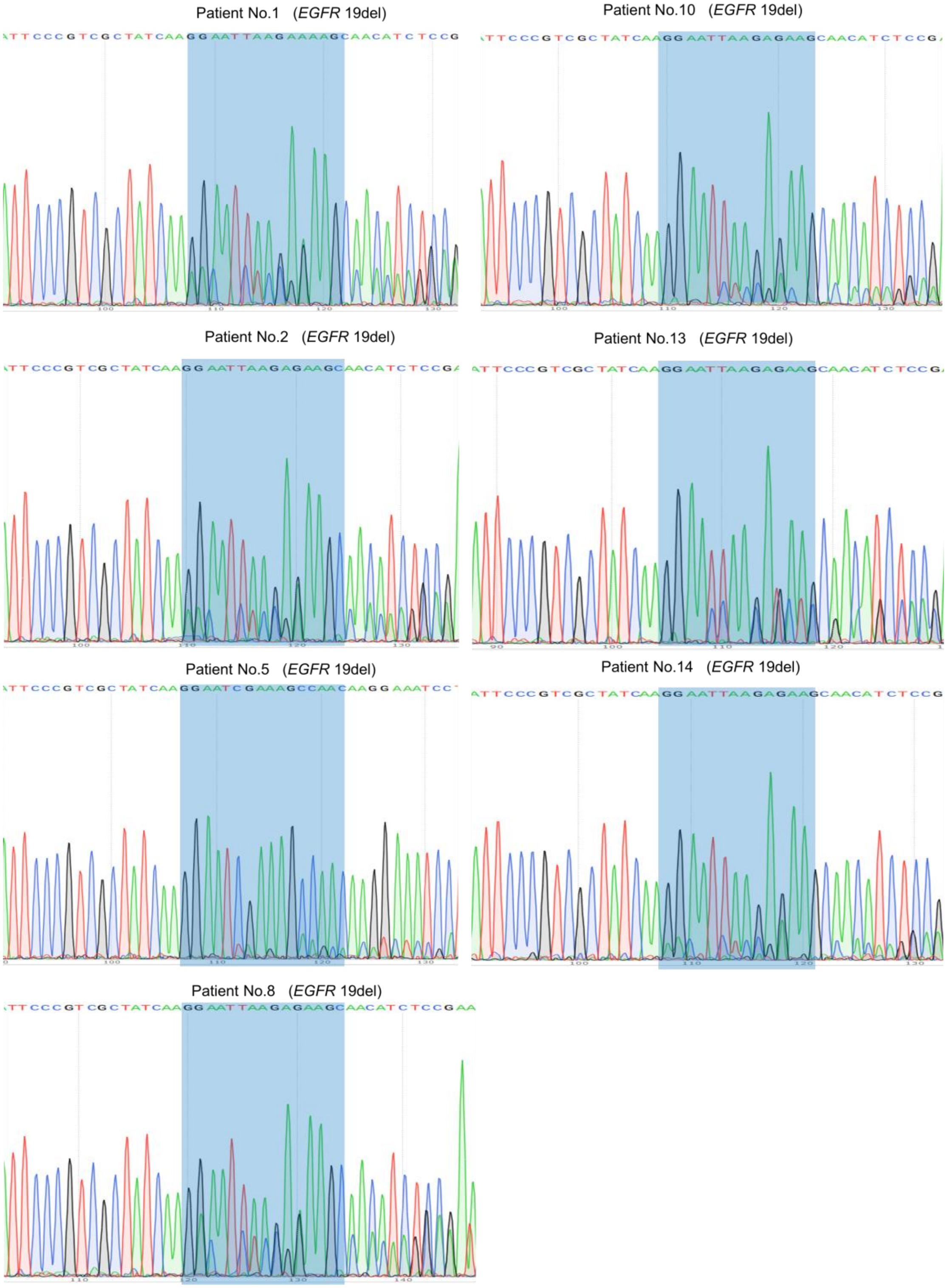

Supplementary Figure 40. The summary of gene sequencing confirmation for biopsy samples with *EGFR* 19del. Start of the exon 19 (GGAATTAAGAGAAGC) deletion is indicated by the blue area.

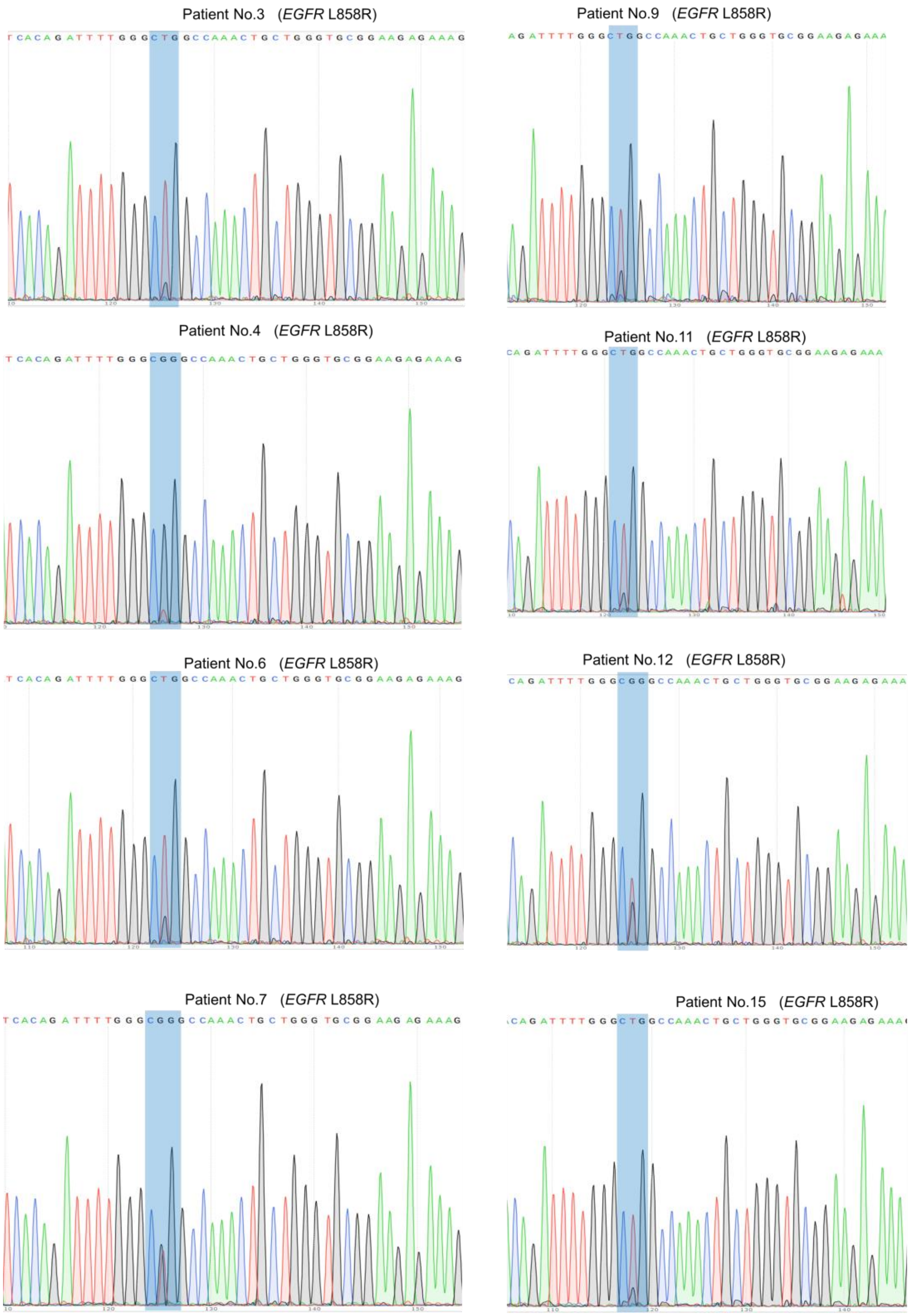

Supplementary Figure 41. The summary of gene sequencing confirmation for biopsy samples with *EGFR* L858R. Mutation point is indicated by the blue area.

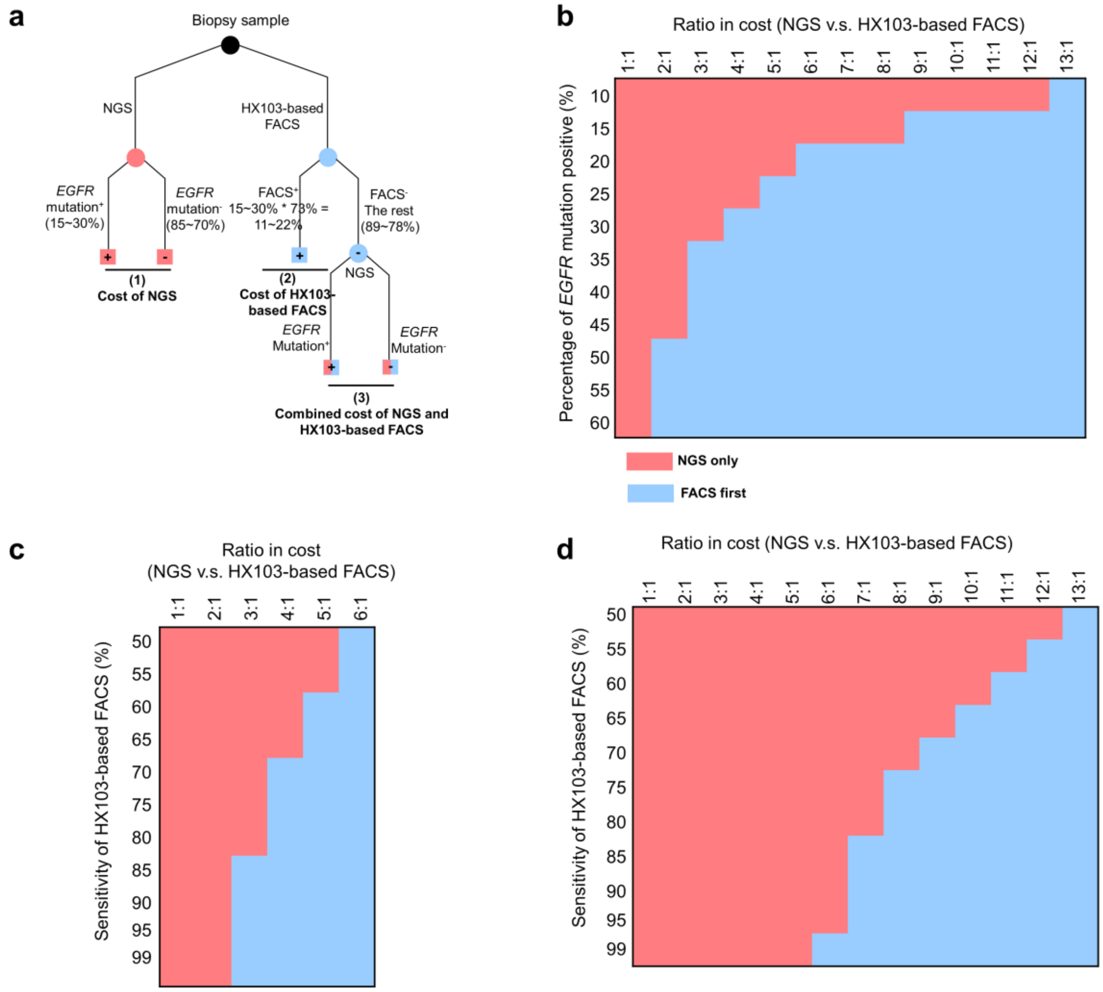

Supplementary Figure 42. Cost-benefit analysis for HX103-based FACS analysis. (**a**) A decision process for determining under which conditions upfront *EGFR* mutation testing with HX103-based FACS would be economically preferable. Of note, 73% is the sensitivity between HX103-based FACS and *EGFR* gene mutations in biopsy sample. (**b**) Cost ratio between NGS and HX103-based FACS in correlation with the proportion of *EGFR* mutations in a given population. (**c**) Cost ratio between NGS and HX103-based FACS in correlation with the sensitivity of the HX103-based FACS assay taking into account the proportion of *EGFR* mutations in Asian population (~30%). (**d**) Cost ratio between NGS and HX103-based FACS in correlation with the sensitivity of the HX103-based FACS assay taking into account the proportion of *EGFR* mutations in U.S./European population (~15%).

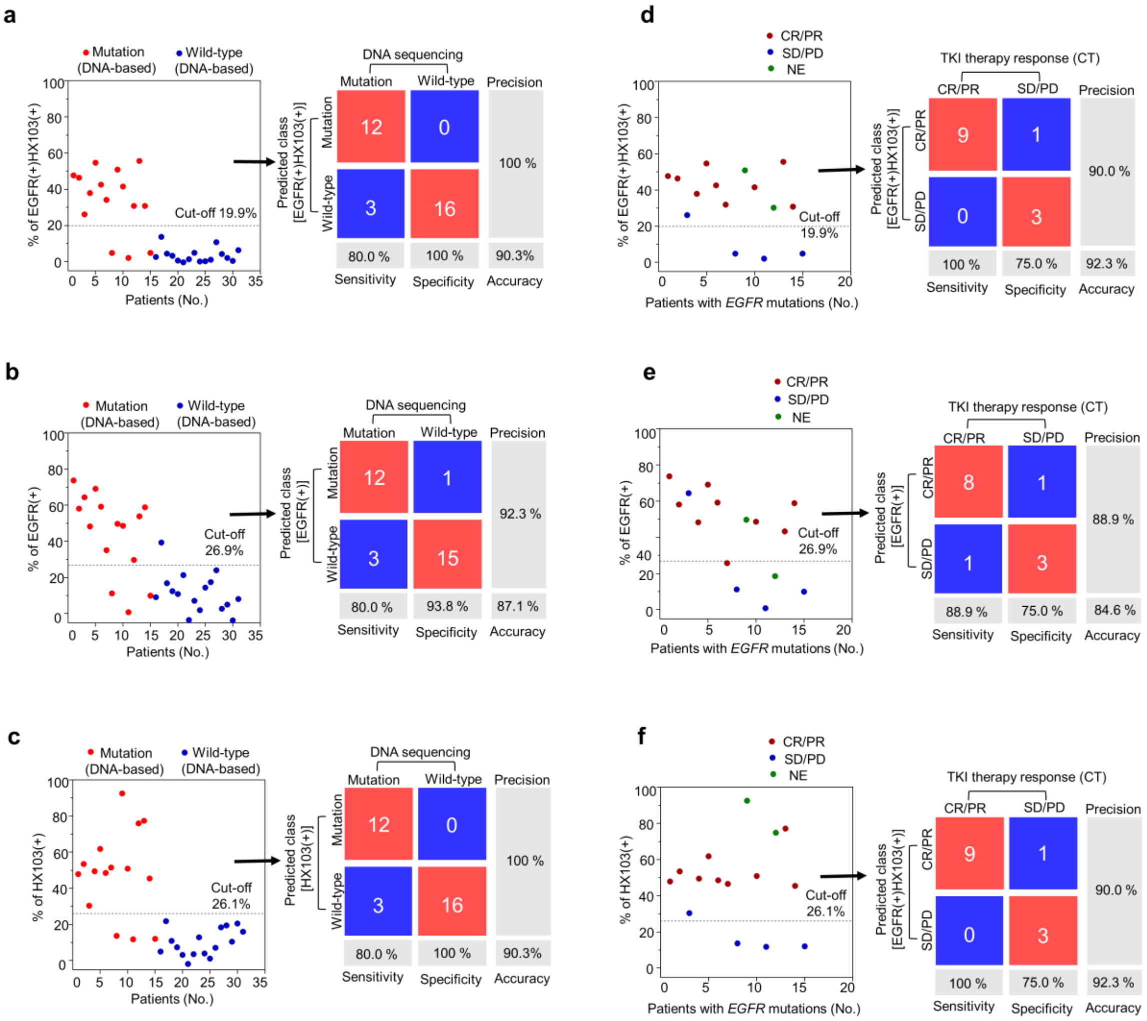

Supplementary Figure 43. Comparison of HX103-based FACS analysis with different cut-off points. (**a**-**c**) Comparing HX103-based FACS analysis and *EGFR*-activating mutations (DNA-based method) in 31 biopsy samples by using the cut-off points of EGFR(+)HX103(+) ≥ 19.9% (**a**), EGFR(+) ≥ 26.9% (**b**) and HX103(+) ≥ 26.1% (**c**) determined from Supplementary Fig. 31a. (**d**-**f**) Comparing HX103-based FACS analysis with cut-off points in (a-c) and EGFR-TKI therapy response (determined by CT imagines). Of note, therapy responses of 2 patients (9 and 12) were not evaluated, and these cut-off points were generated only with 31 biopsy samples.

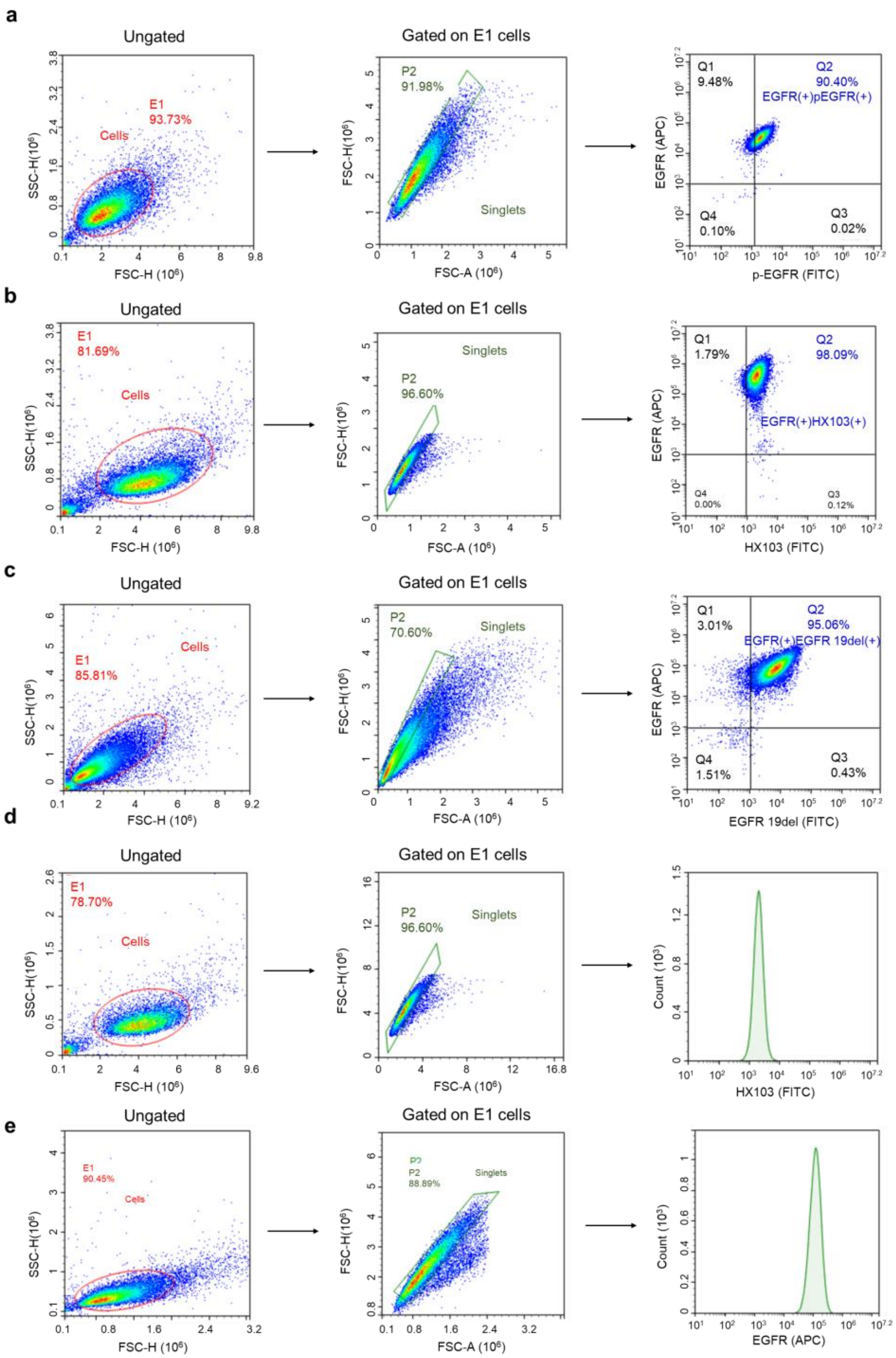

Supplementary Figure 44. Gating strategies of flow cytometric experiments in culture cells. (**a-c**) Gating strategy for measuring the percent of EGFR autophosphorylation (a), HX103 labeling (b) and EGFR mutants (c) in living cells. Events were gated by forward and site scatter (cells), side scatter area (singlets) and the threshold for EGFR-positive and pEGFR/HX103/EGFR mutant cells were determined based on background fluorescence for isotype control. This gating strategy applies to Fig 3f-h and Supplementary Fig. 9-12 and 15-17. (**d-e**) Gating strategy for measuring fluorescence intensity of HX103 (d) and antibody of EGFR (e). This gating strategy applies to Fig 3c-e and Supplementary Fig. 7 and 14.

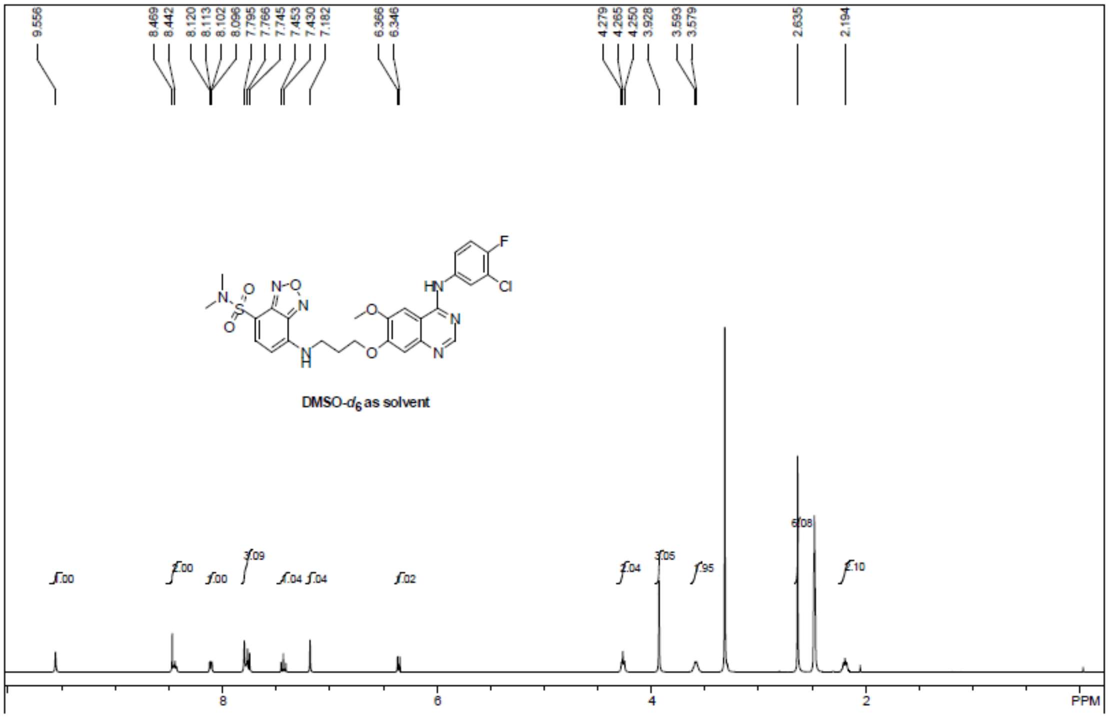

Supplementary Figure 45. ^{1}H-NMR spectrum of 7-((3-((4-((3-chloro-4-fluorophenyl)amino)-6-methoxyquinazolin-7-yl)oxy)propyl)amino)-N,N-dimethylbenzo[c][1,2,5]oxadiazole-4-sulfonamide (**9**, HX103).

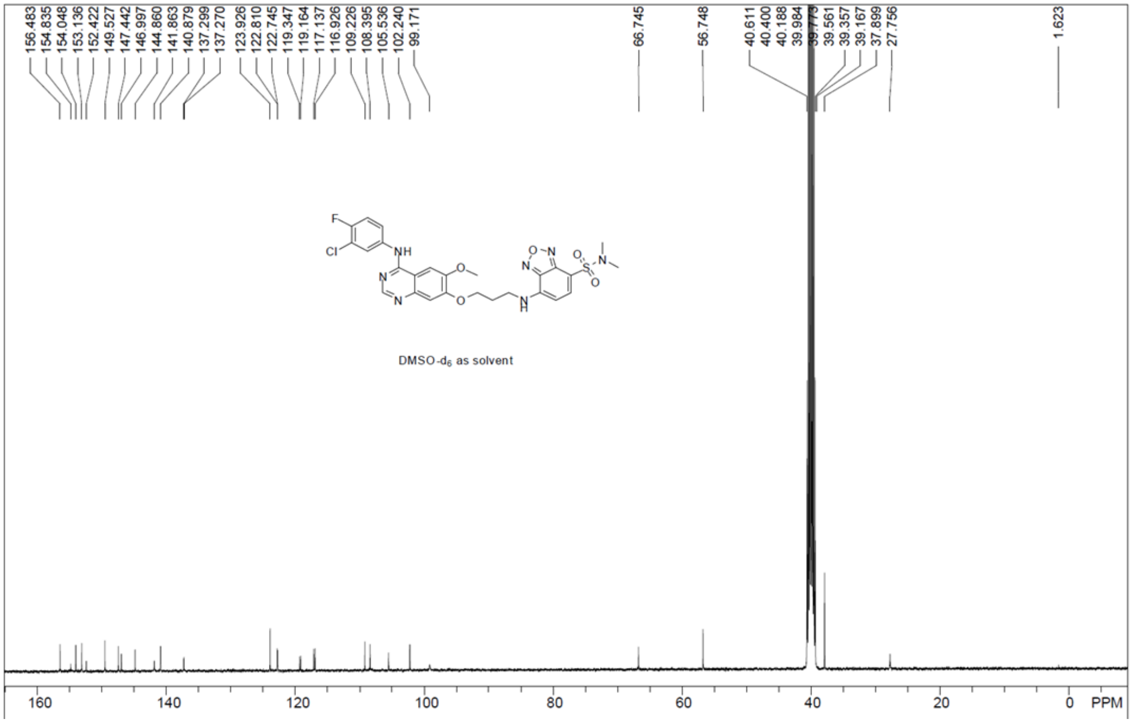

Supplementary Figure 46. ^{13}C-NMR spectrum of 7-((3-((4-((3-chloro-4-fluorophenyl)amino)-6-methoxyquinazolin-7-yl)oxy)propyl)amino)-N,N-dimethylbenzo[c][1,2,5]oxadiazole-4-sulfonamide (**9**, HX103).

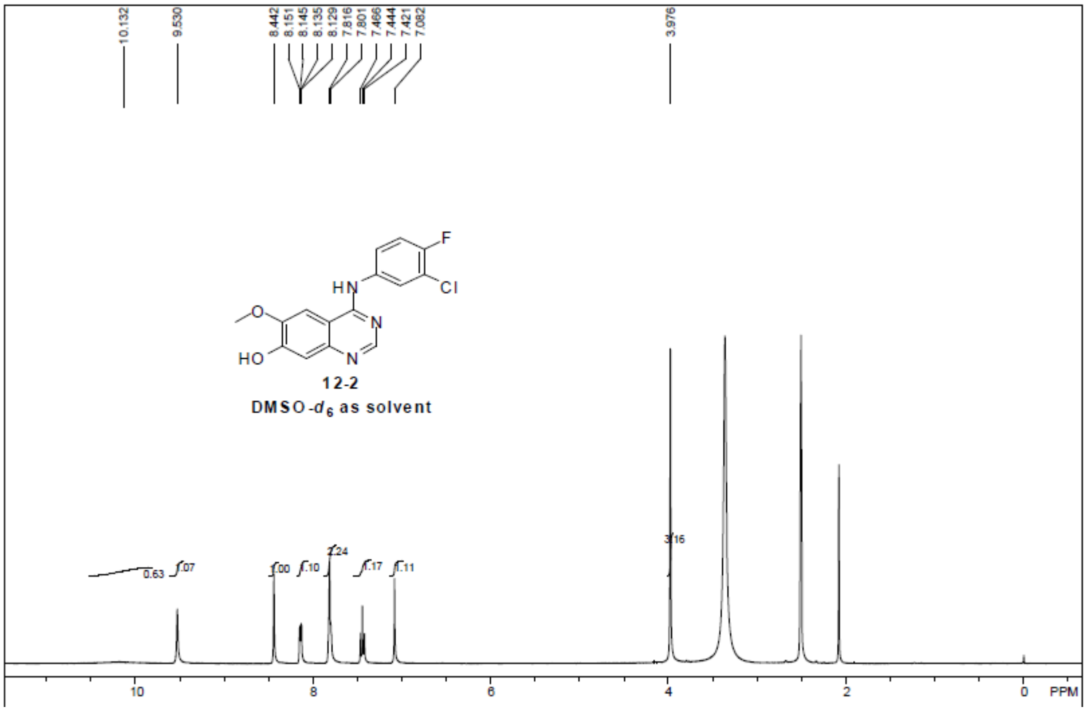

Supplementary Figure 47. ^{1}H-NMR spectrum of 4-((3-chloro-4-fluorophenyl)amino)-6-methoxyquinazolin-7-ol (**4**).

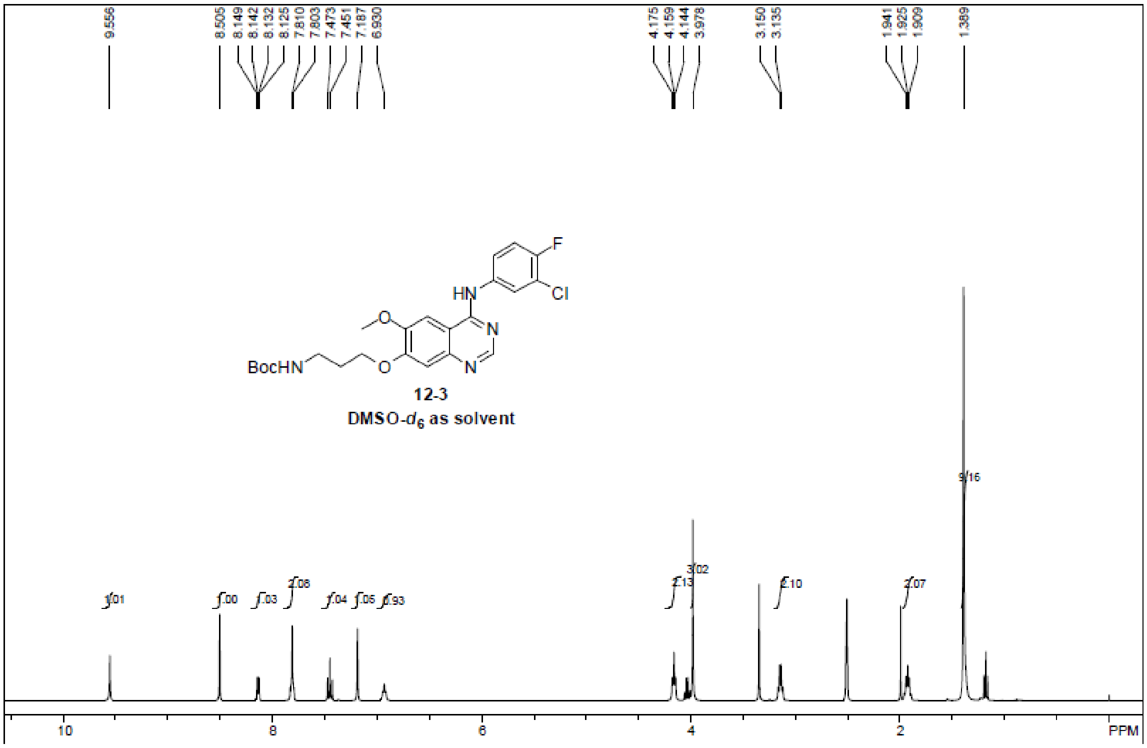

Supplementary Figure 48. ^{1}H-NMR spectrum of *tert*-Butyl (3-((4-((3-chloro-4-fluorophenyl)amino)-6-methoxyquinazolin-7-yl)oxy)propyl)carbamate (**6**).

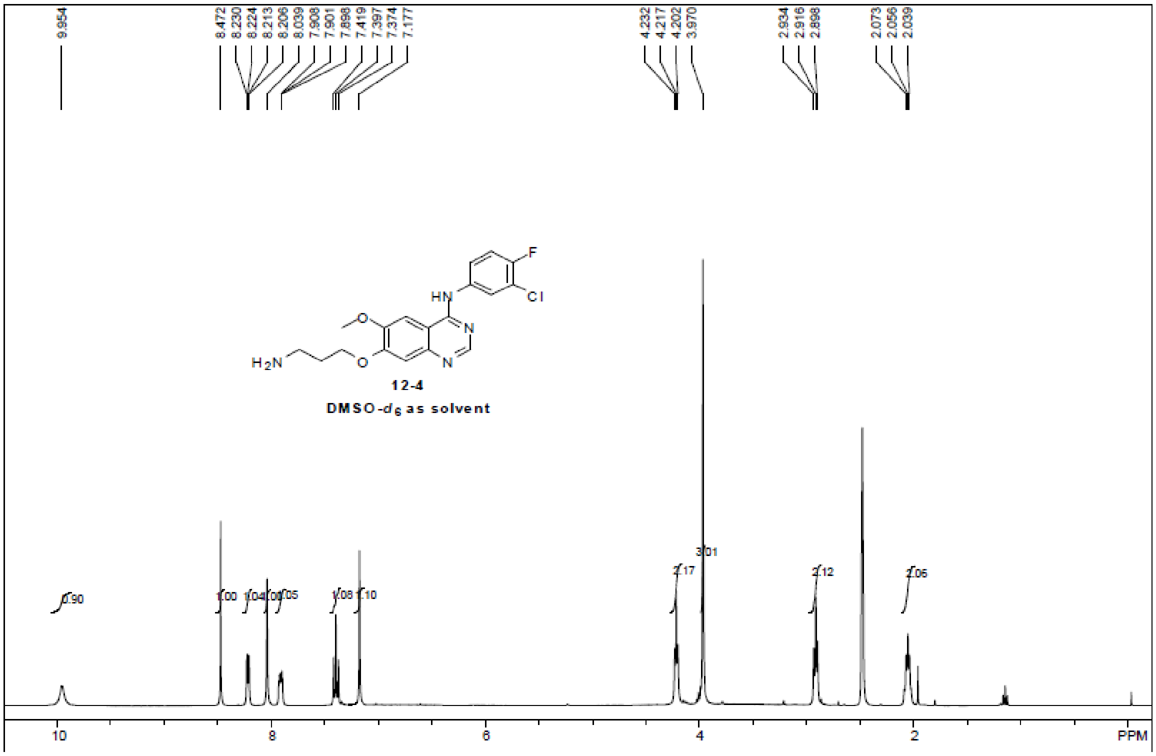

Supplementary Figure 49. ^{1}H-NMR spectrum of 7-(3-aminopropoxy)-N-(3-chloro-4-fluorophenyl)-6-methoxyquinazolin-4-amine (**7**).

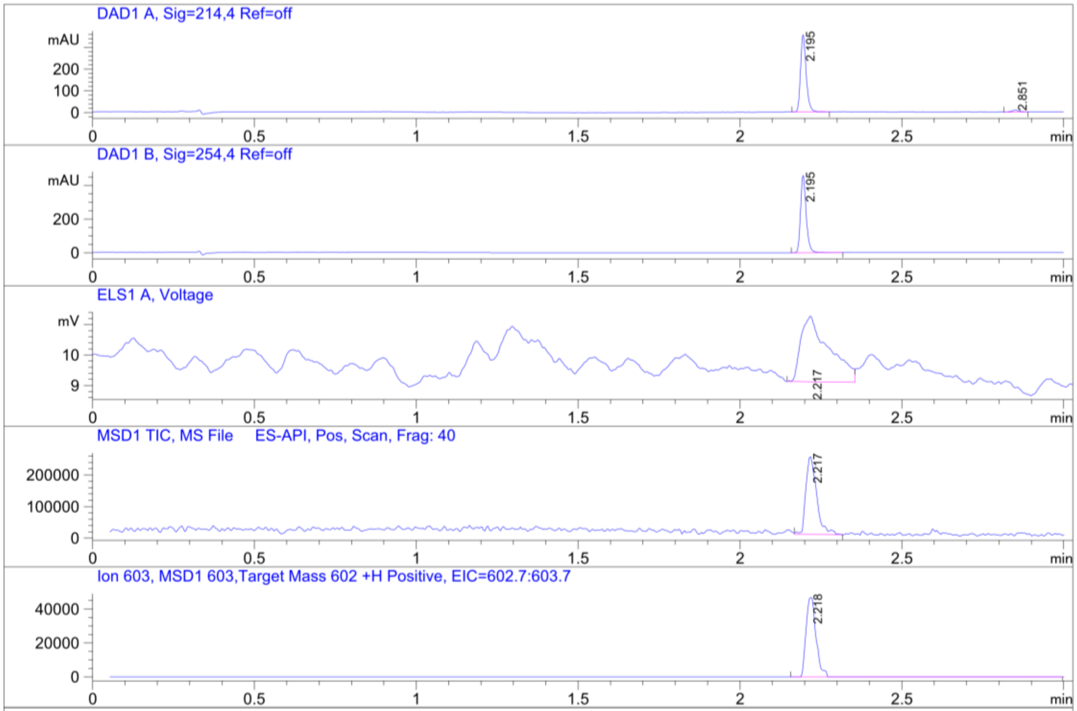

Integration Results for DAD1 A, Sig=214,4 Ref=off

RetTim	Width	Area	Height	Area%
2.20	0.02	425.98	355.11	96.97
2.85	0.02	13.31	8.70	3.03

Integration Results for DAD1 B, Sig=254,4 Ref=off

RetTim	Width	Area	Height	Area%
2.20	0.02	551.29	458.99	100.00

Integration Results for ELS1 A, Voltage

RetTim	Width	Area	Height	Area%
2.22	0.08	13.05	2.16	100.00

Integration Results for MSD1 TIC, MS File

RetTim	Width	Area	Height	Area%

Ret. Time: 2.22 <<<< POSITIVE SPECTRA >>>>

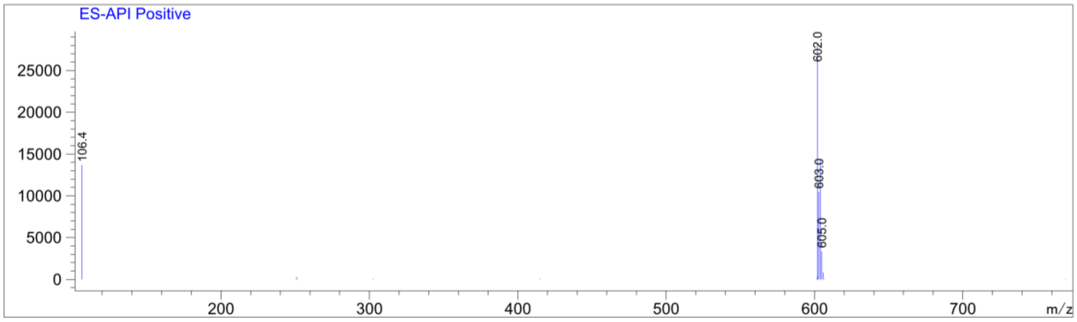

Supplementary Figure 50. LC-MS spectrum of 7-((3-((4-((3-chloro-4-fluorophenyl)amino)-6-methoxyquinazolin-7-yl)oxy)propyl)amino)-N,N-dimethylbenzo[c][1,2,5]oxadiazole-4-sulfonamide (**9**, HX103).

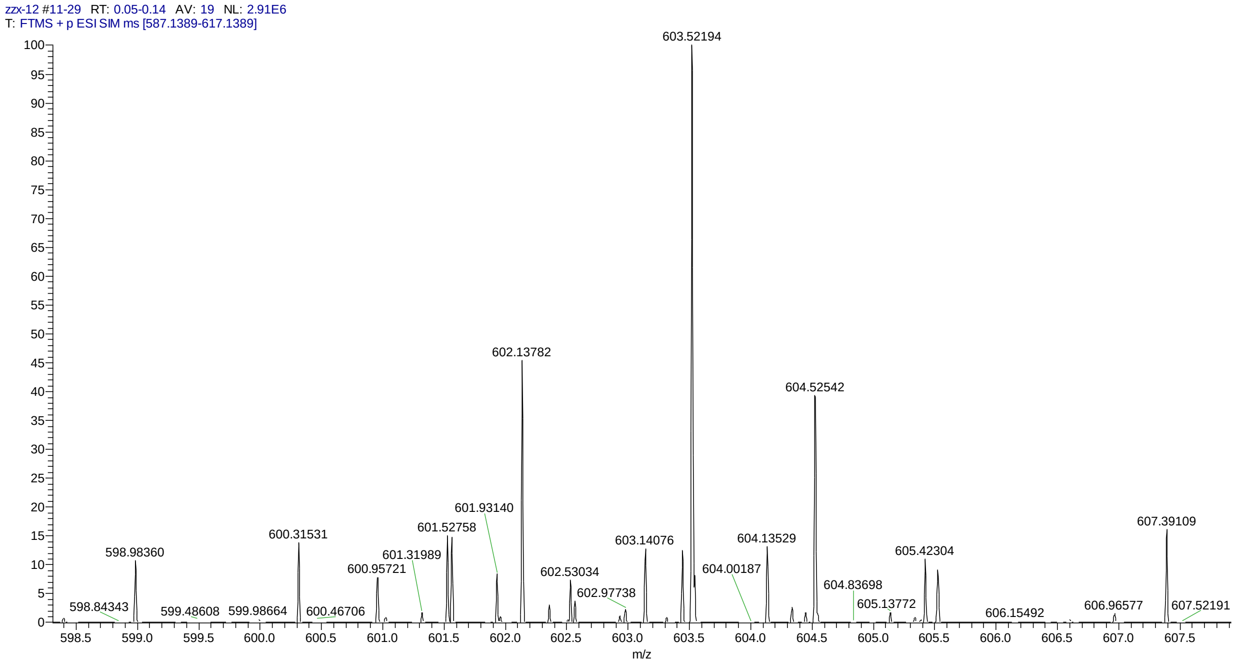

Supplementary Figure 51. HRMS spectrum of 7-((3-((4-((3-chloro-4-fluorophenyl)amino)-6-methoxyquinazolin-7-yl)oxy)propyl)amino)-N,N-dimethylbenzo[c][1,2,5]oxadiazole-4-sulfonamide (**9**, HX103).

Supplementary Tables

Supplementary Table 1. Binding energy of HX103 and gefitinib to EGFR wild-type and mutants (L858R, L858R/T790M). Data represent average values with the standard error of mean (SEM).

Target	Ligand	Vander Waal energy (kJ/mol)	Electrostatic energy (kJ/mol)	Polar solvation energy (kJ/mol)	SASA energy (kJ/mol)	Binding energy (kJ/mol)
EGFR L858R	Gefitinib	-106±4	-14±3	58±2	-10±0	-72±4
	HX103	-104±4	-9±4	57±2	-10±1	-67±3
EGFR wild-type	Gefitinib	-98±3	-15±3	60±2	-13±1	-65±5
	HX103	-102±4	-12±2	63±3	-9±1	-60±4
EGFR L858R/T790 M	Gefitinib	-84±3	-16±2	59±2	-12±1	-53±3
	HX103	-89±3	-14±3	61±3	-13±0	-55±4

Supplementary Table 2. Absorbance, excitation and emission maximum and quantum yield of HX103.

Compound	λmax (nm)	λex (nm)	λem (nm)	ϕ(%) PBS	ϕ(%) DMSO
HX103	340/440 (PBS)<br>342/438 (DMSO)	340/440	570	5.8	42.4

Supplementary Table 3. Inhibitory values for HX103 and gefitinib towards recombinant EGFR wild-type, EGFR L858R, EGFR 19del and EGFR T790M. Data represent average values with 95% Confidence Intervals (CI), n=3 per group.

	HX103	Gefitinib
	IC_{50} (95% CI) [nM]	IC_{50} (95% CI) [nM]
EGFR wild-type	4.0 (3.6-4.4)	1.9 (1.7-2.2)
EGFR L858R	1.5 (1.3-1.9)	1.7 (1.5-1.9)
EGFR 19del	1.3 (1.2-1.5)	1.2 (1.0-1.4)
EGFR T790M	977 (832-1147)	787 (694-893)

Supplementary Table 4. The cytotoxicity of HX103 and gefitinib in cancer cells. Data represent average values, n=3 per group.

Cytotoxicity (IC_{50}, [μM], n=3)			
Cells	EGFR status	HX103	Gefitinib
A431	wild-type	26	5.7
A549	wild-type	18	33
Hela	wild-type	>100	28
HCC827	19del	9.3	1.5
H1975	L858R/T790M	45	22
PC-9	19del	1.8	0.5

Supplementary Table 5. The summary of percentage values for HX103(+), EGFR(+) and HX103(+)EGFR(+) in surgical samples (including 23 tumor and 23 adjacent normal samples) (Supplementary Fig. 20).

The ratios of cell populations (%) (Background-correction with DMSO-treated sample)							
Primary tumor tissue				Adjacent normal tissue			
Samples	HX103 (+)	EGFR (+)	EGFR(+) HX103(+)	Samples	HX103 (+)	EGFR (+)	EGFR(+) HX103(+)
#1-T	39.72	72.72	37.65	#1-N	5.959	25.282	5.242
#2-T	57.994	98.854	62.204	#2-N	0.091	16.776	0.061
#3-T	21.923	93.913	22.183	#3-N	2.83	8.44	0.95
#4-T	56.54	89.96	56.19	#4-N	0.117	25.337	0.021
#5-T	38.985	85.155	40.635	#5-N	1.119	9.724	0.392
#6-T	61.81	90.54	60.93	#6-N	6.793	14.09	3.342
#7-T	2.402	11.172	1.692	#7-N	0.29	5.63	0.18
#8-T	90.573	21.57	22.6	#8-N	3.43	17.5	2
#9-T	54.55	59.33	44.43	#9-N	0.579	3.979	0.019
#10-T	13.41	29.318	5.35	#10-N	0.41	12.72	0.12
#11-T	4.48	28.25	2.39	#11-N	1.2	12.02	1.02
#12-T	99.62	98.888	98.7	#12-N	-0.18	15.104	0.02
#13-T	65.67	98.18	80.28	#13-N	5.88	13.69	0.81
#14-T	0.504	14.716	0.016	#14-N	6.841	11.98	0.58
#15-T	9.842	0.262	0.032	#15-N	10.965	23.355	4.055
#16-T	1.722	0.78	0.15	#16-N	9.654	4.767	3.584
#17-T	74.682	64.78	57.8	#17-N	0.88	0.047	0
#18-T	20.064	2.52	1.7	#18-N	10.911	4.262	1.37
#19-T	2.48	5.18	0.04	#19-N	3.04	6.985	0.53
#20-T	50.02	43.19	39.77	#20-N	9.896	73.196	9.886
#21-T	22.915	13.85	7.26	#21-N	11.48	30.15	12.8
#22-T	76.359	65.139	59.969	#22-N	7.83	6.292	3.85
#23-T	97.452	79.805	79.812	#23-N	10.32	8.56	6.59

Supplementary Table 6. The calculations of MFI values of HX103 in 23 surgical tumor samples (Supplementary Fig. 21).

	MFI value			Background-corrected MFI			Normalized MFI (%)			Specific labeling of HX103
Sample	HX103	HX103 + Gefitinib	DMSO	HX103	HX103 + Gefitinib	DMSO	HX103	HX103 + Gefitinib	DMSO	HX103
#1-T	138	147	27.2	110.8	119.8	0	100	108.1	0	-8.1
#2-T	136	66.7	45.6	90.4	21.1	0	100	23.3	0	76.6
#3-T	40.3	43.4	3.7	36.5	39.6	0	100	108.4	0	-8.4
#4-T	99.1	51.6	10.6	88.5	41	0	100	46.3	0	53.6
#5-T	83.4	57.5	11.2	72.2	46.3	0	100	64.1	0	35.8
#6-T	154	60	23.2	130.8	36.8	0	100	28.1	0	71.8
#7-T	52.6	41.5	38.4	14.2	3.1	0	100	21.8	0	78.1
#8-T	236	77.9	28.5	207.5	49.4	0	100	23.8	0	76.1
#9-T	154	86.1	26.1	127.9	60	0	100	46.9	0	53.0
#10-T	76.8	61.9	40.9	35.9	21	0	100	58.4	0	41.5
#11-T	57.3	35.8	26.1	31.2	9.7	0	100	31.0	0	68.9
#12-T	286	80.1	20.3	265.7	59.8	0	100	22.5	0	77.4
#13-T	139	78.2	28.5	110.5	49.7	0	100	44.9	0	55.0
#14-T	21.6	20.9	11.4	10.2	9.5	0	100	93.1	0	6.8
#15-T	65.4	21.1	45.6	19.8	-24.5	0	100	-123.7	0	223.7
#16-T	26.2	17.7	4.8	21.3	12.8	0	100	60.1	0	39.8
#17-T	148	85.6	4.8	143.1	80.7	0	100	56.4	0	43.5
#18-T	72.6	26.6	14.2	58.4	12.4	0	100	21.2	0	78.7
#19-T	47.5	29.6	8.9	38.6	20.7	0	100	53.6	0	46.3
#20-T	61.8	53.7	3.6	58.1	50.0	0	100	86.0	0	13.9
#21-T	84.4	49.4	7.9	76.4	41.4	0	100	54.2	0	45.7
#22-T	139.8	12.2	7.5	132.2	4.6	0	100	3.5	0	96.4
#23-T	162	47.6	13.1	148.8	34.4	0	100	23.1	0	76.8

Supplementary Table 7. Patient characteristics for surgical samples.

Characteristics	Total	*EGFR* L858R	*EGFR* 19del	*EGFR* (WT)
Patients, n (%)	23	8 (35%)	4 (17%)	11 (48%)
Ages (years)				
Median	61	57	65	62
Range	38-80	38-77	49-82	45-80
Sex (male/female)	9:14	1:3	3:1	4:7
Stage (AJCC)				
I	13	5	3	5
II	8	2	1	5
III	1	1	0	0
IV	1	0	0	1
EGFR(+)HX103(+)%[a]				
≥30.1	12(52%)	7(30%)	3(13%)	2(9%)
<30.1	11(48%)	1(4%)	1(4%)	9(39%)

[a] Threshold value (30.1%) was determined from the receiver operating characteristic curve.

Supplementary Table 8. Clinicopathological and treatment characteristics for patients with NSCLC.[a,b]

Patient (No.)	Age	Sex	Stage (AJCC)[a]	Histology	First-Line Treatment[b]	Response in lesion size (%)
1	74	F	IV	Adenocarcinoma	Afatinib	-36.2
2	76	M	IV	Adenocarcinoma	Afatinib	-36.4
3	64	M	IV	Adenocarcinoma	Afatinib	-1.7
4	65	M	IV	Adenocarcinoma	Dacomitinib	-31.6
5	63	M	IV	Adenocarcinoma	Gefitinib	-72.8
6	69	F	IV	Adenocarcinoma	Dacomitinib	-39.4
7	75	M	IV	Adenocarcinoma	Gefitinib	-46.6
8	64	M	IV	Adenocarcinoma	Dacomitinib	21.4
9	52	F	IV	Adenocarcinoma	Icotinib	NE*
10	49	F	IV	Adenocarcinoma	Icotinib	-55.4
11	63	F	IV	Adenocarcinoma	Icotinib	-12.6
12	86	M	IV	Adenocarcinoma	Erlotinib	NE*
13	69	F	IV	Adenocarcinoma	Gefitinib	-60.0
14	64	F	IV	Adenocarcinoma	Gefitinib	-32.5
15	57	M	IV	Adenocarcinoma	Afatinib	-12.5
16	76	F	III	Adenocarcinoma	Immunotherapy	-11.7
17	66	M	IV	Adenocarcinoma	Immunotherapy	-41.0
18	76	F	IV	Adenocarcinoma	Chemotherapy	-46.8
19	62	M	III	Adenocarcinoma	Chemotherapy & immunotherapy	-29.4
20	67	M	IV	Adenocarcinoma	Immunotherapy	-25.9
21	70	M	III	Adenocarcinoma	None	NE*
22	66	M	IV	Adenocarcinoma	Immunotherapy	NE*
23	63	M	III	Adenocarcinoma	Chemotherapy	NE*
24	70	M	III	Squamous carcinoma	Chemotherapy	-5.5
25	53	F	IV	Adenocarcinoma	Chemotherapy & immunotherapy	NE*
26	87	M	II	Adenocarcinoma	Immunotherapy & Radiotherapy	-17.3
27	69	M	IV	Adenocarcinoma	Chemotherapy & immunotherapy	-20.1
28	54	M	IV	Adenocarcinoma	None	NE*
29	65	M	IV	Adenocarcinoma	Chemotherapy & immunotherapy	24.0
30	55	M	III	Squamous carcinoma	Chemotherapy & immunotherapy	-39.2
31	68	M	IV	Squamous carcinoma	Chemotherapy & immunotherapy	-36.5

[a]AJCC (American Join Committee on Cancer), EGFR L858R (EGFR exon 21 point mutation), EGFR 19del (EGFR exon 19 deletions), WT (wild-type). [b]NSCLC patients with *EGFR*-activating mutations were selected for EGFR-TKIs therapy, while patients without any *EGFR* mutations were treated with other methods, including chemotherapy, radiotherapy, and immunotherapy. *NE indicates not evaluable.

Supplementary Table 9. The cost of HX103-based FACS approach for per biopsy sample.

No.	Reagents	Price ($)	Amount	Use (per biopsy sample)	Output	Price for per sample ($)
1	CD45 antibody	250.7	100 µL	0.5 µL	200	1.25
2	EGFR antibody (conjuated with Alexa Fluor 647)	528.6	100 µL	1.0 µL	100	5.29
3	Alexa fluor 647 Rabbit IgG, monoclonal Isotype	514.3	100 µL	0.5 µL	200	2.57
4	Fixable viability kit	214.3	100 µL	1.0 µL	100	2.14
5	Fc block	111.4	100 µL	2.0 µL	50	2.23
6	HX103	1142.9	20 mg (3322 µL of 10 mM stock solution was obtained)	0.5 µL	6644	0.17
Total						13.65

Supplementary Methods

Material and general remarks

The reactions were performed using oven-dried glassware and dry solvents. All reagents were purchased from commercial sources and used without further purification unless noted otherwise. NMR spectra (^{1}H and ^{13}C) were recorded on a Bruker AV spectrometer at 400 (^{1}H) and 101 (^{13}C) MHz using $(CD_3)_2SO$ as solvent, unless stated otherwise. Chemical shift values are reported in ppm with tetramethylsilane or solvent resonance as the internal standard ($(CD_3)_2SO$, δ 2.50 for ^{1}H, δ 39.52 for ^{13}C). High-resolution mass spectra (HRMS) were recorded on a Thermo Scientific LTQ Orbitrap XL. HPLC purification was performed on a preparative LC-MS system (Agilent 1200 series) with an Agilent 6110 or 6120 mass spectrometer detector. The general LC-MS condition was as follows: Waters X Bridge C18 column (50 mm x 4.6 mm x 3.5 μm), Flow Rate: 2.0 mL/min, the column temperature: 40°C. The purity of the final compounds was determined by a percentage ratio between the areas of the main peak and of possible impurities. All tested compounds were determined to be at least 95% pure by this method.

Synthesis procedures

Synthesis of 7-(benzyloxy)-N-(3-chloro-4-fluorophenyl)-6-methoxyquinazolin-4-amine (**3**). To the solution of 7-(benzyloxy)-4-chloro-6-methoxyquinazoline (**1**) (15.0 g, 49.9 mmol) in i-PrOH (200 mL) was added 3-chloro-4-fluoroaniline (**2**) (8.7 g, 59.9 mmol), the reaction mixture was stirred under reflux for 4 h. When the reaction was completed and the mixture was cooled to room temperature, filtered and the solid was collected and washed with i-PrOH and ether. Dried *in vacuum* to give the crude compound **3** as a white solid (18.7 g, yield 91%). LC-MS m/z: calculated

for $C_{22}H_{17}ClFN_3O_2$ $[M+H]^+$ 410.1, found: 410.4. ^{1}H-NMR (400 MHz, DMSO-d_6) δ 11.23 (s, 1H), 8.88 (s, 1H), 8.28 (s, 1H), 8.07 (s, 1H), 7.47 (s, 8H), 5.38 (s, 2H), 4.05 (s, 3H).

Synthesis of 4-((3-chloro-4-fluorophenyl)amino)-6-methoxyquinazolin-7-ol (**4**). A solution of compound **3** (18.7 g, 45.6 mmol) in TFA (90 mL) was stirred at 70 °C for 90 min. The reaction mixture was monitored by LC-MS. The reaction mixture was cool to room temperature. Adjust the pH to 9-10 with ammonium hydroxide solution. The suspension was filtered and washed with water and ether, dried *in vacuum* to afford crude compound **4** as a white solid (14 g, yield 96%). LC-MS m/z: calculated for $C_{15}H_{11}ClFN_3O_2$ $[M+H]^+$ 320.1, found: 320.2. ^{1}H-NMR (400 MHz, DMSO-d_6) δ 10.22 (s, 1H), 9.53 (s, 1H), 8.44 (s, 1H), 8.14 (dd, *J* = 6.8, 2.4 Hz, 1H), 7.81 (d, *J* = 6.1 Hz, 2H), 7.44 (t, *J* = 9.1 Hz, 1H), 7.08 (s, 1H), 3.98 (s, 3H).

Synthesis of *tert*-butyl (3-((4-((3-chloro-4-fluorophenyl)amino)-6-methoxyquinazolin-7-yl)oxy)propyl)carbamate (**6**). A solution of compound **4** (1.5 g, 4.7 mmol), *tert*-butyl (3-bromopropyl)carbamate (**5**) (1.2 g, 5.16 mmol) and K_2CO_3 (3.2 g, 23.5 mmol) in DMF (10 mL) was stirred at 80°C for 4 h. The reaction mixture was monitored by LC-MS. When the reaction was completed, the mixture was cooled to room temperature, diluted with water and then extracted with ethyl acetate. The combined organic layer was washed with water, brine, dried over $MgSO_4$, and concentrated under reduced pressure. The crude product was purified by column chromatography on silica gel (PE/EA = 1/9) to afford compound **6** as a white solid (1.7 g, yield 76%). LC-MS m/z: calculated for $C_{23}H_{26}ClFN_4O_4$ $[M+H]^+$ 477.2, found: 477.3. ^{1}H-NMR (400 MHz, DMSO-d_6) δ 9.56 (s, 1H), 8.50 (s, 1H), 8.14 (dd, *J* = 6.8, 2.6 Hz, 1H), 7.86 - 7.75 (m, 2H), 7.45 (t, *J* = 9.1 Hz, 1H), 7.19 (s, 1H), 6.93 (s, 1H), 4.16 (t, *J* = 6.2 Hz, 2H), 3.98 (s, 3H), 3.14 (q, *J* = 6.5 Hz, 2H), 1.97 - 1.87 (m, 2H), 1.39 (s, 9H).

Synthesis of 7-(3-aminopropoxy)-N-(3-chloro-4-fluorophenyl)-6-methoxyquinazolin-4-amine (**7**). A solution of compound **6** (1 g, 2.65 mmol) in dry DCM (5 mL) was added TFA (5 mL). The reaction mixture was stirred at room temperature for 1 h. The reaction mixture was monitored by LC-MS. When the reaction was completed, the solvent of the reaction mixture was evaporated, affording a white solid that was dissolved in water. After addition of potassium carbonate solution, the solution pH was adjusted to 9-10, then it was extracted with chloroform (3x). The organic layer was washed with water, brine, dried over $MgSO_4$, and concentrated under reduced pressure to afford the crude compound **7** as a white solid (700 mg, yield 75%). LC-MS m/z: calculated for $C_{18}H_{18}ClFN_4O_2$ $[M+H]^+$ 377.1, found: 377.2. ^{1}H-NMR (400 MHz, DMSO-*d*$_6$) δ 9.95 (s, 1H), 8.47 (s, 1H), 8.22 (dd, *J* = 6.9, 2.5 Hz, 1H), 8.04 (s, 1H), 7.91 (ddd, *J* = 8.9, 4.1, 2.7 Hz, 1H), 7.40 (t, *J* = 9.1 Hz, 1H), 7.18 (s, 1H), 4.22 (t, *J* = 6.1 Hz, 2H), 3.97 (s, 3H), 2.92 (t, *J* = 7.2 Hz, 2H), 2.10 - 2.00 (m, 2H).

Synthesis of 7-((3-((4-((3-chloro-4-fluorophenyl)amino)-6-methoxyquinazolin-7-yl)oxy)propyl)amino)-N,N-dimethylbenzo[c][1,2,5]oxadiazole-4-sulfonamide (**9**, HX103). To a solution of compound **7** (85 mg, 0.22 mmol), 7-fluoro-*N*,*N*-dimethylbenzo[c][1,2,5]oxadiazole-4-sulfonamide (**8**) (50 mg, 0.2 mmol) in acetonitrile (2 mL) was added Et_3N (22 mg, 0.22 mmol) and the reaction mixture was stirred at room temperature for 1 h. The reaction mixture was monitored by LC-MS. The reaction was quenched with water and exacted with ethyl acetate (3x). The combined organic layer was washed with water, brine, dried over $MgSO_4$, and concentrated under reduced pressure. The residue was purified by *Prep*-HPLC to obtain **9** as a yellow solid (30 mg, yield 23%). HRMS [ESI+] m/z: calculated for $C_{26}H_{25}ClFN_7O_5S$ $[M+H]^+$ 602.1389, found: 602.1378. ^{1}H-NMR (400 MHz, DMSO-*d*$_6$) δ 9.56 (s, 1H), 8.52 - 8.38 (m, 2H), 8.11 (dd, *J* = 6.8, 2.6 Hz, 1H), 7.85 - 7.71 (m, 3H), 7.43 (t, *J* = 9.1 Hz, 1H), 7.18 (s, 1H), 6.36 (d, *J* = 8.3 Hz, 1H),

4.26 (t, *J* = 5.9 Hz, 2H), 3.93 (s, 3H), 3.59 (d, *J* = 5.6 Hz, 2H), 2.64 (s, 6H), 2.26 - 2.14 (m, 2H). ^{13}C NMR (101 MHz, DMSO-d_6) δ 156.47, 154.82, 154.03, 153.12, 152.41, 149.51, 147.43, 146.98, 144.85, 141.85, 140.87, 137.27 (d, *J* = 3.0 Hz), 123.91, 122.76 (d, *J* = 6.8 Hz), 119.25 (d, *J* = 18.4 Hz), 117.02 (d, *J* = 21.6 Hz), 109.22, 108.39, 105.53, 102.23, 66.74, 56.74, 40.62, 40.50 (d, *J* = 21.0 Hz), 37.90, 27.75.

Measurement of optical properties

In the study, UV-vis spectra of the probes were determined on a Hitachi U-2910. The fluorescence spectroscopic experiments were performed on BioTek microplate reader and Horiba Jobin Yvon-Edison Fluoromax-4. Absolute fluorescence quantum efficiency was measured by an absolute quantum yield spectrometer (Horiba Jobin Yvon-Edison Fluoromax-4). The tested probe was dissolved in dimethyl sulfoxide (DMSO) to obtain stock solutions (10 mM). The prepared stock solution was diluted in sodium phosphate buffer (PBS, 1 M, pH=7.4) to obtain the desired final concentration solution, containing 0.1% (v/v) DMSO as a cosolvent. For the *in vitro* kinase reaction, the probes were diluted with PBS to obtain final concentrations of 5 μM. The excitation and emission fluorescence spectra of the solution before/after addition of different concentration of EGFR wild-type or EGFR mutants (0-6 μM) at room temperature for 30 min was measured. *Data analysis*：The obtained fluorescence intensity at 570 nm (Excitation wavelength was 440 nm) in each sample was normalized by using the fluorescence intensity of HX103 in the presence of EGFR. Specifically, the fluorescence intensity of HX103 in the presence of EGFR wild-type was set at 100% to normalized that of the remaining samples.

Cytotoxicity assays

The cytotoxicity of the tested compounds against selected cell lines was measured by 3-(4,5-dimethylthiazol-2-yl)-2,5-diphenl tetrazolium bromide (MTT) assay. Briefly, cells (3000 per well) were plated in 96-well plate for 24 h and 100 µL of medium with different concentrations of the tested compounds was added to each well for 48 h. Then 20 µL of MTT (5 mg/mL, Sigma-Aldrich) was added and incubate at 37 ℃ for 4 h, resulting the formation of the formazan crystal. The formed formazan crystal was dissolved with 150 µL of DMSO after removing the medium. The absorbance of each well was measured at 570 nm wavelength. The inhibition rate was calculated by the formula: $(OD_{\text{control group}} - OD_{\text{treated group}})/OD_{\text{control group}} \times 100\%$. The experiments were repeated at least three times. The IC_{50} values were determined by plotting a log (inhibitor) vs. normalized response (Variable slope) dose-response curve generated using GraphPad Prism software.

Immunohistochemistry staining

The obtained tumor tissues were sectioned into about 3 µm thickness layers. The general protocol of immunohistochemistry (IHC) was applied for the experiment. Firstly, the tissue slices were deparaffinized in xylene before immersion in 100% ethanol, followed by antigen repair. Then, the sections were placed in 10 mM citrate buffer (pH 6.0) at 96 ^{o}C for 15 min, followed by incubation with primary anti-EGFR (L858R specific, 1:100) at 4 ^{o}C for overnight. The sections were washed by PBS for 5 min (2 times) and then incubated with the secondary antibody for 30 min at 37 ^{o}C, followed by washing with PBS and reacting with DAB kit (~2 min). The reaction was quenched by the addition of water, the sections were subsequently stained by haematoxylin and differentiated

by hydrochloric alcohol. Finally, the tissue sections were fixed and the images were obtained using Nikon (Ni-E) microscope and were analysed by Image J software.

Quantitative RT-PCR analysis

The mRNA expression of EGFR in cultured cancer cells was determined by quantitative real-time PCR. For all samples, 1~4 × 10^6 cells per sample were harvested and total cellular RNAs were extracted with TRIzol^{TM} reagent (#15596026, Invitrogen, USA) according to the manufacture's instruction. RNA integrity was detected using the electrophoresis on a denaturing agarose gel and RNA purity and concentration was determined by NanoDrop 1000 spectrophotometer (NanoDrop Technologies, USA). Subsequently, RNA was reverse transcribed to cDNA using the Hiscript III All-in-one RT SuperMix Perfect for qPCR Kit (#R333-01, Vazyme, China). Subsequent qPCR analysis was performed on the 96-well (0.2 mL, block) real-time PCR system (Applied Biosystems, QuantiStudio 3). ChamQ Universal SYBR qPCR Master Mix Kit (#Q711, Vazyme, China) was used to quantify the target genes with GAPDH as an internal standard, the program was set as follows: 95 ^{o}C for 30 s, 35 cycles at 95 ^{o}C for 10 s and 60 ^{o}C for 30 s. All samples were determined in triplicates. The expression of EGFR genes was normalized to GAPDH and the relative expression of EGFR was analyzed using the $2^{-\Delta\Delta Ct}$ method. GAPDH was used for background correction. The primers used in this study are listed as follow: 1) GAPDH, 5'-GTGAAGGTCGGAGTCAACG-3' (forward), 5'-GGTGAAGACGCCAGTGGACTC-3' (reverse); 2) EGFR, 5'-TTGCCGCAAAGTGTGTAACG-3' (forward), 5'-GTCACCCCTAAATGCCACCG-3' (reverse).

Droplet digital PCR (ddPCR) analysis

DNA collection: Genomic DNA extraction from specimens of surgically resected tissues was performed using the Ezup Column Animal Genomic DNA Purification Kit (B518251, Sangon Biotech). DNA mass was measured using the NanoDrop 2000 (Thermo Fisher Scientific, USA), as the manufacturer's recommendation. The extracted DNA was stored at -80 ^{o}C until use.

Droplet digital PCR assays were performed on a Sniper DQ24 digital droplet PCR system (Sniper, China), which combines water-oil emulsion droplet technology with microfluidics. Briefly, the extracted DNA was compartmentalized into thousands of droplets. Each droplet was PCR amplified and fluorescently labeled. Primers and probes for EGFR mutations were based on previous studies [1], For *EGFR* L858R, the primer sequences were as follow: forward primer, 5'-GCAGCATGTCAAGATCACAGATT-3', reverse primer, 5'-CCTCCTTCTGCATGGTATTCTTTCT-3'. Probe sequences: 5'-VIC-AGTTTGGCCAGCCCAA-MGB-NFQ-3', 5'-FAM-AGTTTGGCCCGCCCAA-MGB-NFQ-3'. For *EGFR* 19del, the primer sequences were as follow: forward primer, 5'-GTGAGAAAGTTAAAATTCCCGTC-3', reverse primer, 5'-CACACAGCAAAGCAGAAAC-3'. Probe sequences: 5'-VIC-ATCGAGGATTTCCTTGTTG-MGB-NFQ-3', 5'-FAM-AGGAATTAAGAGAAGCAACATC-MGB-NFQ-3'. All the primers and probes were synthesized by Sangon Biotech. PCR cycling condition were as follow: for *EGFR* 19del, 60 ^{o}C for 5min and 95 ^{o}C for 15 min, 40 cycles of 94 ^{o}C for 30 s and 55 ^{o}C for 1 min, then take picture at 60 ^{o}C; for *EGFR* L858R, 60 ^{o}C for 5min and 95 ^{o}C for 15 min, 40 cycles of 94 ^{o}C for 30 s and 58 ^{o}C for 1 min, then take picture at 60 ^{o}C. The endpoint fluorescence signal from each droplet was record and the number of positive and negative droplets were read by an automated droplet flow cytometer based on the fluorescent intensity. We analyzed the results using SightPro software. All

data of the droplets from each sample were converted to a two-dimensional scatterplot displaying FAM intensity on the x-axis and VIC intensity on the y-axis after spectral compensation. Thresholds were placed manually and the population of positive and negative droplets were used to identify the concentration and fractional abundance of target DNA sequences.

Multicolor FACS analysis in clinical samples

The obtained tissues were dissociated into single-cell suspension using a standard protocol of primary cell isolation. Briefly, the obtained fresh tissue was placed in a petri dish on ice and cut into small pieces of ~2 mm. The pieces were transferred into a centrifuge tube (15 mL) containing the enzyme mixture of collagenase I (1 mg/mL, #17100017, Gibco) and IV (1 mg/mL, #17104019, Gibco), and digested for 30 min at 37 °C under gentle agitation. Subsequently, the digested sample was filtered through 70-μm and 40-μm cell strainer (Falcon), respectively, followed by washing with serum-free medium (DMEM). The tumor cell pellet was then obtained by the centrifugation at 500 g for 5 min at 4 ^{o}C for the removal of the supernatant. Then, the cell pellet was resuspended in serum-free medium and 1 mL of red blood cell lysis buffer was added and incubated for 5 min at 4 ^{o}C to remove the erythrocyte. The cells were pelleted and resuspended in serum-free medium and ready for FACS analysis [2, 3]. The dissociated cells were pelleted (800 g, 4 min, 4 ^{o}C) and resuspended in PBS (containing 1% BSA). Subsequently, 2 μL of live/DEAD cell discriminator kit (#130-091-163, Miltenyi Biotec) was added (1×10^7 cells per tube) on ice for 10 min under a light source to label the dead cells. Cells were then washed with PBS (containing 1% BSA) (100 μL per 10^7 cells, centrifugation at 1500 g, 3 min), followed by the addition of discriminator stop reagent into the cells. Subsequently, the cells were pelleted (1000 g, 3 min) and resuspended in PBS buffer (at a concentration of 1×10^6 cells/mL).

To start the labeling experiment, 499 μL of the isolated cell suspension was used per sample and 0.5 μL gefitinib or DMSO was added to each cell suspension and incubated for 30 min at 37 ℃. HX103 (final concentration of 5 μM) or DMSO was added, incubated for 30 min at 37 ^{o}C. The treated cells were pelleted and fixed with 200 μL of 4% paraformaldehyde (PFA) for 10 min, washed and resuspended in PBS buffer, followed by blocking with human BD Fc block for 20 min. The cells were pelleted (2000 g, 3 min) to remove the supernatant and co-stained with 50 μL of an antibody cocktail [CD45 Pacific blue (1:200), Alexa Fluor 647 anti-EGFR (1:100)] or the isotype control (Alexa fluor 647 Rabbit IgG, ab199093, abcam, 1:100). The co-staining was performed for 30 min at 37 ^{o}C, then the cells were pelleted (2000 g, 3 min) and washed with PBS buffer and PBS (containing 1% BSA) (2 times). The cells were pelleted (2000 g, 3 min) and resuspended in PBS (containing 1% BSA) and ready for analysis with a FACSVerse^{TM} flow cytometer (BD Biosciences, LSRFortessa) (the lasers used for this study: 405 nm, 488 nm and 647 nm). Dead cells were excluded based on FSC/SSC, and the CD45(+) cells were excluded by gating at the CD45-cell population (CD45-gate). The mean fluorescent intensity (MFI) and the ratio of EGFR(+)HX103(+) cells were analyzed with FlowJo software 10.1. The thresholds for specific signals were set according to DMSO-treated sample. The cells were analyzed with a FACSVerse^{TM} flow cytometer (BD Biosciences, LSRFortessa). 10000 events per sample were analyzed.

Data analysis：Gating was set to exclude the cell debris, which is identified by forward and side scatter properties (FSC/SSC) on a density plot. To be able to detect tumor cells, an electronic threshold level of 10000 for the signal of the forward scatter was applied, and the CD45(+) normal T lymphocytes were excluded by gating at the CD45(-) cell population (CD45-gate). Thus, cellular debris and CD45(+) cells with background fluorescence signals below the threshold values were not detected by the flow cytometer. The thresholds for specific EGFR (APC) and HX103 (FITC)

signals were set according to DMSO-treated and isotype-treated samples. Gates were set to have no positive events above these thresholds in DMSO-treated sample (Fig. 4a). HX103 positive cells were selected by FITC fluorescence intensity (Q2+Q3) and EGFR positive cells were selected by APC fluorescence intensity (Q1+Q2). The definite population of double positive cells (Q2) were calculated from positive populations (background corrected by DMSO-treated sample). The calculated data was described using the percentage of cell populations and mean fluorescence intensity (MFI) values shown in Supplementary Table 5 and 6, respectively. The MFI of DMSO-treated samples was subtracted from the MFI values of HX103 to obtain background-corrected specific MFI for individual samples. The MFI values of DMSO-treated sample was also subtracted from the MFI in competition experiments. The background-corrected MFI of HX103 was set at 100% to normalize the background-corrected MFI of other samples.

Supplementary References

1. Thress KS, *et al.* Acquired EGFR C797S mutation mediates resistance to AZD9291 in non-small cell lung cancer harboring EGFR T790M. *Nat. Med.* **21**, 560-562 (2015).

2. Hristozova T, Konschak R, Budach V, Tinhofer I. A simple multicolor flow cytometry protocol for detection and molecular characterization of circulating tumor cells in epithelial cancers. *Cytometry A.* **81**, 489-495 (2012).

3. Soethoudt M, *et al.* Selective photoaffinity probe that enables assessment of cannabinoid CB2 receptor expression and ligand engagement in human cells. *J. Am. Chem. Soc.* **140**, 6067-6075 (2018).

Supplementary Notes

Observational Study Protocol

1. Research background

Lung cancer is the malignant tumor with the highest morbidity and mortality worldwide, with a 5-year survival rate of only 16% [1]. According to statistics, lung cancer is the cancer with the highest incidence among male malignant tumors in many countries, accounting for 21.9% of the total incidence. Among female malignant tumors in our country, lung cancer ranks second with 13.3% [2-3]. 80%-85% of lung cancer are non-small cell lung cancer (NSCLC) [4]. Compared with small cell lung cancer (SCLC), NSCLC grows and divides slowly, and spreads and metastasizes later. The diagnosis time of lung cancer is particularly important for the advanced lung cancer patients. Despite the continuous development and improvement of medical technology, most of the patients diagnosed with NSCLC at the current stage are in the advanced stage [5], and it is difficult to perform surgery and other methods. In addition, although some patients are fortunate to be diagnosed at an early stage and receive corresponding treatments, such as surgery, radiotherapy, and adjuvant chemotherapy, they still have local recurrence or distant metastasis in the end. Therefore, the five-year survival rate of NSCLC has not been greatly improved in recent years.

In recent years, the discovery of targeted drugs is a major breakthrough in the clinical treatment of lung cancer, which can effectively prolong the survival period of lung cancer patients (up to 24 months) [6]. Among them, epidermal growth factor receptor-tyrosine kinase inhibitor (EGFR-TKI) targeted drugs are one of the important anti-tumor targeted drugs. Studies have shown that without any choice, the overall treatment efficiency of EGFR-TKI for NSCLC patients

is about 10% to 20%, but for patients with epidermal growth factor receptor (EGFR) mutants, the effective rate can be as high as 60%. %～80%). Therefore, accurate detection of lung cancer with EGFR mutations is the key to the targeted drugs for lung cancer treatment. Clinically, the EGFR gene mutation status must be tested before using TKI-targeted drugs for patients with non-small cell lung cancer. However, it is difficult to achieve accurate detection of EGFR mutation in lung cancer patients with the current existing technical methods. The mean challenges are as follows: 1) First, lung cancer has complex heterogeneity, including individual heterogeneity, spatial heterogeneity and temporal heterogeneity. Individual heterogeneity means that different lung cancer patients have great differences in their EGFR mutations; spatial heterogeneity means that their EGFR mutation varies in different lesions and at different locations in the same lesion; temporal heterogeneity means that there is a dynamic difference in EGFR mutations during lung cancer progression. Therefore, real-time, dynamic and accurate detection of EGFR mutation status can solve the problem of lung cancer heterogeneity. 2) Secondly, the existing EGFR mutation detection methods have certain limitations. For example, the use of pathological methods to detect EGFR mutations has poor reproducibility and cannot overcome the problems of spatial heterogeneity. In addition, the use of traditional imaging methods such as CT and magnetic resonance cannot provide the distributions of EGFR. Therefore, to achieve accurate diagnosis and treatment of lung cancer, it is urgent to develop a new technology to quantitively detect EGFR mutation in protein expression level.

Molecular probes enable to detect specific cells and gene expression processes in real time, and to track protein targets (e.g., imaging, measurement), to achieve real-time and dynamic assessment of disease progression. Therefore, a highly specific molecular probe targeting EGFR can qualitative and quantitative detect EGFR. This project aims to develop molecular probes based

on EGFR-TKIs to achieve the quantitative detection and visualization of EGFR mutations in lung cancer.

2. Aim of the study

(1) The major aim: our major aim of the study is to identify the subset of patients who may benefit from EGFR-TKIs therapy, and hence to design appropriate treatment strategies for optimized clinical outcome. Specifically, the biopsied samples of lung cancer patients are subjected to quantitative detection of EGFR mutations (L858R, 19del) using the fluorescent probe HX103 and FACS analysis. Combined with the results from gene sequencing and HX103 labeling, it will allow us to rapid and accurate determine EGFR mutations in lung cancer patients.

(2) Secondary aim: we will also evaluate the possibility of the EGFR fluorescent probe HX103 in clinical translational application, particularly the quantitative information of HX103 labeling in discrimination of EGFR mutations and predicting the sensitivity of EGFR-TKIs therapy in NSCLC patients.

3. Design, methods and procedures of the study

(1) Study design

This study is a prospective study and will be performed at a single research center in China (West China Hospital of Sichuan University). The acquisition of biopsied samples will be accepted by the patients. The fluorescent probe and FACS will be used to detect EGFR mutations at the Targeted Tracer Research and Development Laboratory of West China Hospital. This study will be performed as follows:

1) Firstly, patients with advanced lung cancer diagnosed by pathology in West China Hospital

were selected. All the participants who were accordance with the diagnosis criteria of lung cancer (stage III, IV) need to sign informed consent after told study details.

2) Using gene sequencing analysis to detect EGFR mutation status for all the participants.

3) The biopsied tissues were obtained by the qualified pulmonary physicians. The obtained tissues were immediately prepared into single-cell suspensions, which will be used to quantitatively detect EGFR mutation with HX103 using FACS.

4) Identifying patients who may benefit from EGFR-TKIs therapy and designing appropriate EGFR-TKIs treatment strategies. Considering our study is a non-interventional clinical study, we will design EGFR-TKIs treatment strategies based on gene sequencing results in accordance with the "Guidelines for the Clinical Use of Targeted Drugs for Lung Cancer ".

5) After receiving EGFR-TKIs treatments, the participants will return to the research center for prognostic evaluation of EGFR-TKIs efficacy (CT test) (approximately every 8-10 weeks). Furthermore, to ensure the safety of participants after TKIs therapy, we will keep in touch with the participants with telephone. The follow-up time is 4 months to 6 months, even longer (if possible).

6) Comparing the CT results (pre-TKIs and post-TKIs) of the participants between gene sequencing and HX103 labeling results, and evaluating the accuracy of EGFR mutation detection by HX103 labeling and EGFR-TKIs therapy efficacy.

(2) Methods of the study

In general, this study will use statistical analysis to systematically analyze the feasibility of EGFR mutation discrimination using HX103 labeling determined by FACS analysis, comparing with the reports of gene sequencing analysis. For gene sequencing analysis, this study will use

both Sanger sequencing and next-generation sequencing analysis to detect EGFR mutation status. For determining the amount of HX103 labeling in biopsied samples, we will apply multicolor FACS analysis, which is previously optimized by our group, to quantitatively measure the fluorescent labeling of HX103. Subsequently, we will evaluate the sensitivity and selectivity of HX103 labeling to determine EGFR mutation by comparing with gene sequencing analysis. A receiver operating characteristic (ROC) curve analysis will be computed for HX103 labeling, in order to assess the ability of HX103 to determine EGFR mutations. Meanwhile, we will identify the optimum cut-off value of the percentage of HX103 labeling, when discriminating EGFR mutations.

Taking into account that there may be a certain deviation between the gene sequencing analysis and HX103 labeling, and if it happens, how to make an appropriate EGFR-TKIs therapy strategy for the participants? As this study is a non-interventional clinical study, the assignment of the patients to the therapeutic strategy will be decided in accordance with the "Guidelines for the Clinical Use of Targeted Drugs for Lung Cancer in China". Specifically, the EGFR-TKIs treatment strategies will be making according to gene sequencing results. However, HX103 labeling will be referenced in this study, especially, pay attention to compare EGFR-TKIs therapy efficacy with the amount of HX103 labeling. In practice, there are four possibilities, when making EGFR-TKIs treatment strategy:

(A) If the gene sequencing and HX103 labeling both show the exist of EGFR mutation (L858R or 19del), the patient will be selected to EGFR-TKIs treatment;

(B) If the gene sequencing shows the exist of EGFR mutation (L858R or 19del), but not HX103 labeling, the patient will be selected to EGFR-TKIs treatment in accordance with the "Guidelines for the Clinical Use of Targeted Drugs for Lung Cancer in China";

(C) If the gene sequencing shows EGFR wild-type, but HX103 shows the exist of EGFR activating mutations, the patient will **not** be selected to EGFR-TKIs treatment in accordance with the "Guidelines for the Clinical Use of Targeted Drugs for Lung Cancer in China";

(D) If the gene sequencing and HX103 labeling both show EGFR wild-type, the patient will **not** be selected to EGFR-TKIs treatment.

After receiving EGFR-TKIs treatments, the participants will return to the research center for prognostic evaluation of EGFR-TKIs efficacy (CT test) (approximately every 8-10 weeks). Comparing the prognosis of EGFR-TKIs therapy between gene sequencing analysis and HX103 labeling, especially, pay attention to the patients with high HX103 labeling.

(3) Procedure of the study

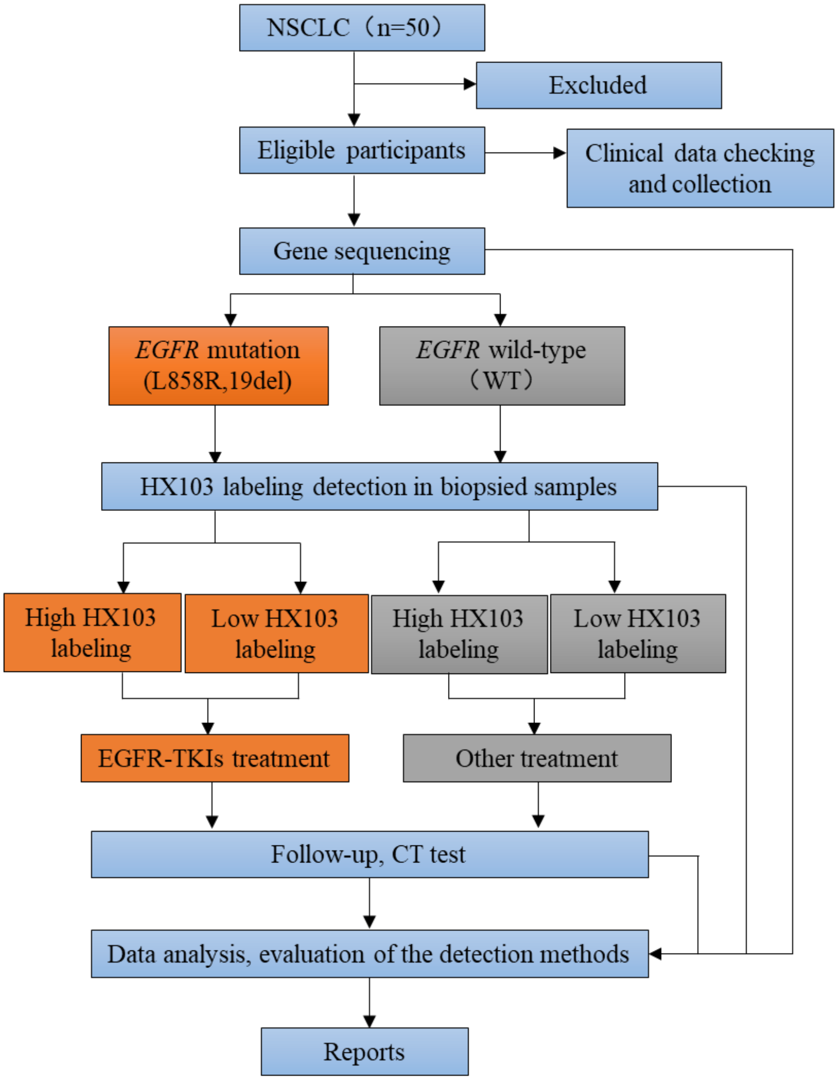

Figure 1. Flow chart of the clinical study

4. Case selection (Explain the rationality and feasibility of the selection of research objects and related standards)

(1) Selection criteria

- Voluntary test, the patient or his/her legal representative signs informed consent;
- Volunteers are ≥ 40 years old;

- Lung cancer patients were enrolled, including EGFR mutation-positive group and EGFR wild-type group (determined by gene sequencing).

(2) Exclusion criteria

- Patients and family members who refuse to participate in the process;
- No detailed clinical data is available;
- Lung cancer patients who have previously received targeted drug therapy.
- There are other clinical problems that are difficult to be controlled (such as HIV, hepatitis C virus infection or active hepatitis B, or other serious chronic infectious diseases, etc.).
- Exclude patients with limited amount of biopsied sample (not enough to produce single-cell suspension for HX103 labeling).

(3) Criteria for termination of the study

If the participate happens to situations that are not suitable for continuing the study, including: worsening of the disease, serious adverse events, poor compliance, etc., the study should be discontinued.

5. Alternative diagnosis and treatment methods

None.

6. Testing items and testing time point (design clinical observation and laboratory inspection items around the main efficacy indicators and secondary efficacy indicators of the study, and also need to consider the observation of safety evaluation indicators, and explain the testing time point)

The enrolled patients were registered at West China Hospital of Sichuan University and received routine testing, CT test, and gene sequencing analysis. With the agreement of the patient, the obtained biopsied sample will be used for detection of EGFR mutations by using HX103 and FACS analysis. According to the results of gene sequencing analysis, as well as referenced to HX103 labeling, we will identify the superior population for EGFR-TKIs therapy. Patients with EGFR mutation-positive determined by gene sequencing and HX103 labeling belong to the best superior population, and those with EGFR mutation positive determined by only gene sequencing also belong to the EGFR-TKIs treatment group. After EGFR-TKIs therapy (every 8-10 weeks), the patients will return to the research center for follow-up assessment of safety and prognosis of EGFR-TKIs efficacy by CT tests. The follow-up time is 4 months to 6 months.

7. Criteria of diagnostic evaluation

- *Interpretation of CT imaging*: detected by a doctor with a professional title of deputy director or above with diagnostic experience;
- *Gene sequencing*: the Precision Medicine Center of West China Hospital will perform the experiments of gene sequencing analysis.
- *Determination of probe performance*: compare the labeling of HX103 with gene sequencing and CT follow-up results and analyze the specificity, sensitivity, and accuracy of the ability of HX103 in detection of EGFR mutation and prediction of EGFR-TKIs sensitivity.

8. Observation, recording and handling of adverse events

(1) After receiving EGFR-TKIs, any adverse reactions or events should be recorded in detail in the adverse event record page of the case report form.

(2) If a serious adverse reaction (SAE) occurs, a preliminary report must be completed within 24 hours and submitted to the department of the hospital's clinical research management and biomedical ethics committee.

9. Research quality control and quality assurance

(1) Probe preparation and quality control to ensure purity > 98%;

(2) The weight of the biopsied sample used for the probe labeling will be kept within a certain range;

(3) Strict registration and recording of data collection, analysis, etc., and proceed in accordance with the regulations of the research plan, and the data can be traced back;

(4) The informed consent of the participant is fully informed to ensure compliance;

10. Data Security Supervision

The clinical research will develop a corresponding data security monitoring plan based on the level of risk. All adverse events are recorded in detail, properly handled and tracked until they are properly resolved or the condition is stable, and report serious adverse events and unexpected events to the ethics review committee, competent authorities, sponsors and drug regulatory authorities in a timely manner in accordance with regulations. The main investigator regularly carry out a cumulative review of all adverse events, and if necessary, hold a meeting of researchers

to evaluate the risks and benefits of the study; if necessary, double-blind trials can be urgently unblinded to ensure the safety and rights of subjects.

11. Statistical processing

All statistical analysis, summary tables and lists use Excel and GraphPad Prism 6. Standard descriptive summary includes N, mean, median, standard deviation or coefficient of variation (% CV); minimum and maximum values of continuous variables; number and percentage of categorical variables. According to the gene sequencing and HX103 labeling, the receiver operating characteristic curve (ROC) is produced, and the area under the curve (AUC) value is calculated, which will be used to evaluate the sensitivity, specificity and accuracy of the detection of EGFR mutation/EGFR-TKI sensitivity by HX103 labeling.

12. Ethical principles and requirements of clinical study

Clinical study will follow the relevant regulations of the World Medical Congress "Declaration of Helsinki" and the National Health and Family Planning Commission of the People's Republic of China "Measures for the Ethical Review of Biomedical Research Involving Humans", and implement informed consent, protect privacy, research free and compensation, and control risks, protection of special subjects and compensation principles and requirements for research-related damages. Before the start of the study, the clinical study was carried out after the ethics review committee approved the trial protocol. Before each subject is selected for this study, the investigator has the responsibility to fully and comprehensively introduce the purpose, procedures and possible risks of the study to the subject or/and his legal representative, and sign a written informed consent form. Let the subjects know that their participation in the clinical research is completely voluntary. They can refuse to participate or withdraw from the research at

any stage of the trial without discrimination and retaliation, and their medical treatment and rights will not be affected. The informed consent form should be kept as a clinical research document for future reference to effectively protect the subject's personal privacy and data confidentiality.

13. Research progress

January 2019-December 2019: Preparation materials, probe preparation.

January 2020- October 2021: Patients are enrolled and research is carried out.

November 2021-December 2021: Summary data, complete report

14. Reference

1. Zhou M, Wang H, Zeng X, et al. Mortality, morbidity, and risk factors in China and its provinces, 1990-2017: a systematic analysis for the Global Burden of Disease Study 2017 [J]. Lancet, 2019, 394(10204): 1145-1158. DOI: 10.1016/S0140-6736(19)30427-1.

2. Bray F, Ferlay J, Soerjomataram I, et al. Global cancer statistics 2018: GLOBOCAN estimates of incidence and mortality worldwide for 36 cancers in 185 countries [J]. CA Cancer J Clin, 2018, 68(6): 394-424. DOI:10.3322/caac.21492.

3. Feng RM, Zong YN, Cao SM, et al. Current cancer situation in China: good or bad news from the 2018 Global Cancer Statistics? [J]. Cancer Commun (Lond), 2019, 39(1): 22. DOI: 10.1186/s40880-019-0368-6.

4. Didkowska J, Wojciechowska U, Mańczuk M, et al. Lung cancer epidemiology: contemporary and future challenges worldwide [J]. Ann Transl Med, 2016, 4(8): 150. DOI: 10.21037/atm.2016.03.11.

5. Sant M, Aareleid T, Berrino F, et al. EUROCARE-3: survival of cancer patients diagnosed 1990-94--results and commentary [J]. Ann Oncol, 2003, 14 Suppl 5: v61-118. DOI:10.1093/annonc/mdg754.

6. Mok TS, Wu YL, Thongprasert S, et al. Gefitinib or carboplatin-paclitaxel in pulmonary adenocarcinoma [J]. N Engl J Med, 2009, 361(10): 947-957. DOI: 10.1056/NEJMoa0810699.
